# Electronic Tuning of Host-Guest Interactions within the Cavities of Fluorophore-Appended Calix[4]arenes

**DOI:** 10.3390/molecules27175689

**Published:** 2022-09-03

**Authors:** Varun Rawat, Arkadi Vigalok

**Affiliations:** School of Chemistry, The Raymond and Beverly Sackler Faculty of Exact Sciences, Tel Aviv University, Tel Aviv 69978, Israel

**Keywords:** calixarene, chemosensors, fluorescence, molecular recognition, π interactions

## Abstract

A series of fluorescent calix[4]arene scaffolds bearing electron-rich carbazole moiety conjugated at the lower rim have been prepared. Studies of the fluorescence quenching in the presence of the N-methyl pyridinium guest revealed that the electronic properties of the distal phenolic ring play a major role in the host–guest complexation. In particular, placing an electron-donating piperidine fragment at that ring significantly increased the host–guest interactions, while introducing the same fragment into the proximal phenolic ring weakened the fluorescence response. These results suggest that the dominant interactions between the guest and calixarene cavity involve the oxygen-depleted fluorophore-bearing aromatic ring and not the more electron-rich unsubstituted phenolic fragments.

## 1. Introduction

**Figure 1:**
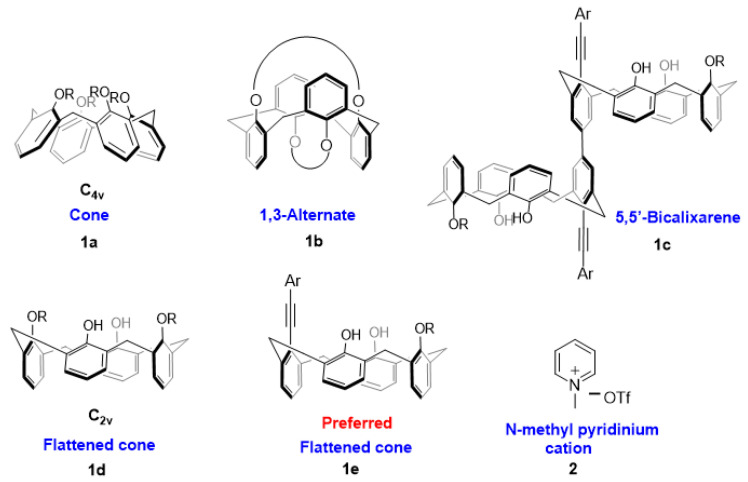
Representative calixarene scaffolds for host–guest chemistry.

Complexation of cationic organic guests within the electron-rich cavity of calix[4]arene (calixarene) compounds has been at the heart of the host–guest complexation chemistry for several decades [1]. In particular, multiple calixarene scaffolds have been investigated in much detail with regard to the complexation of various pyridinium salts and their derivatives [2]. In addition to common calixarene hosts, these studies involved calixarene scaffolds adapting various conformations [3], and scaffolds containing two calixarene cavities (Figure 1) [4]. In the great majority of the studies, ^1^H NMR spectroscopy was the method of choice to determine the strength of the host–guest complexation [5], with the technique typically requiring relatively high concentrations. Surprisingly, to our knowledge, studies on common electronic effects on this complexation reaction have not been reported. While the introduction of electron-donating or -accepting substituents in the calixarene aromatic rings can be viewed as a judicious, albeit synthetically challenging, route to study these effects, the data analysis can be skewed by the conformational changes of the host molecule with regard to the guest cation. It is generally accepted that π interactions (cation-π and/or π-stacking) play an important role in the overall complexation of N-alkyl pyridinium salts within the calixarene hosts [6]. With nearly all studied calix[4]arene hosts having four alkyl ether groups at the lower rim, the average C4v conic structure of the cavity is not optimized for such interactions [7,8,9,10,11,12,13,14,15,16]. Naturally, π interactions would be maximized if a pair of the opposite aromatic groups adopted a parallel disposition, where two opposite aromatic groups are parallel to each other in a C_2v_ symmetrical conformation (**1d**, flattened cone), which for the symmetrically substituted calixarenes can be observed only at low temperatures [16,17,18]. A straightforward way to achieve such an arrangement is the selective 1,3-lower rim dialkylation or acylation of the phenolic oxygens, leading to the protected phenolic moieties adopting a parallel geometry. Alternatively, a replacement of an oxygen atom at the lower rim with a non-polar hydrocarbyl group also results in the oxygen-depleted (formerly) phenolic fragment becoming aligned with the opposite phenolic ring, as can be deduced from the available structural data (**1e**) [19,20]. Interestingly, although these parallel aromatic rings are pre-arranged to participate in π interactions, they are less electron rich than the remaining unsubstituted phenolic rings which can adopt a similar arrangement by sacrificing the stabilizing hydrogen bonding between the OH groups. Distinguishing between the two different binding modes could be aided by studying electronic effects of appropriate substituents on the cation complexation. Surprisingly, no such studies have been reported to the best of our knowledge.

**Figure 2:**
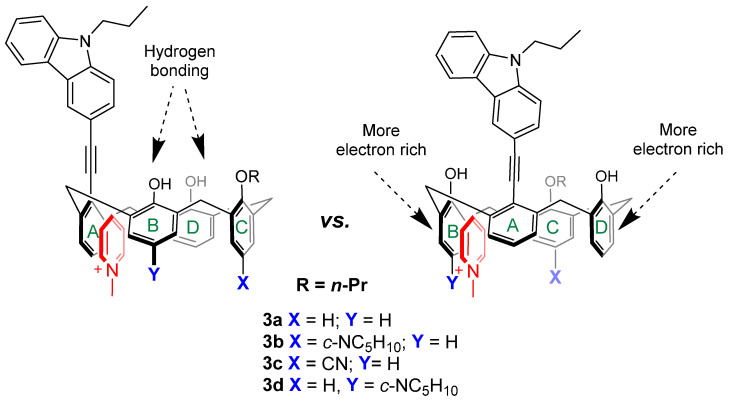
Proposed π interactions between **2** and carbazole-appended calixarenes **3**.

While studying the chemosensory properties of oxygen-depleted 5,5′-Bicalixarene scaffolds (**1c**) bearing an alkyne function at the lower rim [21], we discovered that these compounds show strong NMR and fluorescence response upon the complexation of N-methyl pyridinium cation (**2**) [20,22]. Attachment of electron-donating fluorophores at the termini of the bicalixarene fragment expectedly increased the host–guest complexation properties of the scaffolds [23]. Yet, this observation alone does not provide compelling evidence for the π interactions with the oxygen-depleted part of the calixarene moiety. Here, we present our studies of model calixarene compounds that support the notion of the π interactions between the parallel opposing aromatic rings and N-methyl pyridinium cation playing major role in the host–guest complexation (Figure 2).

## 2. Results and Discussion

Although the presence of the electron-donating fluorophores at the termini of the biphenyl chain in **1c** increases the fluorescence response to the host–guest interactions with **2**, there is no evidence for this chain being involved in the π interactions. Because the adjacent free phenolic rings are more electron rich, they potentially can provide stronger π stabilization to the cationic aromatic guest. As stated above, such strong stabilization would come at the cost of breaking hydrogen bonding between the phenolic groups at the lower rim. To establish the pair of the opposing aromatic rings being responsible for the π interactions with **2**, we decided to directly compare its complexation within the cavities of the substituted mono calixarene hosts **3** (Figure 2).

**Scheme 1:**
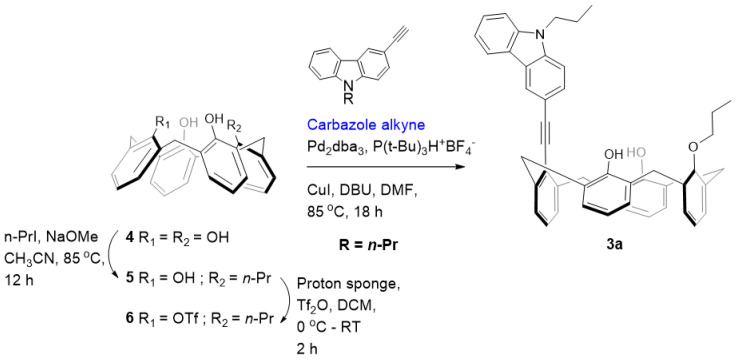
Synthesis of compound **3a**.

**Scheme 2:**
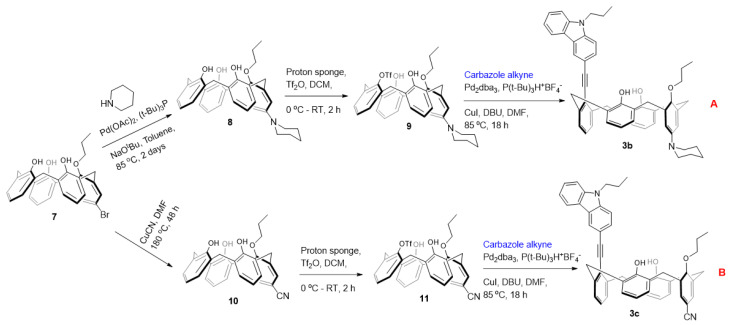
Synthesis of compounds **3b,c**.

**Scheme 3:**
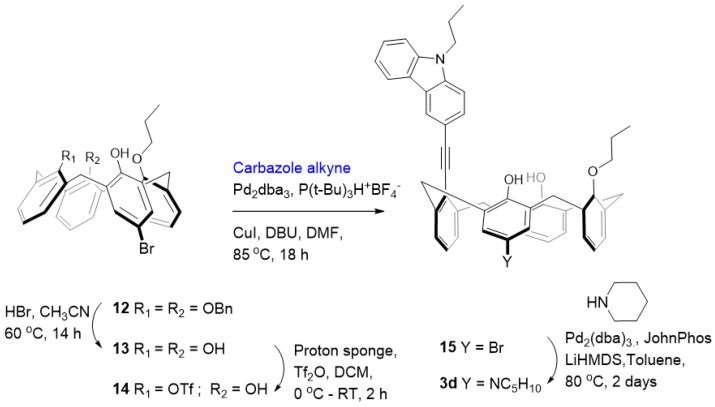
Synthesis of compound 3d.

We hypothesized that if the fluorophore-appended ring A is involved in the π interactions, the substituents in ring C should have major effect on the complexation of **2**. On the other hand, if the phenolic rings B and D are the main contributors to the π interactions, substitution in ring B will cause some change in the fluorescence response. To verify this hypothesis, we developed synthetic protocols toward unsymmetrically substituted hosts **3a–d** (Scheme 1, Scheme 2 and Scheme 3). Moreover, although we earlier reported the synthesis of the parent compound **3a** (Φ = 0.17) [23], we have now modified the procedure to obtain the desired compound in only three steps (Scheme 1).

To prepare compounds **3b** and **3c**, bearing, at the C ring, the electron-donating piperidine group and electron-withdrawing cyano group, respectively, the corresponding bromo-derivative **7** was prepared in three steps [23]. Reacting compound **7** with piperidine under the Buchwald–Hartwig amination conditions afforded the amino derivative **8** which was converted to the triflate **9**. The Sonogashira coupling with the carbazole alkyne gave **3b** in a 14% overall yield and quantum yield of 30% (Φ = 0.30) (Scheme 2A) [24].

**Figure 3:**
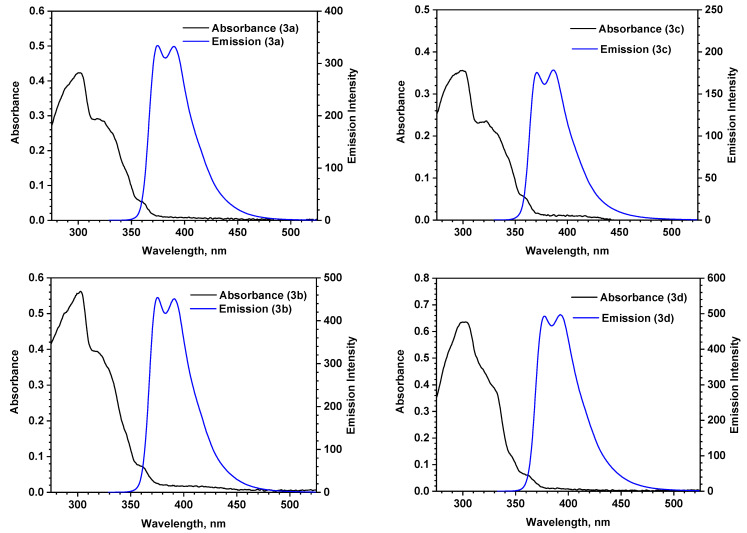
Absorbance–emission spectra of compounds **3a**–**d**.

For **3c** (Φ = 0.14), compound **7** was converted to the cyano derivative **10** via the Rosenmund–von Braun reaction with CuCN, followed by the similar protocols for the installation of the carbazole group at the lower rim (Scheme 2B). To prepare compound **3d**, the selective protection of the phenolic groups on rings A, D, and C was performed followed by the bromination at the para- to the OH position of the remaining unprotected ring B (compound **12**) [25,26]. The removal of the benzylic groups and selective triflation of the intermediate **13** produced the triflate **14** which was reacted with **4**. Finally, the obtained compound **15** was converted to **3d** (Φ = 0.39) via Buchwald–Hartwig amination with piperidine (Scheme 3) [27]. All new compounds were fully characterized by the multinuclear NMR spectroscopy and HRMS. All compounds **3a–d** exhibit strong fluorescence upon irradiation with the UV light (Figure 3).

**Figure 4:**
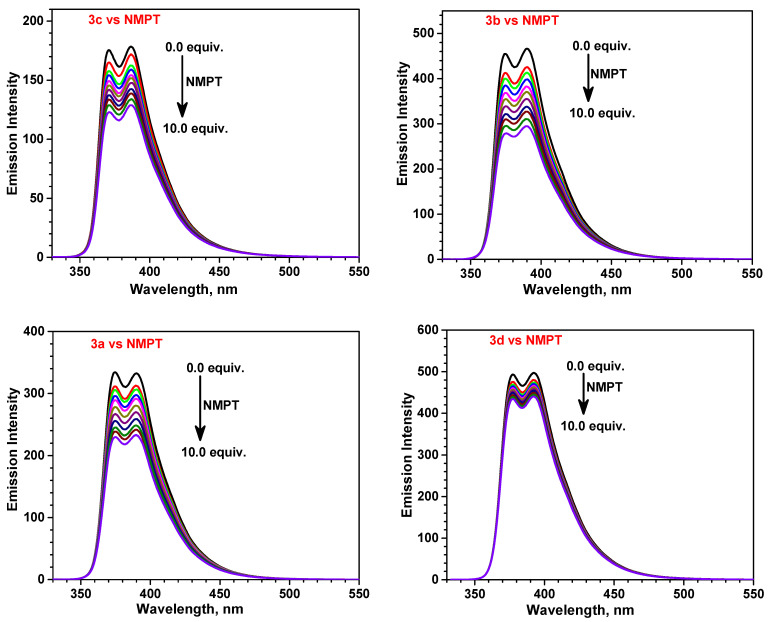
Fluorescent spectra of carbazole-appended calixarenes **3a**–**d** (DCE, 10 μM) with various concentrations of NMPT, **2** (0 to 10 equivalents). Excitation wavelength 321 nm.

**Figure 5:**
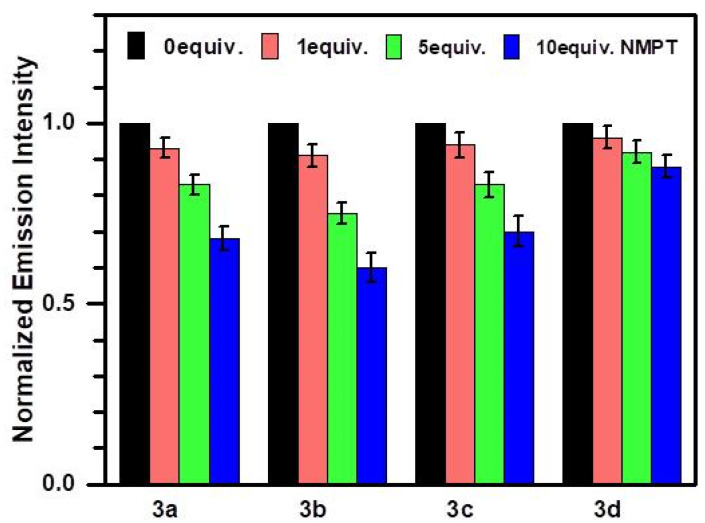
Fluorescence quenching in carbazole-appended calixarenes **3a**–**d** (DCE, 10 μM) upon the addition of N-methylpyridinium triflate (NMPT, **2**).

With these fluorescent calixarenes in hand, we moved to explore their complexation properties toward **2**. As expected, addition of **2** to a 10 µM solution of a calixarene in 1,2-dichloroethane (DCE) resulted in the fluorescence decrease (Figure 4). Titration of the solutions of **3** with 1–10 equiv. of **2** allowed measurements of binding constants (Table 1), which were in the same range reported for calix[4]arene receptors from the UV measurements in chloroform at similar concentrations [10]. The overall numbers (~4000–6000 M^−1^) are higher than *K*_ass_ obtained by the ^1^H NMR technique (*K*_ass_ = 162 ± 13 M^−1^ for **3c**) at higher concentrations. The latter compares well with the literature data for NMP cation complexation obtained by the ^1^H NMR technique for calixarene hosts with a single cavity [9,10]. Importantly, the most significant drop in the fluorescence intensity was observed for compound **3b**, bearing an electron-rich piperidine moiety at the ring C opposite to the fluorophore unit. Calixarene **3a**, unsubstituted at the upper rim, showed a weaker response while **3c**, possessing an electron withdrawing cyano group, was the least responsive among these three compounds. Interestingly, calixarene **3d** showed the weakest response to the presence of the cation **2** despite having an electron-donating substituent at the upper rim (Figure 5, Table 1). These results suggest that the complexation of **2** within the calixarene cavity is directed by the π interactions with the aromatic rings A and C. With the electron-donating piperidine at ring C, the complexation is enhanced, while with the electron-withdrawing CN at ring C, the complexation is weakened compared with the parent **3a**. On the other hand, an electron-donating piperidine unit at ring B weakens cation complexation presumably due to repulsive steric interactions between the piperidine and **2** (Supplementary Materials, Figure S3). Thus, the preset parallel alignment of rings A and C appears more important in the cation complexation within the calixarene cavity over higher electron density in rings B and D, which are prevented from maximizing their π interactions due to hydrogen bonding at the lower rim.

**Figure 6:**
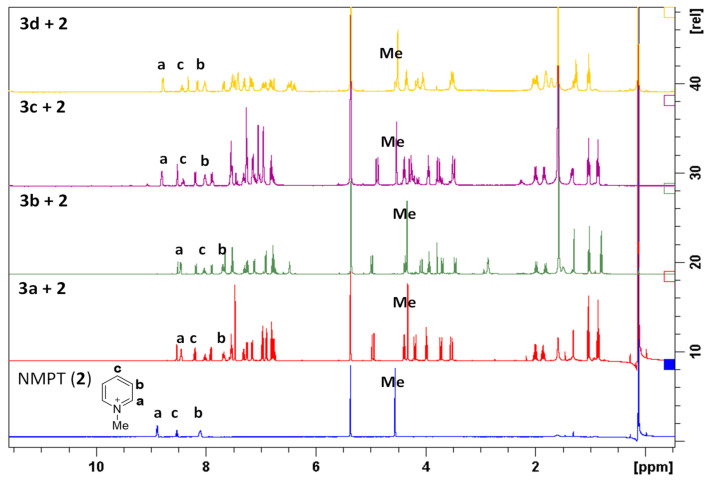
^1^H NMR (400 MHz) host–guest complexation studies in CD_2_Cl_2_ (5 mM).

The fluorescence analysis was further corroborated by the ^1^H NMR studies of complexation of **2** by **3a**–**d**. Unlike the fluorescence quenching which would likely depend on the guest orientation within the cavity, the chemical shifts of the host’s protons should only reflect the strength of the host–guest interactions. At 5 mM concentrations, the 1:1 mixtures of **2** with **3a** or **3b** showed significant upfield shift for the N-CH_3_ group and aromatic protons (Figure 6, Table 2). On the other hand, the same signals were only slightly shifted in the case of **3c** and **3d**, testifying to weaker host–guest interactions. Higher sensitivity of the aromatic protons in **2** suggests that the aromatic ring is likely partly immersed into the calixarene cavity with ensuing π–π interactions [10].

## 3. Materials and Methods

The synthetic manipulations involving air-sensitive compounds were performed in a nitrogen-filled Innovative Technology or Vigor glove box. All solvents were degassed and stored under high-purity nitrogen and activated 4Å molecular sieves. All deuterated solvents were stored under high-purity nitrogen on 3Å molecular sieves. Commercially available reagents (Aldrich, Strem, and Fluka) were used as received. The NMR spectra were recorded on a Bruker Avance 400 MHz spectrometer. ^1^H and ^13^C NMR signals are reported in ppm downfield from TMS. All measurements were performed at 22 °C in CDCl_3_/CD_2_Cl_2_ unless stated otherwise. Mass spectra were recorded on a VG-Autospec M-250 instrument. UV and fluorescence spectra were recorded on a Vernier fluorescence/UV-Vis spectrophotometer and Hitachi F-2710 fluorescence spectrophotometer.

Synthesis of 5: A sample of 4.24 g (10.0 mmol) of calix[4]arene 4 and 0.64 g (11.8 mmol) of NaOCH_3_ was refluxed in 300 mL of CH_3_CN for 30 min to monodeprotonate the calix[4]arene completely. To this, 2.4 mL (4.18 g, 24.6 mmol) of n-propyl iodide was added, and the reaction mixture was further refluxed for 12 h. After the completion of the reaction (monitored by TLC), the reaction mixture was neutralized with a few drops of acetic acid, and the solvent was removed to leave an off-white residue. The residue was dissolved in 150 mL CHCl_3_ and successively washed with H_2_O and brine. The organic phase was separated, dried over anhydrous MgSO_4_, and evaporated. The residue was recrystallized from CHCl_3_ with a slow addition of CH_3_OH to yield the corresponding monoalkylated calix[4]arene 5. Yield: 3.26 g (70%); White solid; ^1^H NMR (CDCl_3_, 400 MHz): δ 10.25 (s, 1H), 9.79 (s, 1H), 9.48 (s, 1H), 7.05–7.13 (m, 6H), 6.93 (t, J = 7.7 Hz, 1H), 6.79 (t, J = 7.0 Hz, 3H), 6.17–6.76 (m, 2H), 4.45 (d, J = 13.4 Hz, 2H), 4.32 (d, J = 13.6 Hz, 2H), 4.18 (t, J = 6.9 Hz, 2H), 3.53 (d, J = 1.7 Hz, 2H), 3.50 (br s, 2H), 2.24 (q, J = 7.3, 14.7 Hz, 2H), 1.34 (t, J = 7.4 Hz, 3H). ^13^C NMR (CDCl_3_, 100 MHz): δ 151.58, 150.95, 149.36, 148.91, 134.39, 129.46, 129.11, 128.94, 128.88, 128.56, 128.37, 126.21, 122.38, 122.09, 121.05, 79.16, 32.05, 31.84, 31.56, 23.42, 10.80. ESI-MS calcd for [M+Na]^+^ C_31_H_30_NaO_4_ 489.20, found 489.46.

Synthesis of 6: To a suspension of 1.81 g (3.9 mmol) of mono-propyl ether 5 and 1,8-bis(dimethylamino)naphthalene (proton sponge) (2.16 g, 10.1 mmol) in dry CH_2_Cl_2_ (40 mL) at 0 °C, trifluoromethanesulphonic anhydride (1.3 mL, 7.8 mmol) was added under nitrogen. After 2 h of stirring at room temperature, the organic layer was washed twice with HCl 10% and once with water, dried over MgSO_4_, and evaporated. The residue was subjected to column chromatography purification (CH_2_Cl_2_/Hexane 3/10 *v*/*v*) giving product 6. Yield: 1.82 g (78%); White solid; ^1^H NMR (CDCl_3_, 400 MHz): δ 7.34 (s, 2H), 7.17 (dd, J = 1.4, 7.5 Hz, 2H), 7.09 (dd, J = 1.5, 7.6 Hz, 2H), 7.05 (s, 1H), 7.02 (s, 1H), 6.88–6.92 (m, 3H), 6.79–6.84 (m, 1H), 6.75 (t, J = 7.6 Hz, 2H), 4.50 (d, J = 14.6 Hz, 2H), 4.19 (t, J = 5.5 Hz, 2H), 4.01 (d, J = 13.8 Hz, 2H), 3.58 (d, J = 13.8 Hz, 2H), 3.46 (d, J = 13.8 Hz, 2H), 2.19 (sextet, J = 7.7, 14.2 Hz, 2H), 1.34 (t, J = 7.5 Hz, 3H). ^13^C NMR (CDCl_3_, 100 MHz): δ 152.96, 149.95, 143.37, 133.99, 132.66, 129.96, 129.65, 129.07, 128.89, 128.63, 127.53, 127.07, 125.68, 119.63, 80.26, 31.95, 31.72, 23.19, 10.56. 19F NMR: −74.34 (s). ESI-MS calcd for [M+Na]^+^ C_32_H_29_F_3_NaO_6_S 621.15, found 621.41.

Synthesis of carbazole-appended calix[4]arene 3a: To a mixture of Pd_2_dba_3_ (0.05 equiv.) and P(t-Bu)_3_H^+^ BF_4_^−^ (0.2 equiv.) dissolved in 10 mL of dry DMF, CuI (2.5 equiv.), DBU (4 equiv.), carbazole alkyne (5 equiv.) and triflate 6 (0.25 mmol) were added and the mixture was heated at 85 °C in an oil bath for 12 h. The solvent was evaporated, and the resulting crude product was dissolved in CH_2_Cl_2_ and washed with brine several times. Drying the CH_2_Cl_2_ extract over MgSO_4_ followed by solvent removal under vacuum gave the crude product. The residue was subjected to column chromatography (CH_2_Cl_2_/Hexane 4/10 *v*/*v*) to obtain the pure compound 3a. Yield: 0.106 g (62%); White solid; ^1^H NMR (CD_2_Cl_2_, 400 MHz): δ 8.53 (d, J = 0.9 Hz, 1H), 8.19 (d, J = 7.8 Hz, 1H), 7.90 (dd, J = 1.6, 8.5 Hz, 1H), 7.52–7.54 (m, 3H), 7.36 (s, 2H), 7.29–7.33 (m, 1H), 7.22 (dd, J = 1.4, 7.5 Hz, 2H), 7.12 (dd, J = 1.5, 7.6 Hz, 2H), 7.02–7.04 (m, 2H), 6.87–6.96 (m, 4H), 6.76 (t, J = 7.5 Hz, 2H), 4.93 (d, J = 12.1 Hz, 2H), 4.39 (t, J = 8.0 Hz, 2H), 4.17 (d, J = 14.7 Hz, 2H), 3.99 (t, J = 5.3 Hz, 2H), 3.69 (d, J = 13.3 Hz, 2H), 3.51 (d, J = 13.3 Hz, 2H), 1.96–2.05 (m, 2H), 1.83–1.92 (m, 2H), 1.04 (t, J = 7.0 Hz, 3H), 0.87 (t, J = 7.9 Hz, 3H). ^13^C NMR (CD_2_Cl_2_, 100 MHz): δ 153.8, 151.5, 141.7, 141.4, 140.4, 133.2, 129.8, 129.7, 129.5, 129.4, 129.1, 128.5, 128.4, 128.0, 127.7, 127.4, 127.2, 126.1, 125.8, 124.1, 123.0, 122.8, 120.8, 119.4, 114.3, 109.1, 108.8, 98.6, 86.5, 78.7, 44.9, 36.6, 31.9, 23.4, 22.5, 11.9, 10.7. HRMS (ESI-TOF) m/z [M+H]^+^ calcd for C_48_H_44_NO_3_ 682.3321, found 682.3323.

Synthesis of 8: The reaction was carried out under an inert atmosphere of pure nitrogen. To a stirred suspension of Pd(OAc)_2_ (0.014 g, 0.063 mmol), P(t-Bu)_3_ (0.019 g, 0.095 mmol) and sodium tert-butoxide (0.121 g, 1.26 mmol), in toluene (15 mL) were added piperidine (0.065 g, 0.75 mmol) and compound 7 (0.343 g, 0.63 mmol). The reaction mixture was then stirred at 85 °C for 48 h. The solvent was evaporated, and the resulting crude product was dissolved in EtOAc (50 mL), washed with water (5 mL × 2), brine, and dried over anhydrous MgSO_4_. Removal of solvent under reduced pressure and column chromatographic purification with EtOAc/Hexane (2:8 *v*/*v*) gave pure compounds 8 in 78% yields (0.270 g). Off-white solid: ^1^H NMR (CD_2_Cl_2_, 400 MHz): δ 9.43 (br s, 2H), 6.99–7.09 (m, 7H), 6.63–6.71 (m, 5H), 4.34 (d, J = 12.6 Hz, 2H), 4.26 (d, J = 12.6 Hz, 2H), 4.08 (t, J = 6.1 Hz, 2H), 3.49 (d, J 12.6 Hz, 2H), 3.39 (d, J = 12.6 Hz, 2H), 3.93–3.96 (m, 4H), 2.16–2.19 (m, 4H), 1.61–1.63 (m 4H), 1.49–1.52 (m, 2H), 1.29 (t, J = 6.8 Hz, 3H). ^13^C NMR (CD_2_Cl_2_, 100 MHz): δ 186.42, 177.34, 160.20, 158.22, 151.17, 150.30, 149.44, 144.46, 134.36, 133.99, 130.69, 129.22, 128.85, 128.76, 128.64, 128.52, 128.36, 128.15, 127.26, 126.01, 124.19, 121.90, 120.86, 120.25, 117.74, 117.41, 117.11, 79.18, 78.60, 78.34, 51.21, 51.04, 32.70, 31.87, 30.70, 30.55, 30.43, 26.16, 24.29, 23.43, 10.68. ESI-MS calcd for [M+H]^+^ C_36_H_40_NO_4_ 550.30, found 550.62.

Synthesis of 9: To a suspension of 8 (0.270 g, 0.49 mmol) and 1,8-bis(dimethylamino)naphthalene (proton sponge) (0.272 g, 1.27 mmol) in dry CH_2_Cl_2_ (20 mL) at 0 °C trifluoromethanesulfonic anhydride (0.276 g, 0.16 mL, 0.98 mmol) was added under nitrogen. After 2 h of stirring at room temperature, the organic layer was washed once with 10% HCl and once with water, dried over anhydrous MgSO_4_, and evaporated. The residue was purified by column chromatography (silica gel, CH_2_Cl_2_/Hexane 4/10 *v*/*v*) to give the title compound as white solid. Yield: 0.244 g (73%); White solid; ^1^H NMR (CD_2_Cl_2_, 400 MHz): δ 7.65 (br s, 2H), 7.19 (dd, J = 1.5, 7.6 Hz, 2H), 7.14 (dd, J = 1.5, 7.6 Hz, 2H), 7.02 (s, 1H), 6.99 (s, 1H), 6.92–6.94 (m, 1H), 6.75 (t, J = 7.5Hz, 2H), 6.62 (s, 2H), 4.50 (d, J = 12.8 Hz, 2H), 4.17 (t, J = 6.4 Hz, 2H), 3.99 (d, J = 12.8 Hz, 2H), 3.55 (d, J = 12.8 Hz, 2H), 3.48 (d, J = 12.8 Hz, 2H), 2.99 (t, J = 5.3 Hz, 2H), 2.15–2.36 (m, 2H), 1.51–1.67 (m, 4H), 1.52–1.56 (m, 2H), 1.34 (t, J = 7.5 Hz, 3H). ^13^C NMR (CD_2_Cl_2_, 100 MHz): δ 153.03, 150.76, 143.28, 142.51, 134.13, 132.94, 130.04, 129.82, 128.88, 128.79, 128.58, 127.44, 125.93, 120.64, 119.70, 117.47, 117.21, 80.43, 50.44, 32.11, 31.86, 26.02, 24.18, 23.17, 10.36. 19F NMR: −74.74 (s). ESI-MS calcd for [M+H]^+^ C_37_H_39_F_3_NO_6_S 682.25, found 682.47.

Synthesis of carbazole-appended calix[4]arene 3b: To a mixture of Pd_2_dba_3_ (0.05 equiv.) and P(t-Bu)_3_H^+^ BF_4_^-^ (0.2 equiv.) dissolved in 10 mL of dry DMF, CuI (2.5 equiv.), DBU (4 equiv.), carbazole alkyne (5 equiv.) and triflate 9 (0.25 mmol) were added and the mixture was heated at 85 °C in an oil bath for 12 h. The solvent was evaporated, and the resulting crude product was dissolved in CH_2_Cl_2_ and washed with brine several times. Drying the CH_2_Cl_2_ extract over anhydrous MgSO_4_ followed by solvent removal under vacuum gave the crude product. The residue was subjected to column chromatography (CH_2_Cl_2_/Hexane 4/10 *v*/*v*) to obtain the pure compound. Yield: 0.090 g (47%); Slightly yellow solid; ^1^H NMR (CD_2_Cl_2_, 400 MHz): δ 8.52 (d, J = 1.0 Hz, 1H), 8.20 (dd, J = 4.8, 3.9 Hz, 1H), 7.91 (dd, J = 8.5, 1.6 Hz, 1H), 7.62–7.49 (m, 5H), 7.28–7.34 (m, 1H), 7.22 (dd, J = 7.5, 1.5 Hz, 2H), 7.09 (dd, J = 7.6, 1.6 Hz, 2H), 7.02–6.90 (m, 3H), 6.71 (t, J = 7.5 Hz, 2H), 6.58 (s, 2H), 4.98 (d, J = 12.8 Hz, 2H), 4.39 (t, J = 7.1 Hz, 2H), 4.08 (d, J = 13.5 Hz, 2H), 3.96 (t, J = 6.6 Hz, 2H), 3.69 (d, J = 12.8 Hz, 2H), 3.46 (d, J = 12.9 Hz, 2H), 3.00–2.95 (m, 4H), 2.06–1.95 (m, 2H), 1.90–1.77 (m, 2H), 1.70–1.59 (m, 4H), 1.52–1.54 (m, 2H), 1.05 (t, J = 7.5 Hz, 3H), 0.82 (t, J = 7.4 Hz, 3H). ^13^C NMR (CD_2_Cl_2_, 100 MHz): δ 153.64, 150.28, 143.67, 141.78, 141.10, 140.32, 133.32, 129.53, 129.03, 128.73, 128.51, 128.20, 127.83, 127.23, 127.09, 126.81, 126.09, 123.72, 123.32, 122.90, 122.65, 120.62, 120.18, 119.52, 119.31, 117.28, 114.75, 109.20, 108.97, 97.33, 87.30, 79.50, 50.70, 44.87, 36.33, 32.17, 26.14, 24.26, 23.33, 22.46, 11.64, 10.35. HRMS (ESI-TOF) m/z [M+H]^+^ calcd for C_53_H_53_N_2_O_3_ 765.4056, found 765.4055.

Synthesis of 10: To a solution of 7 (1.0 g, 1.83 mmol) in DMF (50 mL), CuCN (0.492 g, 5.49 mmol) was added. The resulting heterogeneous mixture was poured into a thick wall glass pressure round bottom flask and then heated at 180 °C for 48 h under vigorous stirring. After cooling, the solvent was completely evaporated under reduced pressure. The resulting sticky residue was extracted twice with hot ethyl acetate (2 × 100 mL). The combined organic phases were then washed twice with brine (2 × 100 mL), dried over anhydrous MgSO_4_, and then evaporated to dryness (the separated water phase was carefully treated with a solution of sodium hypochlorite to destroy the residuals cyanide ions). Purification of the solid residue by silica column chromatography (CH_2_Cl_2_/hexane, *v*/*v* 8:2) gave title compound. Yield: 0.603 g (67%); Slightly yellow solid; ^1^H NMR (CDCl_3_, 400 MHz): δ 9.43 (s, 1H), 9.13 (s, 2H), 7.41 (s, 2H), 7.14–7.08 (m, 4H), 7.04 (d, J = 7.6 Hz, 2H), 6.75 (m, 3H), 4.43 (d, J = 13.1 Hz, 2H), 4.29 (d, J = 13.8 Hz, 2H), 4.18 (dt, J = 14.3, 7.0 Hz, 2H), 3.54 (d, J = 3.7 Hz, 2H), 3.51 (d, J = 3.0 Hz, 2H), 2.30–2.18 (m, 2H), 0.93 (t, J = 6.9 Hz, 3H). ^13^C NMR (CDCl_3_, 100 MHz): δ 157.12, 153.92, 152.75, 143.34, 134.69, 133.88, 133.83, 132.95, 132.80, 132.69, 132.46, 130.99, 130.72, 130.29, 129.83, 129.48, 129.09, 128.73, 127.91, 127.69, 127.39, 126.82, 124.75, 120.56, 120.26, 119.84, 118.09, 117.38, 110.75, 80.54, 31.94, 31.45, 23.22, 23.22, 10.38. ESI-MS calcd for [M−H]^−^ C_32_H_28_NO_4_ 490.20, found 490.48.

Synthesis of 11: To a suspension of 0.500 g (1.02 mmol) of mono-propyl ether 10 and 1,8-bis(dimethylamino)naphthalene (proton sponge) (0.567 g, 2.65 mmol) in dry CH_2_Cl_2_ (20 mL) at 0 °C, trifluoromethanesulphonic anhydride (0.34 mL, 2.04 mmol) was added under nitrogen. After 2 h of stirring at room temperature, the organic layer was washed twice with 10% HCl and once with water, dried over anhydrous MgSO4, and evaporated. The residue was subjected to column chromatography purification (CH_2_Cl_2_/Hexane 3/10 *v*/*v*) giving product 11. Yield: 0.439 g (69%); Off-white solid; 1H NMR (CD_2_Cl_2_, 400 MHz): δ 7.33 (s, 2H), 7.23 (dd, J = 7.5, 1.3 Hz, 2H), 7.11 (dd, J = 7.6, 1.5 Hz, 2H), 6.96–6.84 (m, 3H), 6.81 (t, J = 7.5 Hz, 2H), 6.73 (s, 2H), 4.49 (d, J = 13.5 Hz, 2H), 4.18 (t, J = 6.5 Hz, 2H), 4.08 (d, J = 14.1 Hz, 2H), 3.61 (d, J = 14.1 Hz, 2H), 3.52 (d, J = 13.5 Hz, 2H), 2.38–2.12 (m, 2H), 1.34 (t, J = 7.4 Hz, 3H). 19F NMR: −74.21 (s). ESI-MS calcd for [M+Na]^+^ C_33_H_28_F_3_NNaO_6_S 646.15, found 646.49.

Synthesis of carbazole-appended calix[4]arene 3c: To a mixture of Pd2dba3 (0.05 equiv.) and P(t-Bu)_3_H^+^BF_4_^−^ (0.2 equiv.) dissolved in 10 mL of dry DMF, CuI (2.5 equiv.), DBU (4 equiv.), carbazole alkyne (5 equiv.) and triflate 11 (0.25 mmol) were added and the mixture was heated at 85 °C in an oil bath for 12 h. The solvent was evaporated, and the resulting crude product was dissolved in CH_2_Cl_2_ and washed with brine several times. Drying the CH_2_Cl_2_ extract over anhydrous MgSO_4_ followed by solvent removal under vacuum gave the crude product. The residue was subjected to column chromatography (CH_2_Cl_2_/Hexane 4/10 *v*/*v*) to obtain the pure compound. Yield: 0.053 g (30%); Off-white solid; ^1^H NMR (CD_2_Cl_2_, 400 MHz): δ 8.53 (s, 1H), 8.20 (d, J = 7.7 Hz, 1H), 7.89 (d, J = 7.5 Hz, 1H), 7.54 (t, J = 7.0 Hz, 2H), 7.35–7.23 (m, 5H), 7.13 (d, J = 7.9 Hz, 2H), 7.04 (s, 2H), 6.97 (s, 3H), 6.85–6.65 (m, 3H), 4.89 (d, J = 13.4 Hz, 2H), 4.39 (t, J = 7.0 Hz, 2H), 4.29 (d, J = 13.5 Hz, 2H), 3.95 (t, J = 6.3 Hz, 2H), 3.77 (d, J = 13.5 Hz, 2H), 3.49 (d, J = 13.5 Hz, 2H), 1.98 (m, 2H), 1.85 (m, 2H), 1.04 (t, J = 7.3 Hz, 3H), 0.87 (t, J = 7.4 Hz, 3H). ^13^C NMR (CD2Cl2, 100 MHz): δ 155.88, 153.60, 141.62, 141.08, 140.60, 135.31, 133.72, 133.48, 133.22, 130.78, 129.47, 129.42, 129.30, 128.84, 128.56, 128.24, 128.06, 127.93, 127.32, 126.63, 126.23, 126.13, 123.92, 122.47, 122.07, 120.61, 120.56, 119.79, 119.43, 118.52, 113.23, 109.24, 109.06, 99.82, 85.68, 80.50, 78.92, 46.66, 44.82, 36.38, 31.27, 23.39, 22.38, 11.56, 10.29. HRMS (ESI-TOF) m/z [M+H]^+^ calcd for C_49_H_43_N_2_O_3_ 707.3274, found 707.3279.

Synthesis of 13: Calixarene 12 [24] (2.00 g, 2.87 mmol) was suspended in CH_3_CN (40 mL), and after addition of 48% HBr (10 mL) the mixture was stirred at 60 °C for 12 h. The resulting suspension was diluted with CH_2_Cl_2_ and washed twice with water and once with brine. The organic layer dried over MgSO_4_ and evaporated. The residue was washed several times with MeOH to remove benzyl alcohol giving the free phenol. The residue was recrystallized from CH_3_Cl/MeOH to give pure calixarene 13. Yield: 1.04 g (70%); Pale yellow solid; ^1^H NMR (CDCl_3_, 400 MHz): δ 9.76 (d, J = 1.0 Hz, 1H), 9.58 (d, J = 1.0 Hz, 1H), 9.38 (d, J = 1.1 Hz, 1H), 7.21 (d, J = 1.0 Hz, 2H), 7.16–7.03 (m, 5H), 7.01–6.92 (m, 2H), 6.80–6.70 (m, 2H), 4.48 (d, J = 12.8 Hz, 1H), 4.36–4.21 (m, 3H), 4.20–4.05 (m, 2H), 3.63–3.25 (m, 4H), 2.31–2.13 (m, 2H), 1.32 (td, J = 7.3, 1.3 Hz, 3H). ^13^C NMR (CDCl_3_, 100 MHz): δ 150.56, 150.51, 150.43, 149.38, 134.09, 134.06, 131.20, 131.04, 130.88, 130.69, 129.73, 129.16, 128.90, 128.72, 128.43, 128.37, 128.02, 126.36, 125.59, 125.49, 122.17, 121.47, 119.16, 112.28, 79.24, 31.94, 31.89, 31.86, 3.31, 31.04, 23.38, 10.77. ESI-MS calcd for [M-H]^−^ C_31_H_28_BrO_4_ 543.12, found 543.50.

Synthesis of 14: To a suspension of 1.00 g (1.93 mmol) of mono-propyl ether 13 and 1,8-bis(dimethylamino)naphthalene (proton sponge) (1.08 g, 5.02 mmol) in dry CH_2_Cl_2_ (40 mL) at 0 °C, trifluoromethanesulphonic anhydride (0.65 mL, 3.86 mmol) was added under nitrogen. After 2 h of stirring at room temperature, the organic layer was washed twice with 10% HCl and once with water, dried over anhydrous MgSO4, and evaporated. The residue was subjected to column chromatography purification (CH_2_Cl_2_/Hexane 3/10 *v*/*v*) giving product 14.

Yield: 0.938 g (75%); White solid; ^1^H NMR (CDCl_3_, 400 MHz): δ 7.43 (s, 1H), 7.30 (s, 2H), 7.27–7.10 (m, 4H), 6.99 (dd, J = 13.1, 7.5 Hz, 2H), 6.91–6.73 (m, 4H), 4.48 (dd, J = 12.6, 9.5 Hz, 2H), 4.17 (t, J = 6.2 Hz, 2H), 3.99 (t, J = 13.0 Hz, 2H), 3.69–3.37 (m, 4H), 2.29–2.06 (m, 2H), 0.92 (t, J = 6.0 Hz, 3H). ^13^C NMR (CDCl_3_, 100 MHz): δ 152.90, 152.18, 149.91, 134.13, 133.11, 132.69, 131.75, 131.28, 131.13, 130.92, 130.32, 130.00, 129.95, 129.63, 128.96, 128.72, 127.62, 127.18, 125.52, 119.72, 111.09, 80.34, 31.93, 31.72, 31.49, 23.18, 22.78, 14.24, 10.53. 19F NMR: −74.34 (s). ESI-MS calcd for [M+Na]^+^ C_32_H_28_BrF_3_O_6_SNa 699.06, found 699.52.

Synthesis of 15: To a mixture of Pd_2_dba_3_ (0.05 equiv.) and P(t-Bu)_3_H^+^ BF_4_^−^ (0.2 equiv.) dissolved in 10 mL of dry DMF, CuI (2.5 equiv.), DBU (4 equiv.), carbazole alkyne (5 equiv.) and triflate 14 (0.25 mmol) were added and the mixture was heated at 85 °C in an oil bath for 12 h. The solvent was evaporated, and the resulting crude product was dissolved in CH_2_Cl_2_and washed with brine several times. Drying the CH_2_Cl_2_ extract over anhydrous MgSO_4_ followed by solvent removal under vacuum gave the crude product. The residue was subjected to column chromatography (CH_2_Cl_2_/Hexane 4/10 *v*/*v*) to obtain the pure compound. Yield: 0.086 g (45%); Yellow solid; ^1^H NMR (CDCl_3_, 400 MHz): δ 8.47 (s, 1H), 8.18 (d, J = 7.8 Hz, 1H), 7.85 (d, J = 8.4 Hz, 1H), 7.64–7.42 (m, 2H), 7.34 (t, J = 9.3 Hz, 1H), 7.26–7.08 (m, 6H), 7.07–6.92 (m, 3H), 6.80 (m, 5H), 4.90 (t, J = 14.0 Hz, 2H), 4.35 (dd, J = 13.6, 6.6 Hz, 2H), 4.26–3.98 (m, 2H), 3.93 (t, J = 6.4 Hz, 2H), 3.66 (dd, J = 23.6, 11.9 Hz, 2H), 3.43 (dd, J = 25.8, 12.1 Hz, 2H), 1.99 (dd, J = 14.5, 7.2 Hz, 2H), 1.82 (dd, J = 14.0, 7.0 Hz, 2H), 1.03 (t, J = 7.5 Hz, 3H), 0.86 (t, J = 7.3 Hz, 3H). ^13^C NMR (CDCl_3_, 100 MHz): δ 153.03, 151.69, 142.00, 141.07, 140.80, 140.46, 133.37, 132.40, 131.51, 131.47, 131.04, 130.97, 130.74, 130.35, 130.23, 129.86, 129.78, 129.68, 129.51, 129.40, 129.29, 129.13, 128.49, 128.28, 128.12, 127.84, 127.60, 127.46, 127.41, 127.25, 126.48, 126.15, 125.84, 124.09, 120.82, 120.50, 119.45, 114.08, 110.94, 109.11, 108.83, 99.00, 86.22, 80.37, 78.66, 44.92, 37.58, 37.35, 36.64, 36.55, 31.95, 31.85, 23.42, 23.36, 22.50, 11.94, 10.64. ESI-MS calcd for [M-H]^−^ C_48_H_41_BrNO_3_ 758.23, found 758.50.

Synthesis of carbazole-appended calix[4]arene 3d: An oven-dried Schlenk tube was charged with Pd_2_(dba)_3_ (0.05 equiv.), JohnPhos (0.1 equiv.), calix halide 15 (0.08 mmol), amine (0.16 mmol) and toluene (2 mL). The reaction was stirred for few minutes and then LiHMDS (0.9–1.1 M in Hexanes) (0.18 mL) was added via syringe. The reaction vessel was sealed and heated at 80 °C with stirring for 48 h. The reaction mixture was then allowed to cool to room temperature, adsorbed on silica, and purified by column chromatography with EtOAc/hexane (2/8 *v*/*v*). Yield: 0.013 g (20%); Off-white solid; ^1^H NMR (CD_2_Cl_2_, 400 MHz): δ 8.33 (s, 1H), 8.16 (d, J = 7.8 Hz, 1H), 7.68 (d, J = 8.4 Hz, 1H), 7.58–7.45 (m, 3H), 7.42 (d, J = 6.0 Hz, 2H), 7.30 (t, J = 7.2 Hz, 1H), 7.23–7.13 (m, 2H), 7.10 (s, 1H), 7.02–6.76 (m, 4H), 6.66 (s, 1H), 6.56–6.37 (m, 3H), 4.53 (dt, J = 12.1, 5.9 Hz, 2H), 4.35 (t, J = 7.1 Hz, 2H), 4.15 (d, J = 14.1 Hz, 2H), 4.06 (t, J = 6.0 Hz, 2H), 3.58–3.43 (m, 4H), 3.39–2.83 (m, 4H), 2.09–1.93 (m, 4H), 1.81 (s, 4H), 1.72 (s, 2H), 1.26 (t, J = 7.2 Hz, 3H), 1.03 (t, J = 7.3 Hz, 3H). ^13^C NMR (CD_2_Cl_2_, 100 MHz): δ 153.76, 153.37, 151.62, 149.13, 141.05, 140.08, 138.63, 138.11, 133.07, 132.51, 131.60, 131.09, 130.75, 130.60, 129.40, 129.27, 129.18, 129.06, 128.41, 128.31, 127.67, 127.43, 126.15, 125.56, 125.44, 123.56, 123.48, 122.87, 122.41, 120.47, 119.29, 119.03, 114.01, 113.83, 109.15, 109.04, 88.59, 88.02, 78.62, 44.82, 33.93, 33.75, 31.44, 31.24, 27.71, 25.09, 23.35, 22.38, 13.81, 11.57, 10.43. HRMS (ESI-TOF) m/z [M]^+^ calcd for C_53_H_53_N_2_O_3_ 765.4056, found 765.4057.

## 4. Conclusions

In summary, we presented the first studies of the electronic effects on the host–guest complexation in fluorescent calixarene scaffolds. We found that the introduction of an electron-donating substituent in the aromatic ring opposing the fluorophore-substituted ring enhances the complexation of the cationic N-methyl pyridinium guest, while an electron-withdrawing substituent in the same position decreases this complexation. In contrast, an electronic donor at the ring adjacent to the fluorophore-substituted one does not provide stronger binding of the cationic guest. Thus, our results provide strong evidence for the planar cationic guest undergoing π interactions with only one pair of the calixarene aromatic rings which is not involved in the hydrogen bonding at the lower rim. Although more electron rich, this hydrogen bonding between the unsubstituted phenolic rings is likely too strong to make them available for the π interactions.

## Figures and Tables

**Table 1:** Emission intensity dependence on the concentration of **2** [a]. % Decrease in Emission Intensity.

Calixarene	1 Equiv. 2	5 Equiv. 2	10 Equiv. 2	*K*_ass_ [b]
**3a**	7%	17%	33%	4392 ± 150 M^−1^
**3b**	9%	25%	39%	5935 ± 210 M^−1^
**3c [c]**	6%	17%	30%	3900 ± 134 M^−1^
**3d**	4%	8%	12%	1182 ± 76 M^−1^

[a] Solutions of calixarenes **3a**–**d** in DCE (10 μM) were treated with increasing concentrations of NMPT, **2** (0 to 10 equivalents) at 24 °C; [b] the binding constants were calculated directly from the Stern–Volmer plots, see Supplementary Materials for details (page S43); [c] binding constant calculated via NMR measurements was found to be 162 ± 13 M^−1^, see Supplementary Materials for details (page S45).

**Table 2:** Comparative studies of the ^1^H NMR chemical shifts (δ, ppm) of **2** within the calixarene cavities (CD_2_Cl_2_, 5 mM).

Resonance Signal	2	3a + 2	3b + 2	3c + 2	3d + 2
Me	4.56	4.33	4.36	4.53	4.51
H_a_	8.88	8.45	8.47	8.80	8.78
H_b_	8.11	7.68	7.71	8.02	8.02
H_c_	8.54	8.02	8.05	8.42	8.44

## Data Availability

All experimental data are provided in the Supplementary information.

## Supplementary Materials

Synthesis and characterization of all new compounds, UV-vis, fluorescence and NMR spectra, fluorescence and NMR complexation studies. This information can be downloaded at: https://www.mdpi.com/article/10.3390/molecules27175689/s1. Reference [28] has been cited the Supplementary Materials.

## Sample Availability

Samples of the compounds are not available from the authors.
