# Supplementary material for: Electronic Tuning of Host-Guest Interactions within the Cavities of Fluorophore-Appended Calix[4]arenes
Source: Molecules. 2022 Sep 3;27(17):5689. doi: 10.3390/molecules27175689 (PMC9457996; doi:10.3390/molecules27175689)
Supplement: Supplementary file 1 [file molecules-27-05689-s001.zip › molecules-1872318-supplementary.pdf]

Article

Electronic Tuning of Host-Guest Interactions within the Cavities of Fluorophore-Appended Calix[4]arenes

Varun Rawat and Arkadi Vigalok *

School of Chemistry, The Raymond and Beverly Sackler Faculty of Exact Sciences, Tel Aviv University, Tel Aviv 69978, Israel; varunrawat.chemistry@gmail.com
* Correspondence: avigal@tauex.tau.ac.il

Electronic Supporting Information

Contents	Page
1. Experimental Section	S2-S3
2. NMR and Mass Spectra	S4-S40
3. Absorption and Emissions Spectra	S41
4. Host-guest Complexation Studies	S42-S44
5. Binding constant from NMR titration	S45-S46
6. Calculation of Quantum Yield	S47

1. Experimental section

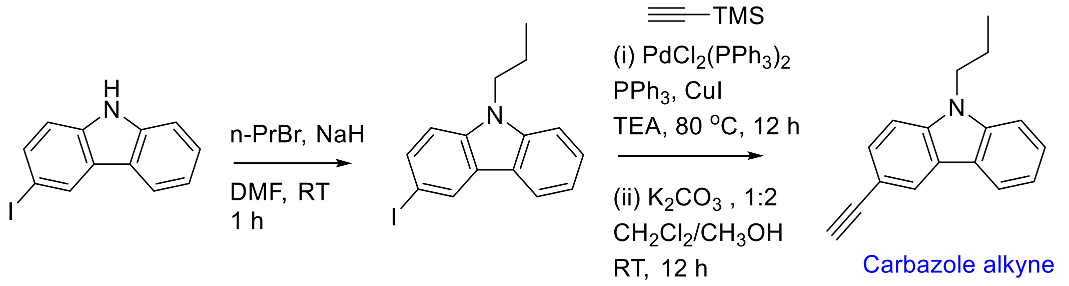

Scheme S1: Synthesis of carbazole alkyne

Synthesis of 9-propyl-3-iodocarbazole

To a stirred solution of 3-iodocarbazole (2.93 g, 10 mmol) in dry DMF (30 mL), NaH (60 wt % in mineral oil; 0.36 g, 15 mmol) and *n*-C_3H_7Br (1.85 g, 1.37 mL, 15 mmol) were added sequentially. The mixture was stirred at room temperature until the reaction was complete, as monitored by TLC. The mixture was quenched with water (2 mL) and most of the solvent evaporated. The crude mixture was dissolved in dichloromethane (CH_2Cl_2) and washed with water, brine and dried over anhydrous $MgSO_4$. Filtration and evaporation of organic solvent followed by purification by column chromatography (silica gel, CH_2Cl_2/petroleum ether, 1/1, v/v), gave the title compound as white solid.

Yield: 3.15 g (94%); White solid; **^{1}H NMR** ($CDCl_3$, 400 MHz): δ 8.40 (d, J = 1.8 Hz, 1H), 8.03 (d, J = 7.4 Hz, 1H), 7.69 (dd, J = 1.9, 8.6 Hz, 1H), 7.47-7.49 (m, 1H), 7.39-7.41 (m, 1H), 7.19-7.27 (m, 2H), 4.4 (t, J = 7.0 Hz, 2H), 1.85-1.95 (m, 2H), 0.96 (t, J = 7.4 Hz, 3H). ^{13}C NMR (101 MHz, $CDCl_3$): δ 140.60, 139.77, 133.88, 129.30, 126.46, 125.47, 121.68, 120.64, 119.42, 110.93, 109.06, 81.33, 44.78, 22.39, 11.93. **ESI-MS** calcd for $[M]^+$ $C_{15}H_{14}IN$ 335.02, found 335.22.

Synthesis of 3-ethynyl-9-propyl-9H-carbazole

A mixture of 3-iodo-9-propyl-9H-carbazole (2.10 g, 6.27 mmol), $Pd(PPh_3)_2Cl_2$ (126 mg, 0.18 mmol), PPh_3 (78.6 mg, 0.3 mmol), CuI (57 mg, 0.3 mmol) in triethylamine (50 mL) was stirred for 5 minutes under inert atmosphere. To this (trimethylsilyl)-acetylene (2.4 mL, 23.5 mmol) was added dropwise and the reaction stirred for additional 4 h at 80 ^{o}C. After the completion of reaction the volatiles were removed, and the residue was dissolved in diethylether and passed through a short silica gel column. The eluent obtained containing the intermediate, 3-(2-(trimethylsilyl)- ethynyl)-9-propyl-9H-carbazole, was concentrated to dryness and dissolved in dichloromethane/methanol mixture (1:2). It was treated with sodium hydroxide, 0.40 g (10 mmol) with overnight stirring. The reaction was quenched by the addition of water, and the organic product was extracted into dichloromethane. The organic layer was dried over anhydrous $MgSO_4$ and evaporated to yield the crude product. It was further purified by column chromatography using hexane/dichloromethane mixture (4:1) as eluent.

Yield: 1.08 g (74%); Yellow liquid; **^{1}H NMR** (CD_2Cl_2, 400 MHz): δ 8.29 (d, J = 1.5 Hz, 1 H), 8.12 (d, J = 7.7 Hz, 1 H), 7.62 (dd, J = 1.5, 8.5 Hz, 1 H), 7.43-7.56 (m, 3 H), 7.28-7.31 (m, 1H), 4.33 (t, J = 7.3 Hz, 2 H), 3.16 (s, 1 H), 1.93-1.98 (m, 2 H), 1.01 (t, J = 8.3 Hz, 3 H). **^{13}C NMR** (CD_2Cl_2, 100 MHz): δ 141.12, 129.60, 126.3, 124.6, 122.8, 120.5, 119.5, 111.9, 109.3, 109.0, 85.0, 75.1, 44.9, 22.4, 11.6. **ESI-MS** calcd for $[M]^+$ $C_{17}H_{15}N$ 233.12, found 233.23.

3. NMR and Mass Spectra

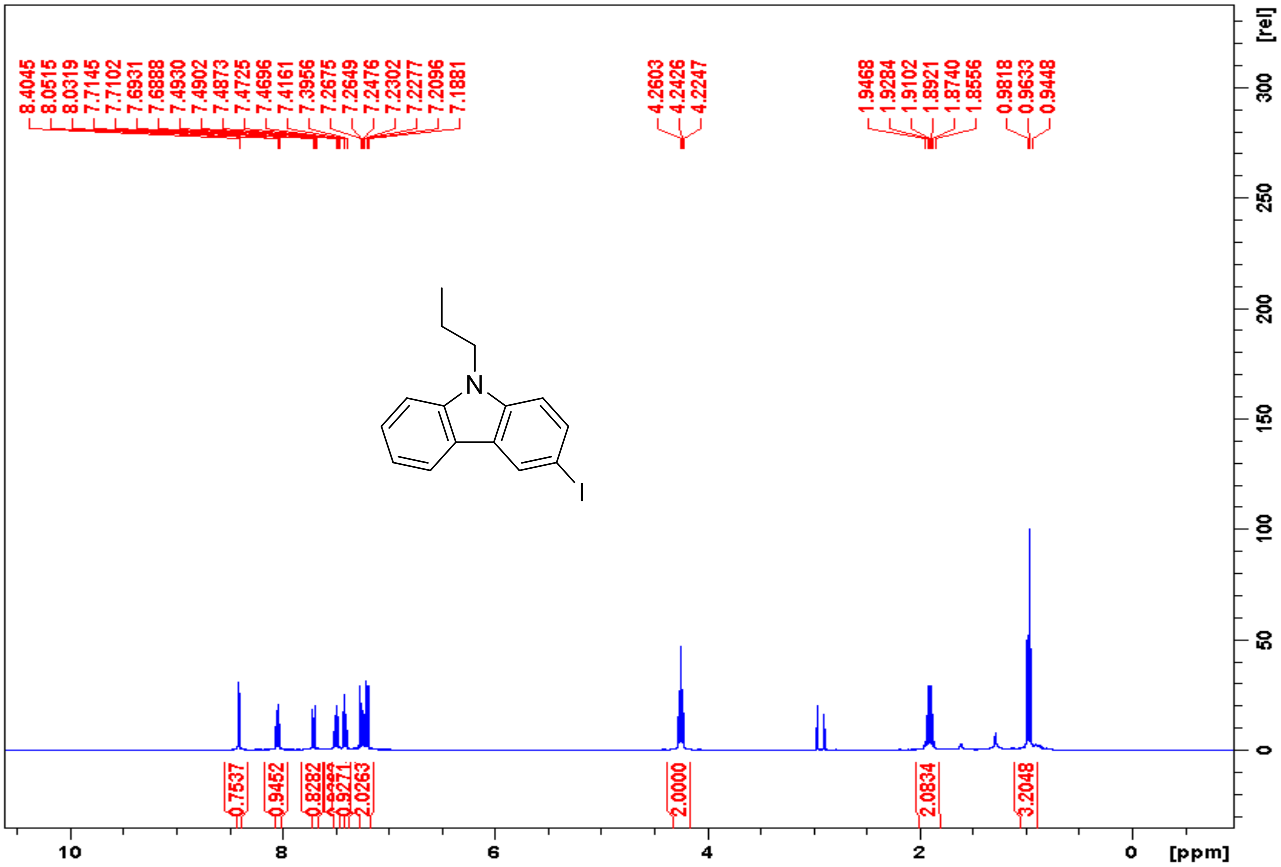

^{1}H NMR of 9-propyl-3-iodocarbazole

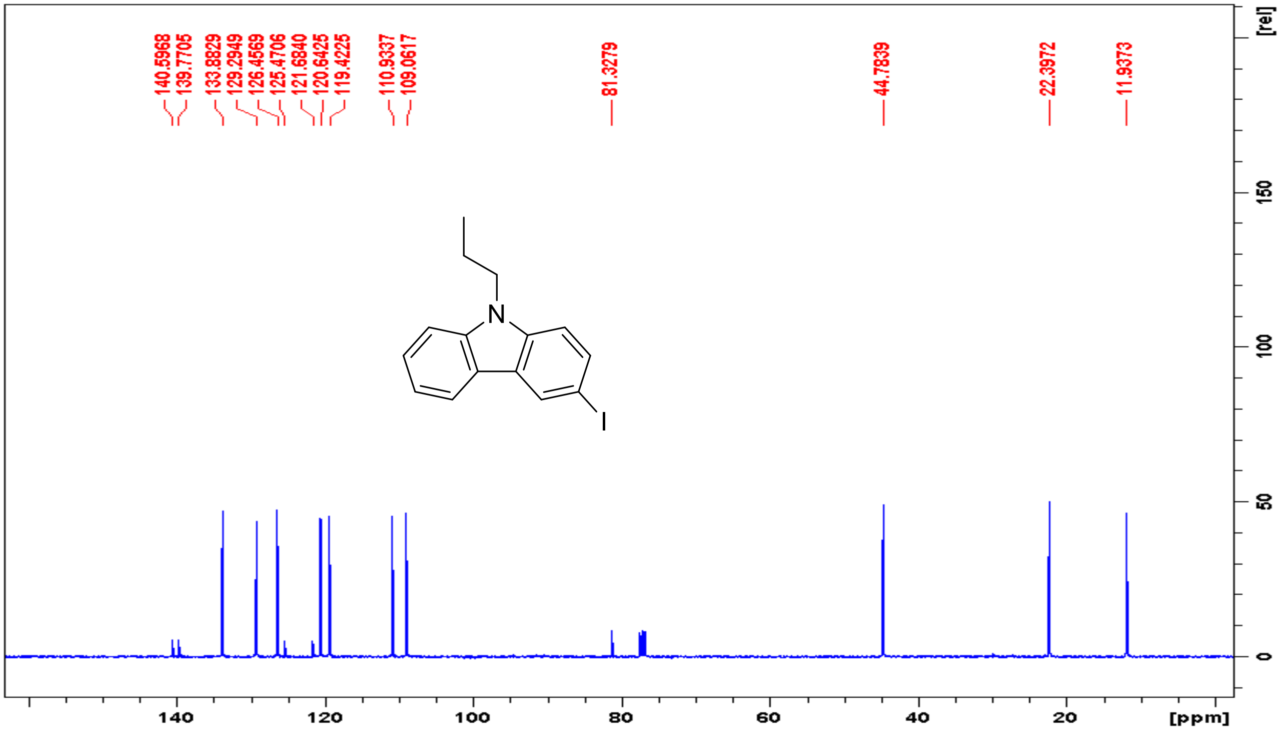

^{13}C NMR of 9-propyl-3-iodocarbazole

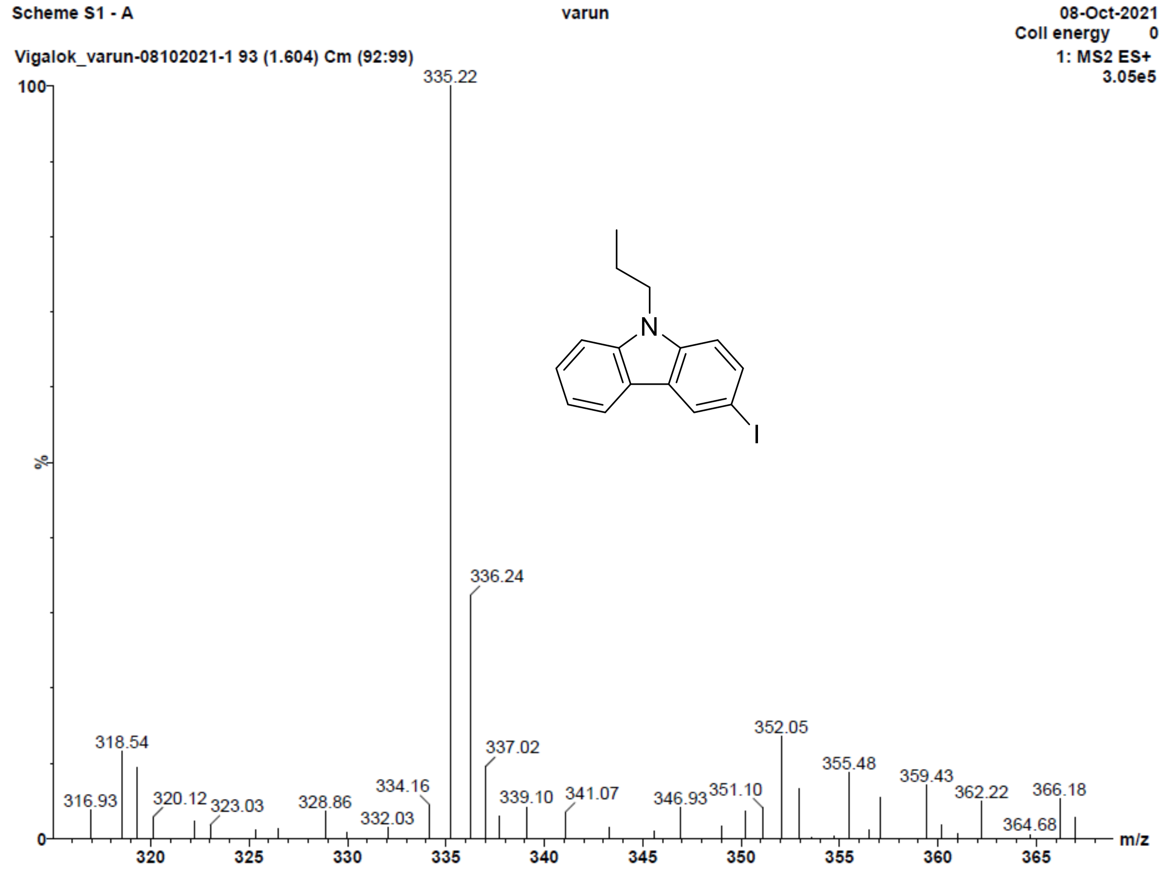

ESI MS of 9-propyl-3-iodocarbazole

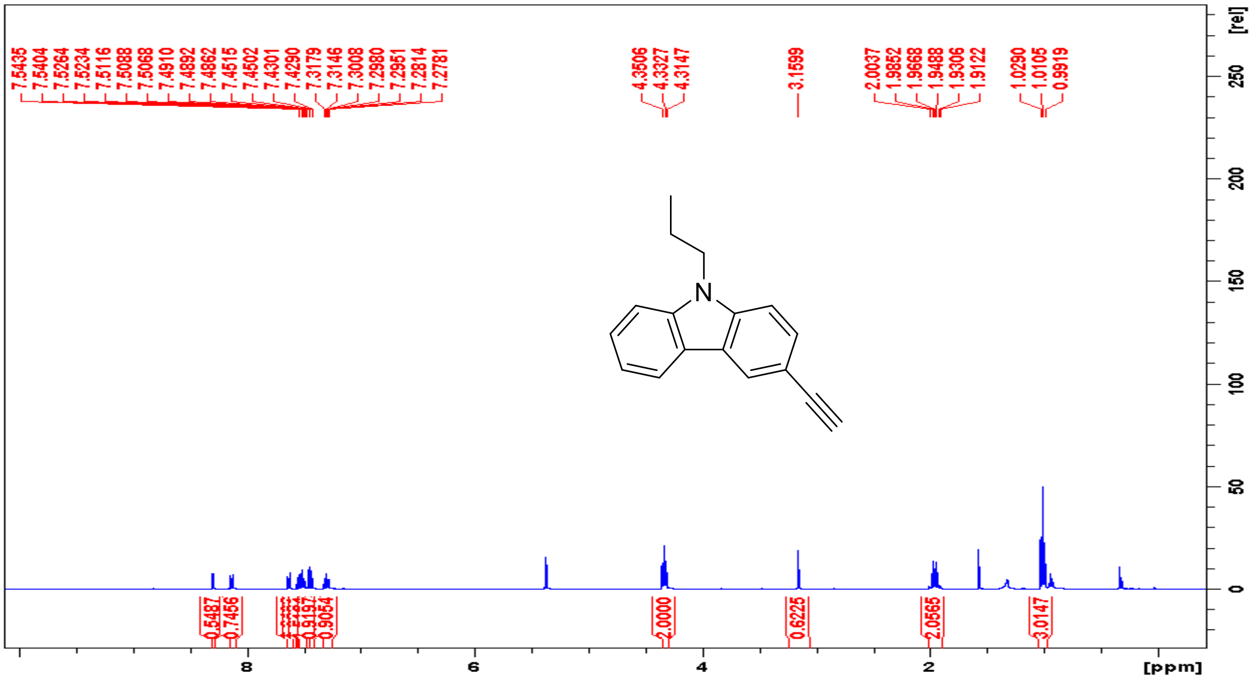

^{1}H NMR of 3-ethynyl-9-propyl-9H-carbazole

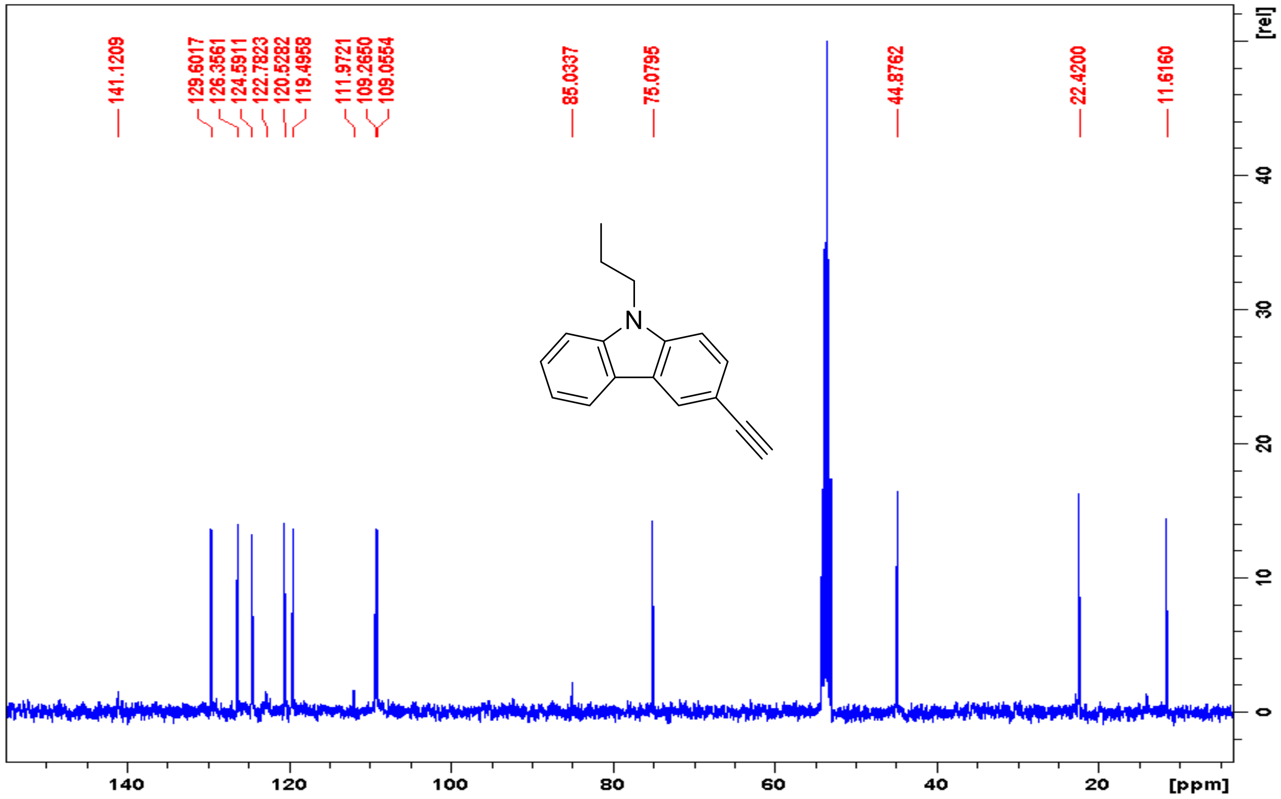

^{13}C NMR of 3-ethynyl-9-propyl-9H-carbazole

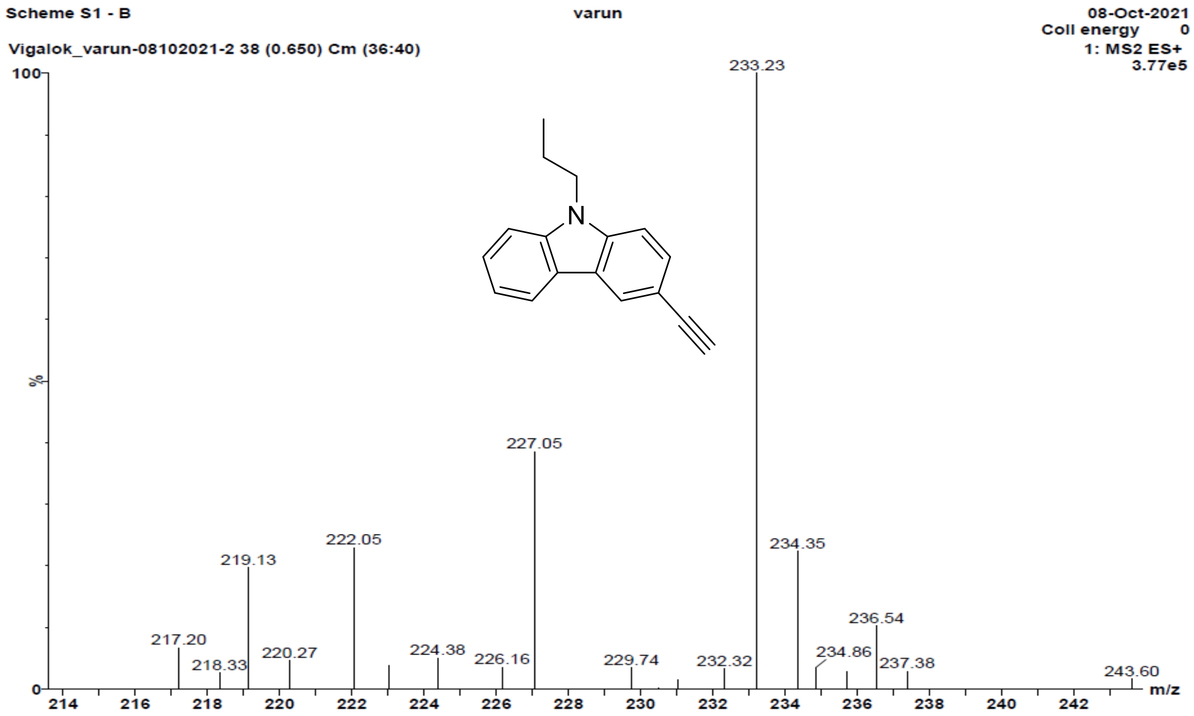

ESI MS of 3-ethynyl-9-propyl-9H-carbazole

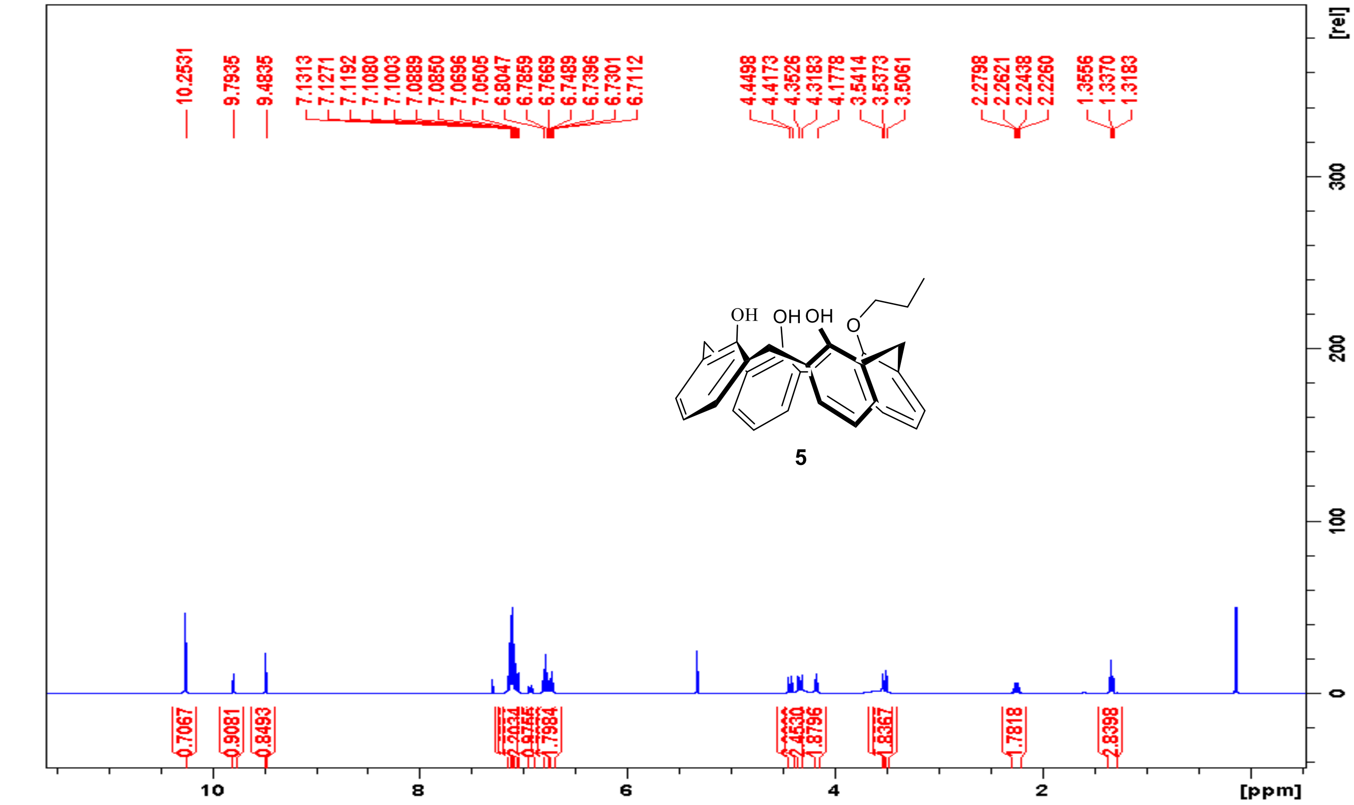

^{1}H NMR spectra of 5

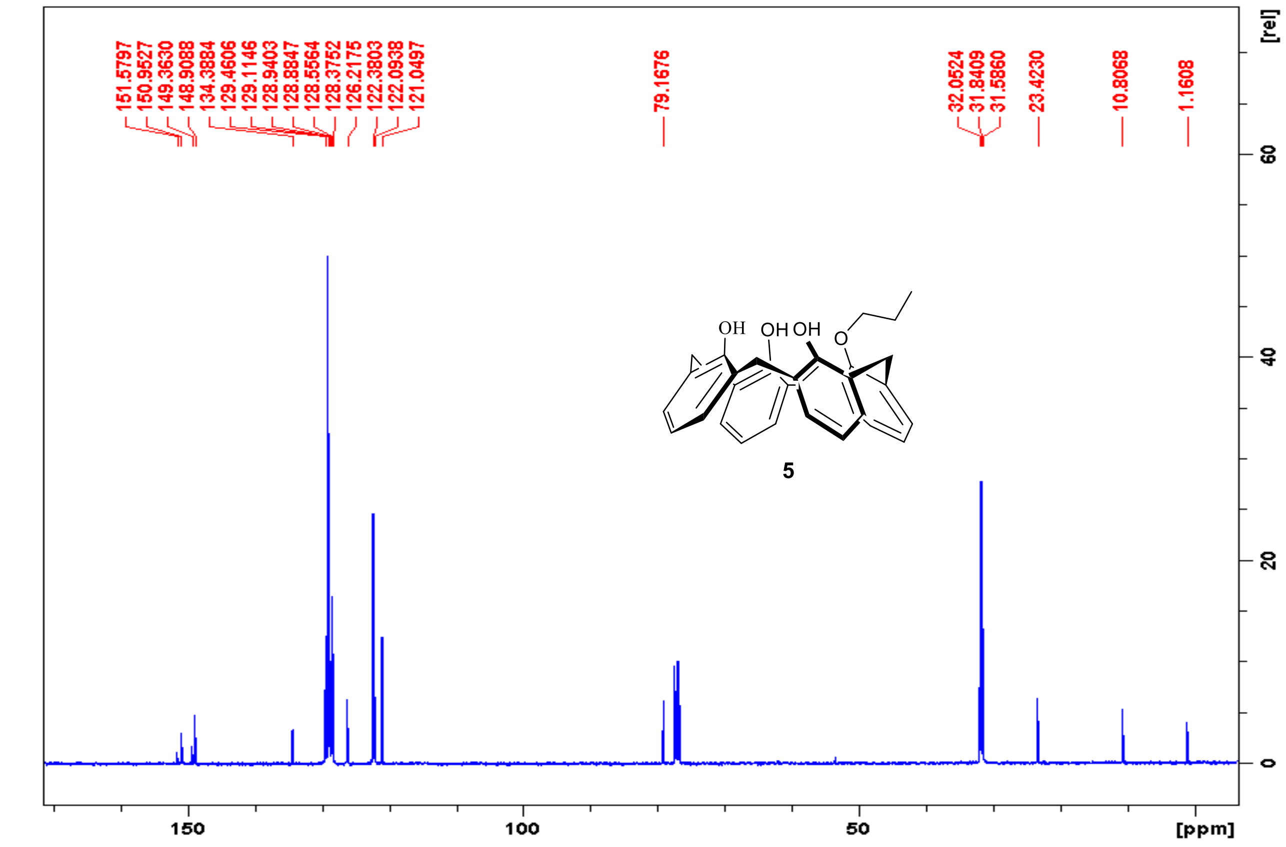

^{13}C NMR spectra of 5

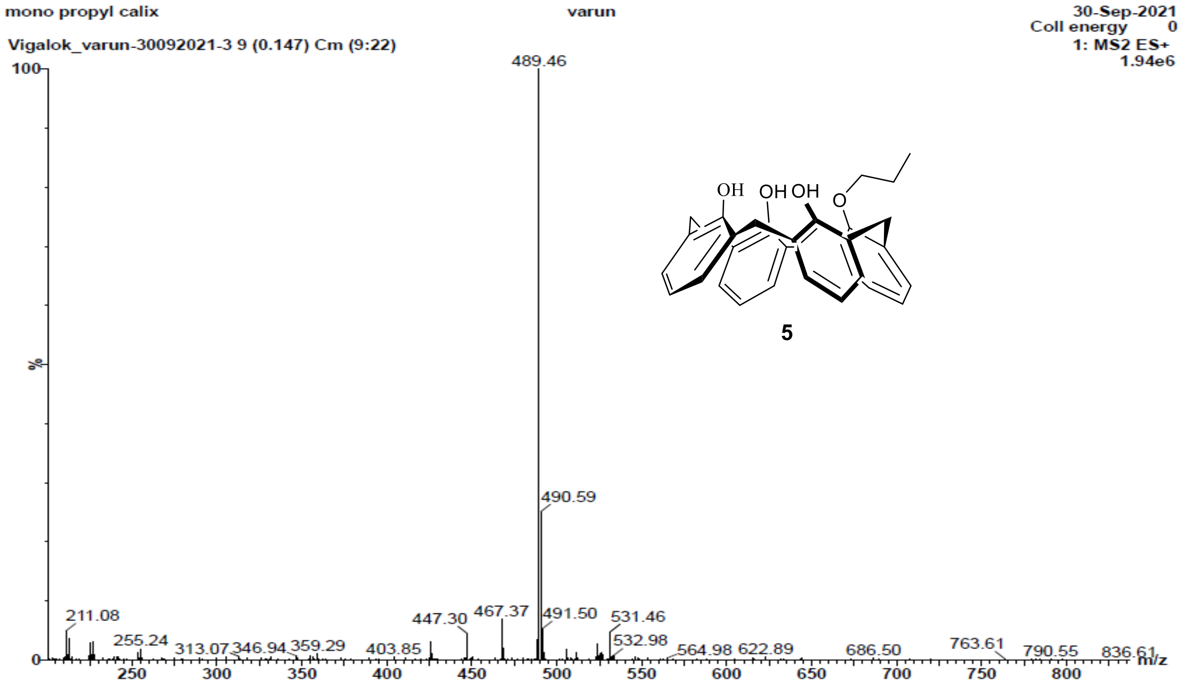

ESI MS of 5

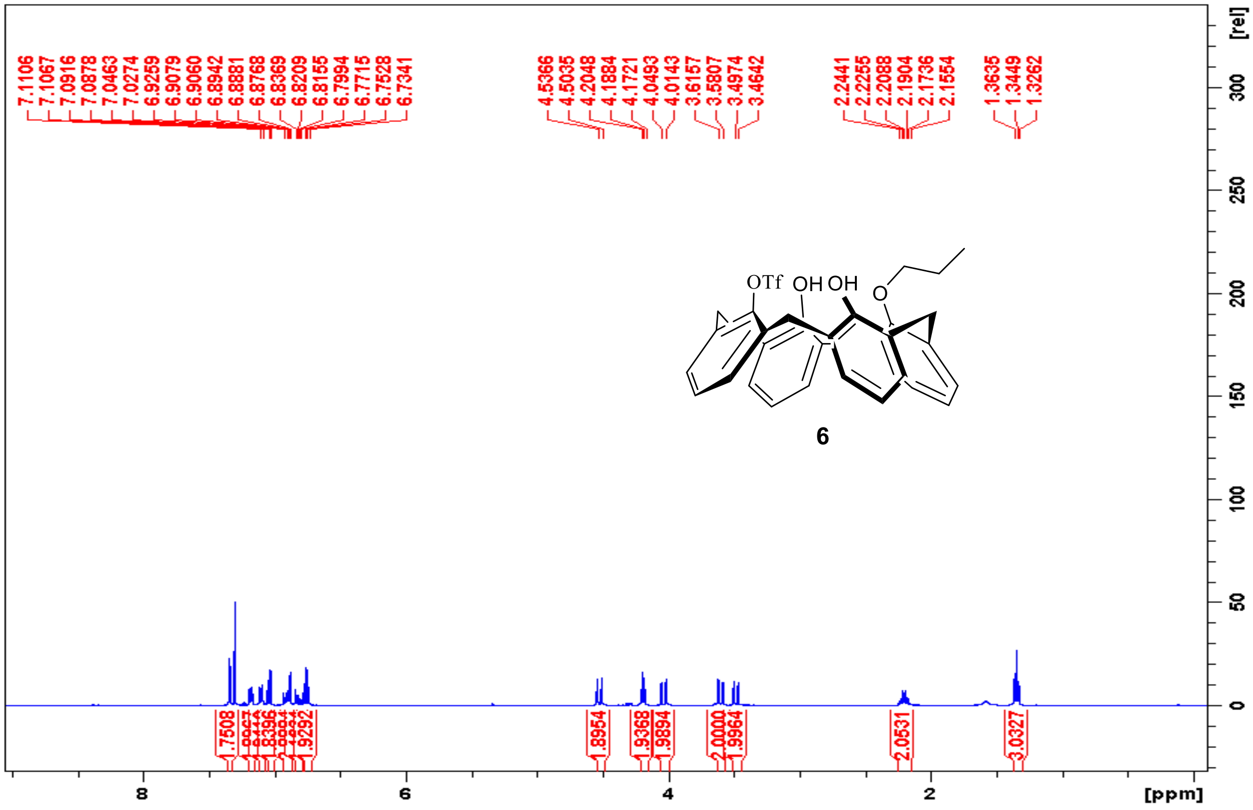

^{1}H NMR spectra of 6

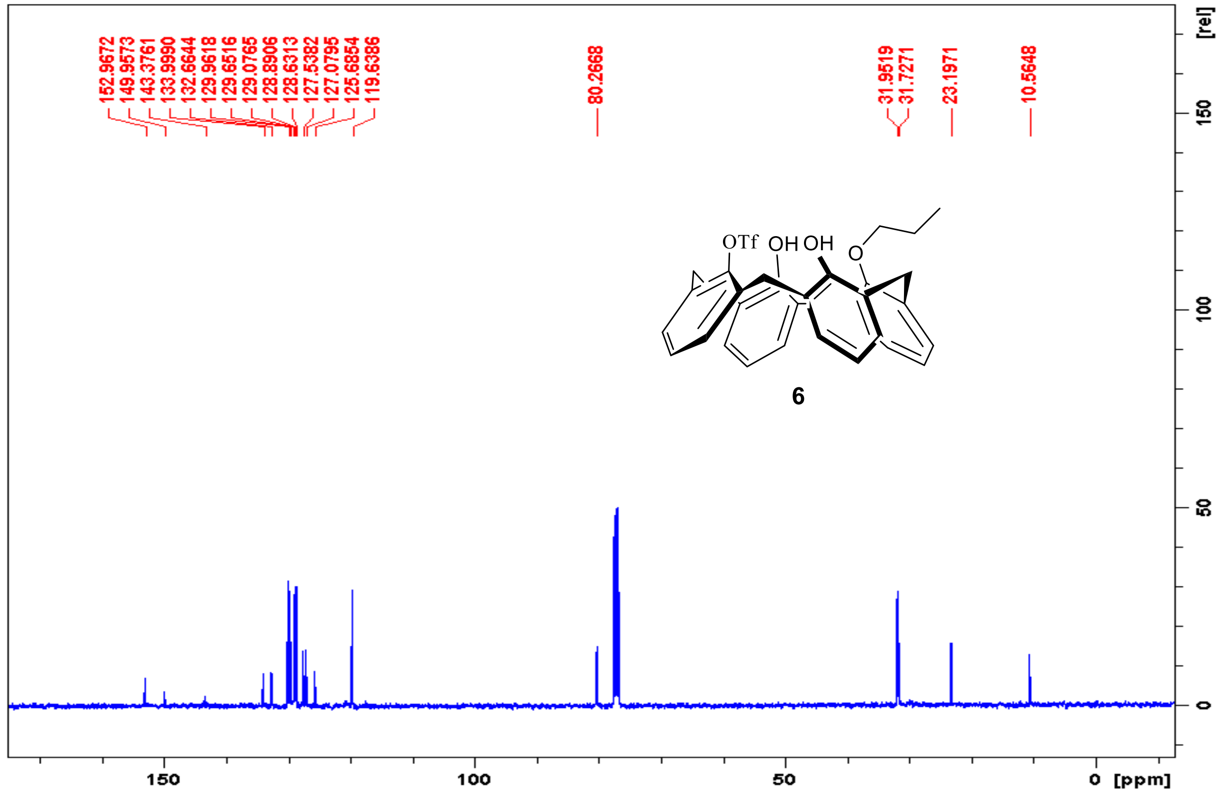

^{13}C NMR spectra of 6

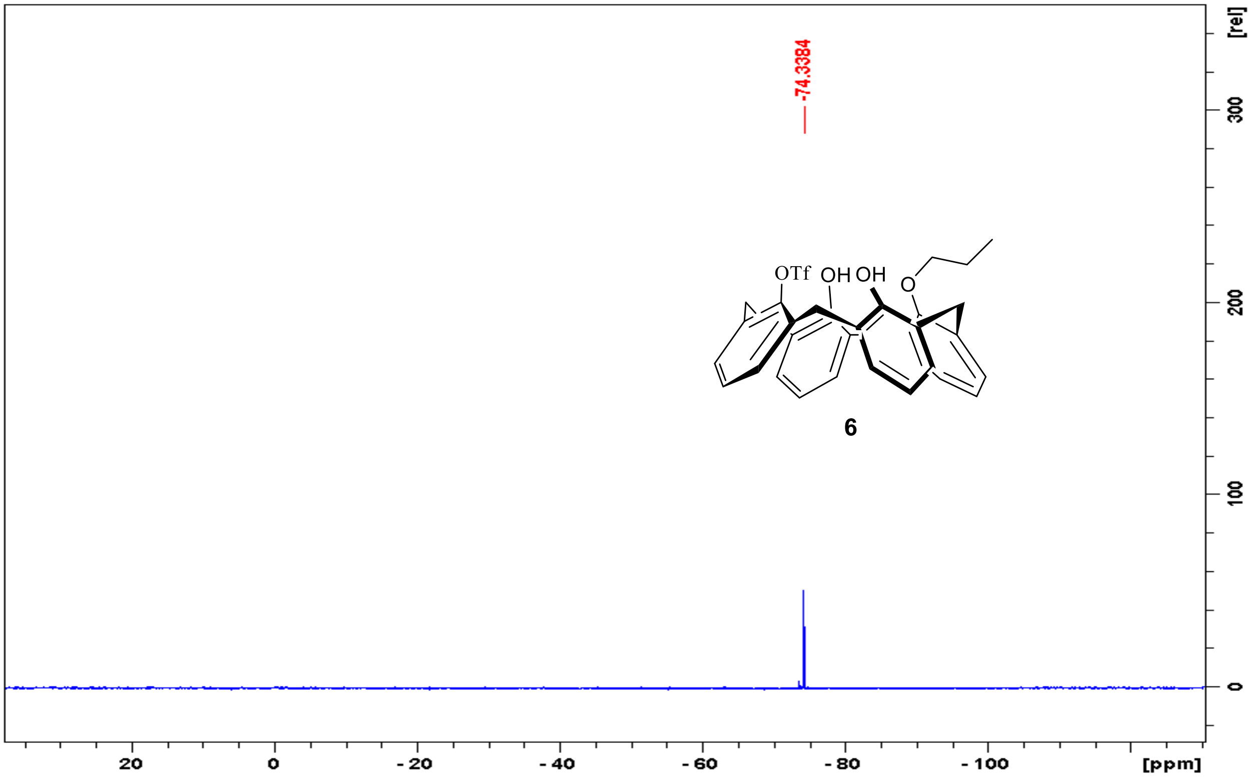

^{19}F NMR spectra of 6

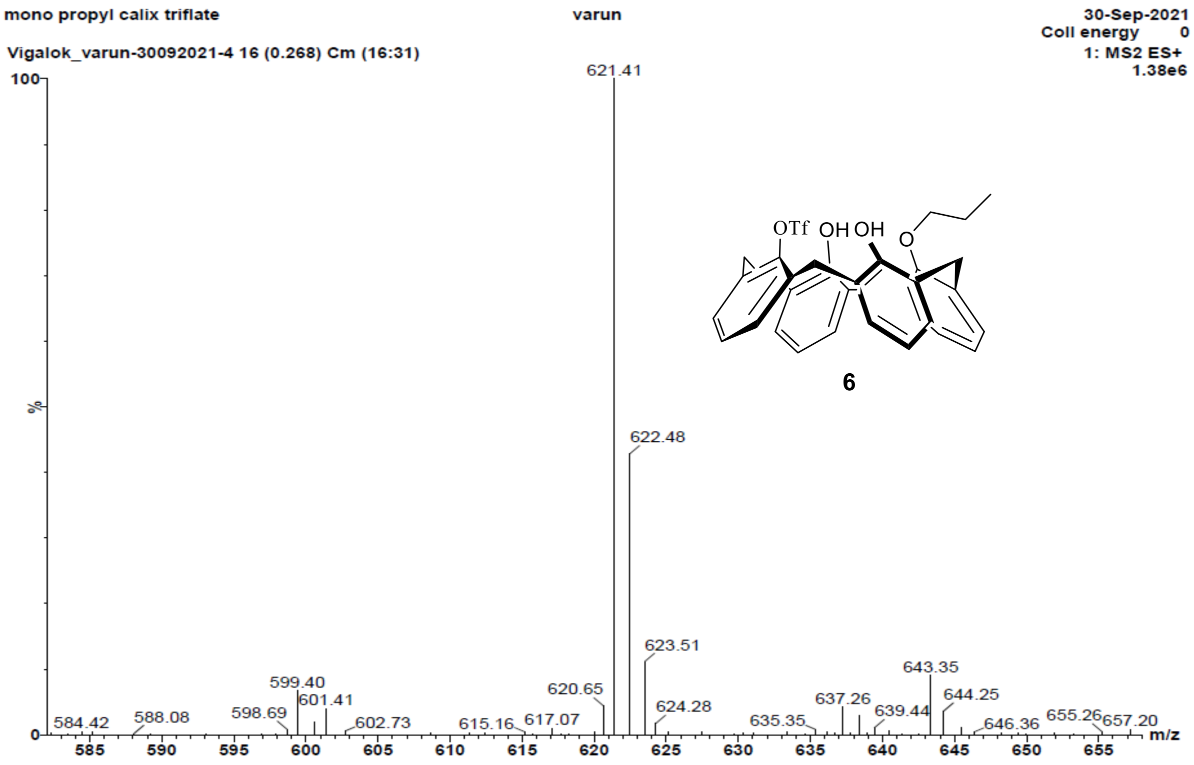

ESI MS data of 6

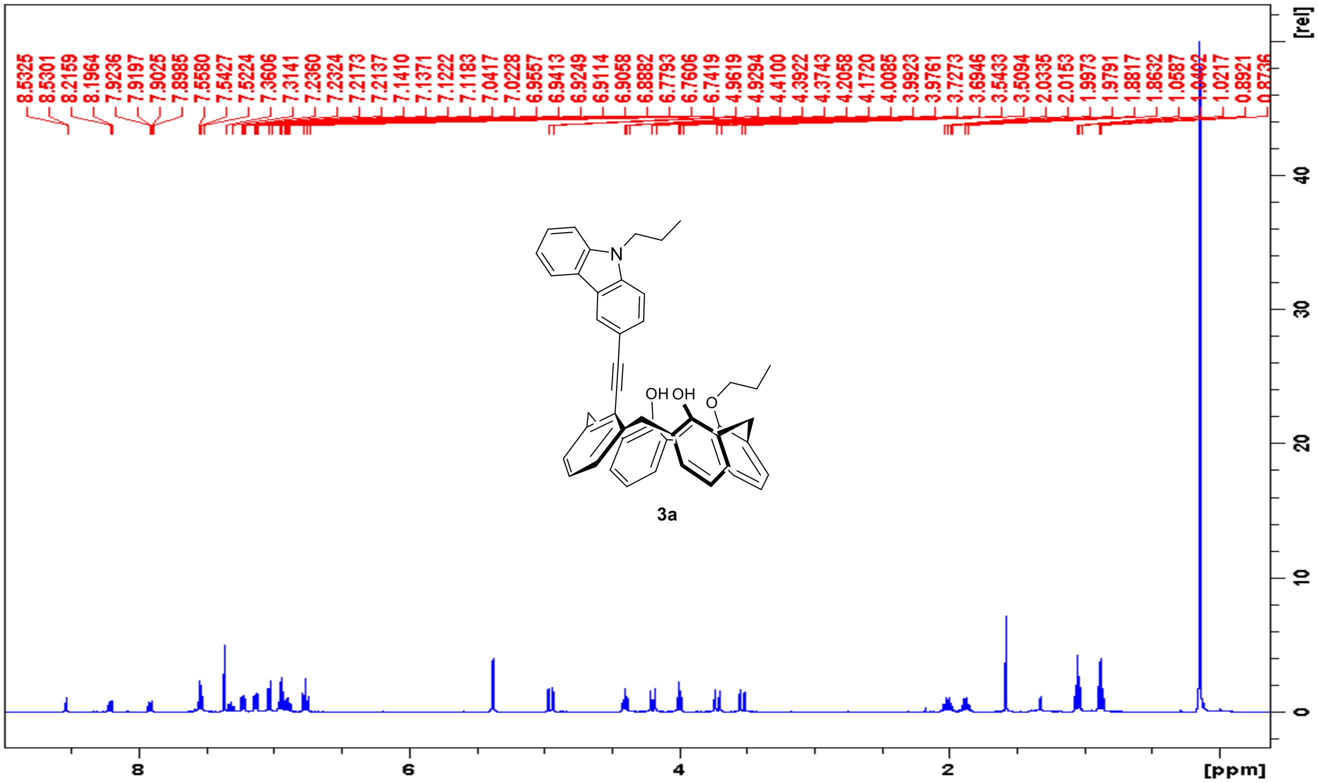

^{1}H NMR spectra of carbazole-appended calix[4]arene 3a

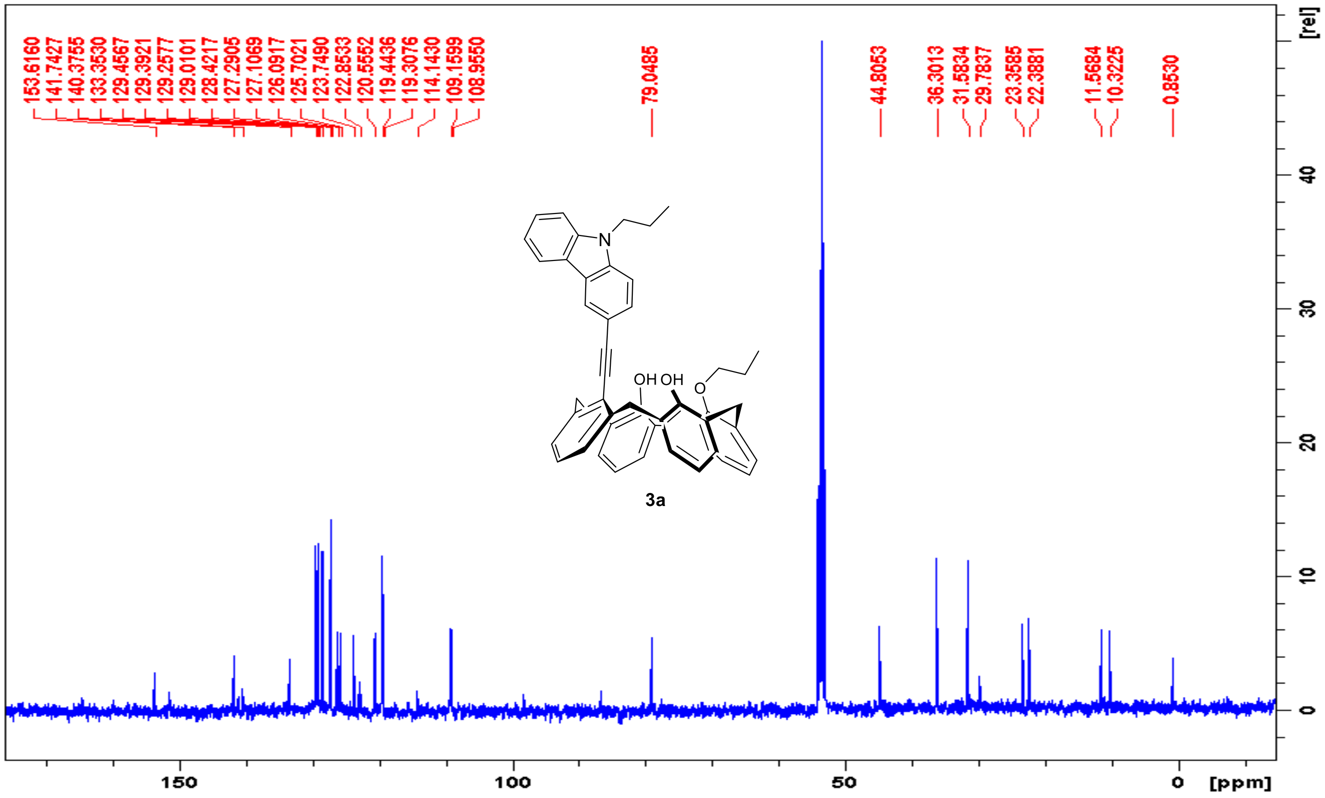

^{13}C NMR spectra of carbazole-appended calix[4]arene 3a

Single Mass Analysis
Tolerance = 10.0 PPM / DBE: min = 0.0, max = 50.0
Element prediction: Off
Number of isotope peaks used for i-FIT = 3
Monoisotopic Mass, Even Electron Ions
96 formula(e) evaluated with 2 results within limits (all results (up to 1000) for each mass)
Elements Used:

Mass	Calc. Mass	mDa	PPM	DBE	Formula	i-FIT	i-FIT Norm	Fit Conf %	C	H	N	O
682.3323	682.3321	0.2	0.3	27.5	C48 H44 N O3	993.1	3.535	2.91	48	44	1	3
	682.3281	4.2	6.2	23.5	C43 H44 N3 O5	989.6	0.030	97.09	43	44	3	5

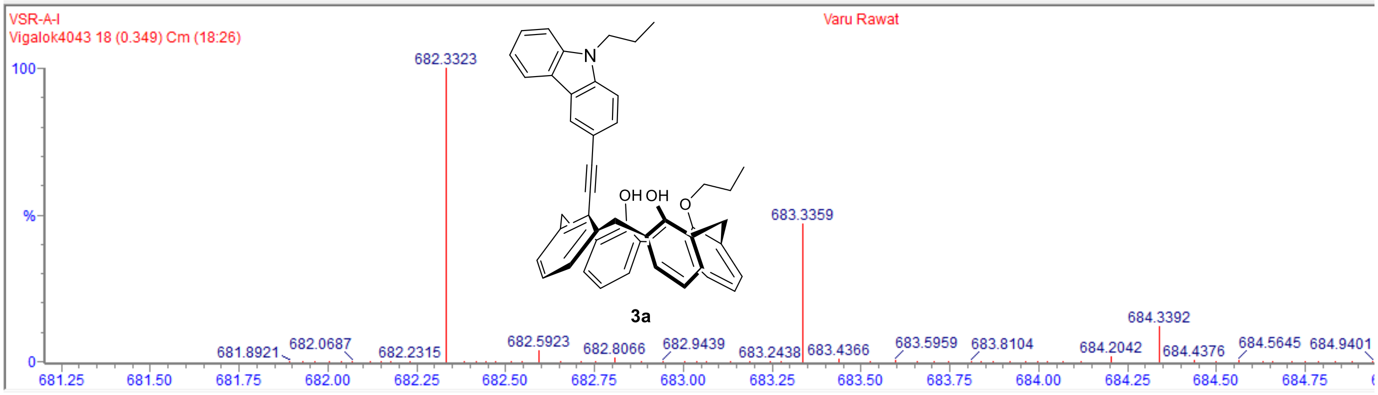

HRMS of carbazole-appended calix[4]arene 3a

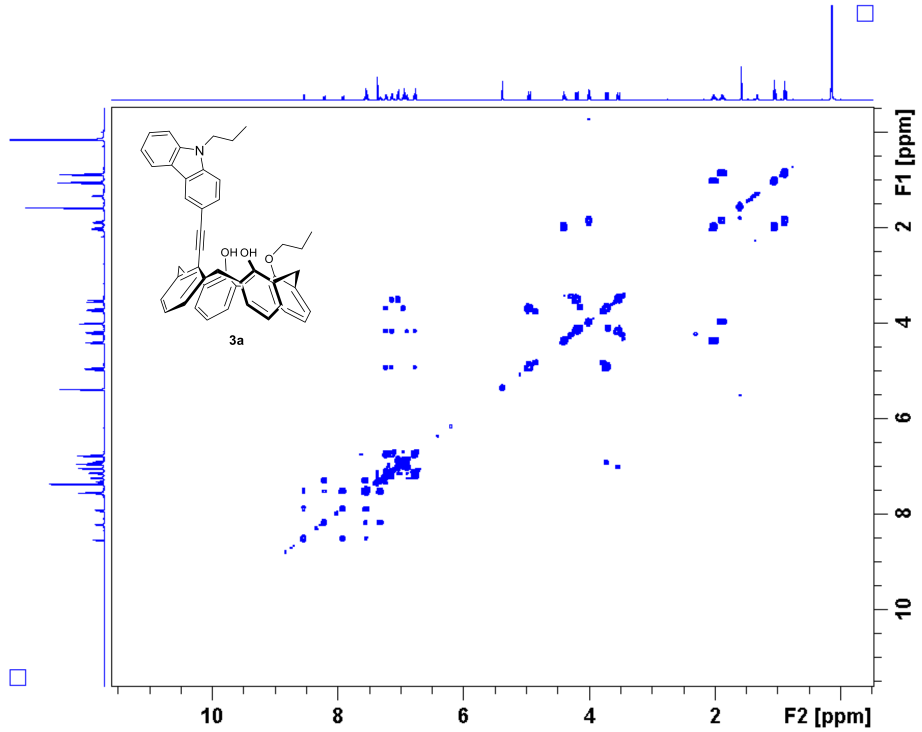

COSY NMR spectra of carbazole-appended calix[4]arene 3a

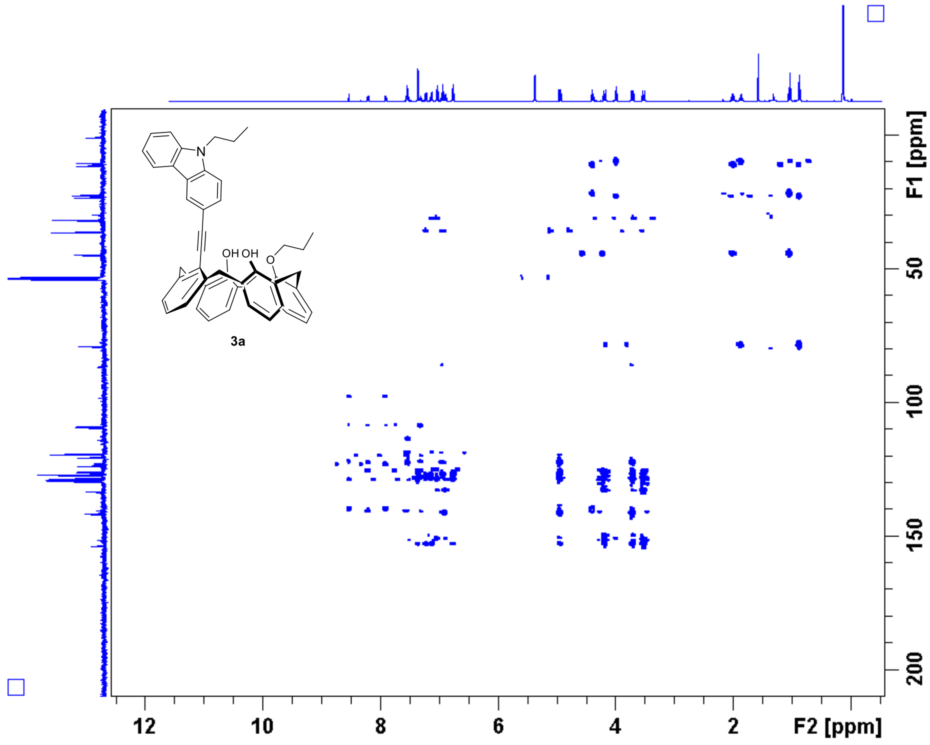

HMBC NMR spectra of carbazole-appended calix[4]arene 3a

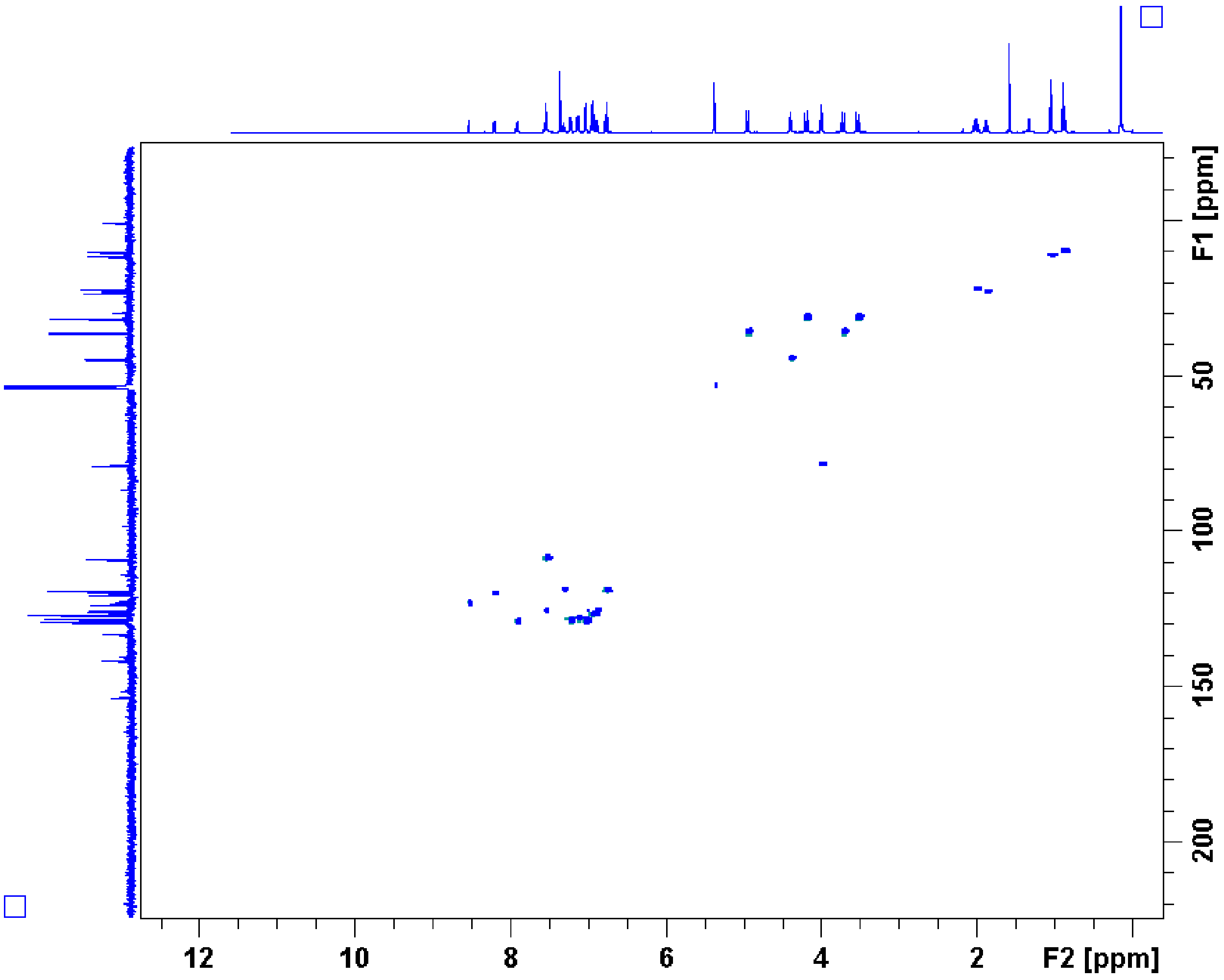

HSQC NMR spectra of carbazole-appended calix[4]arene 3a

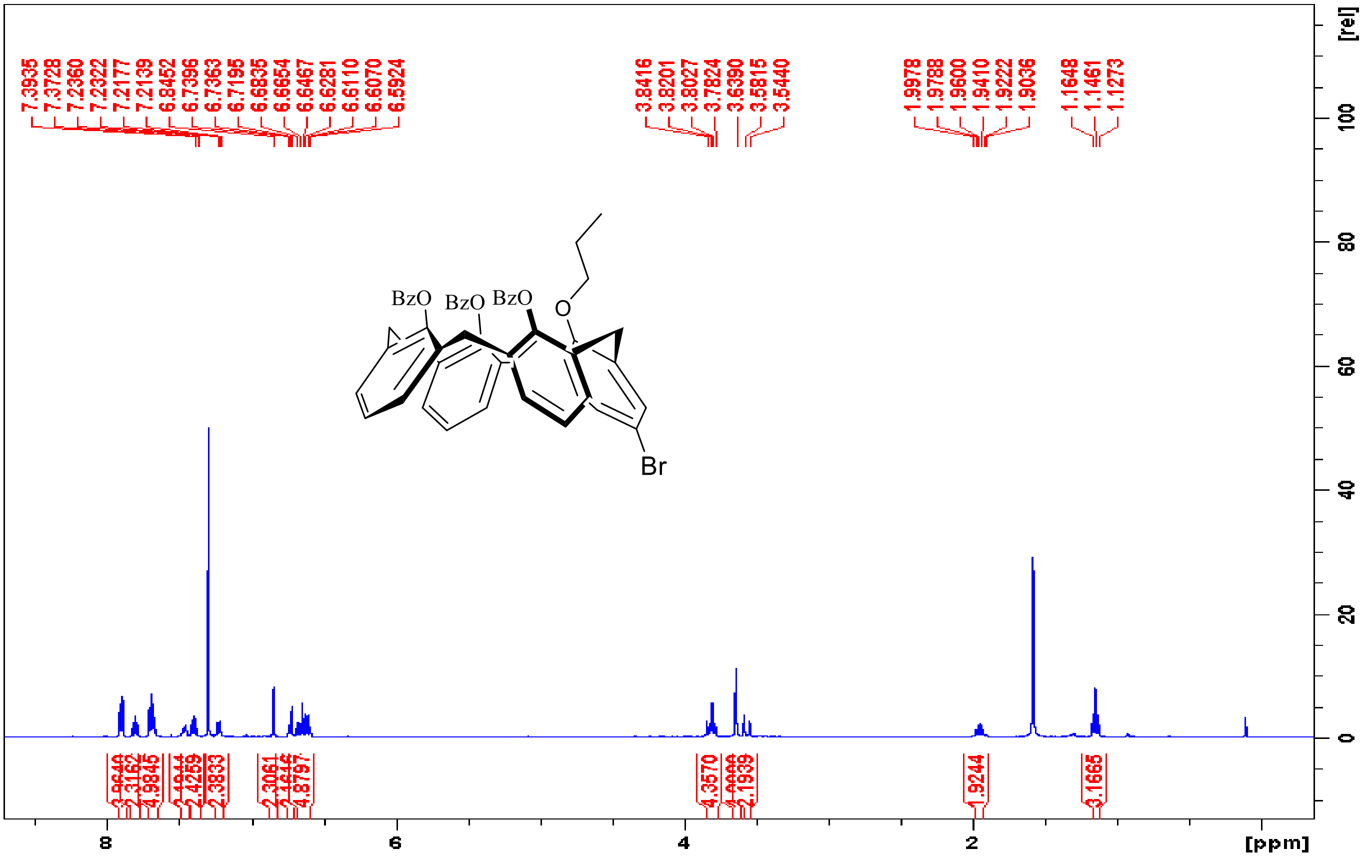

^{1}H NMR of 25,26,27-tribenzoyl-5-bromo-28-propoxy-calix[4]arene

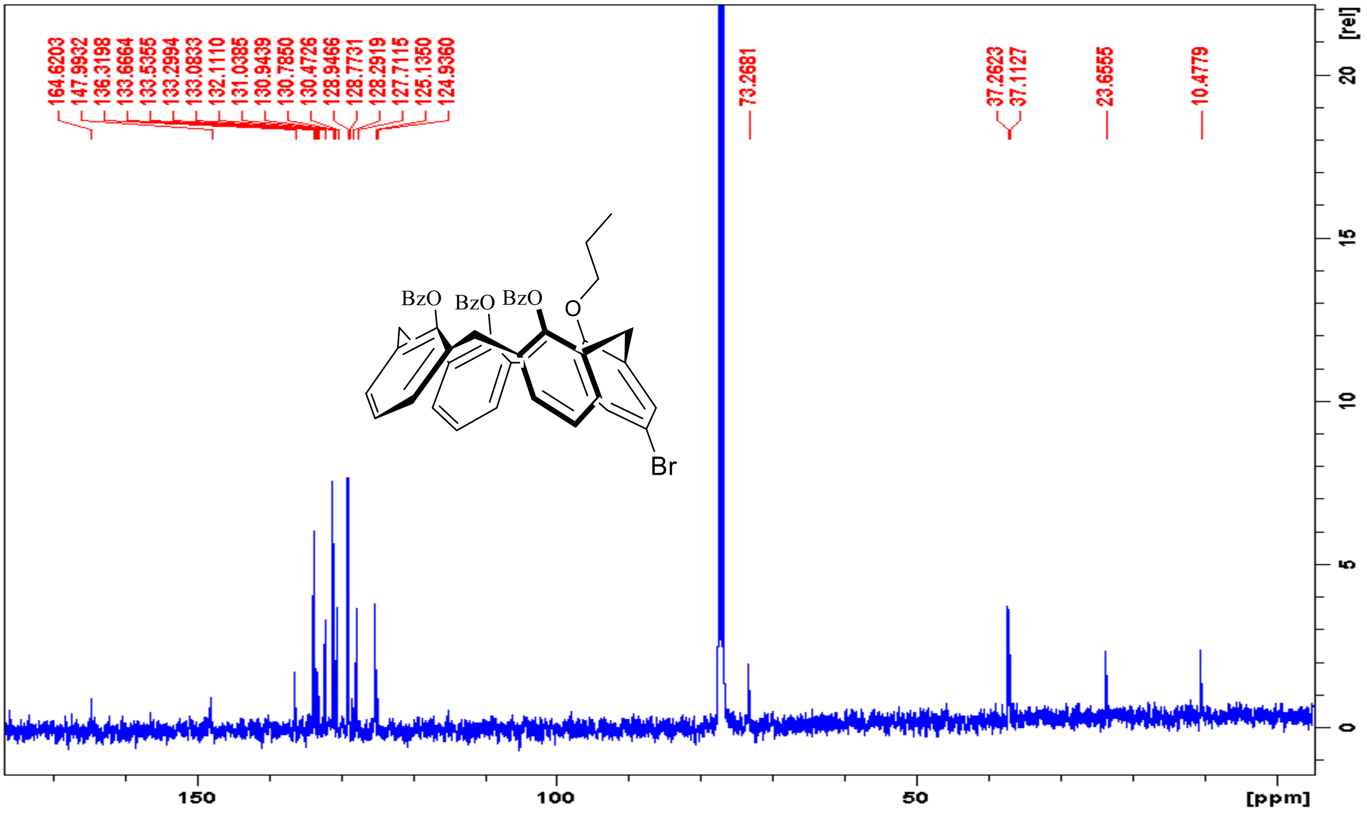

^{13}C NMR of 25,26,27-tribenzoyl-bromo-28-propoxy-calix[4]arene

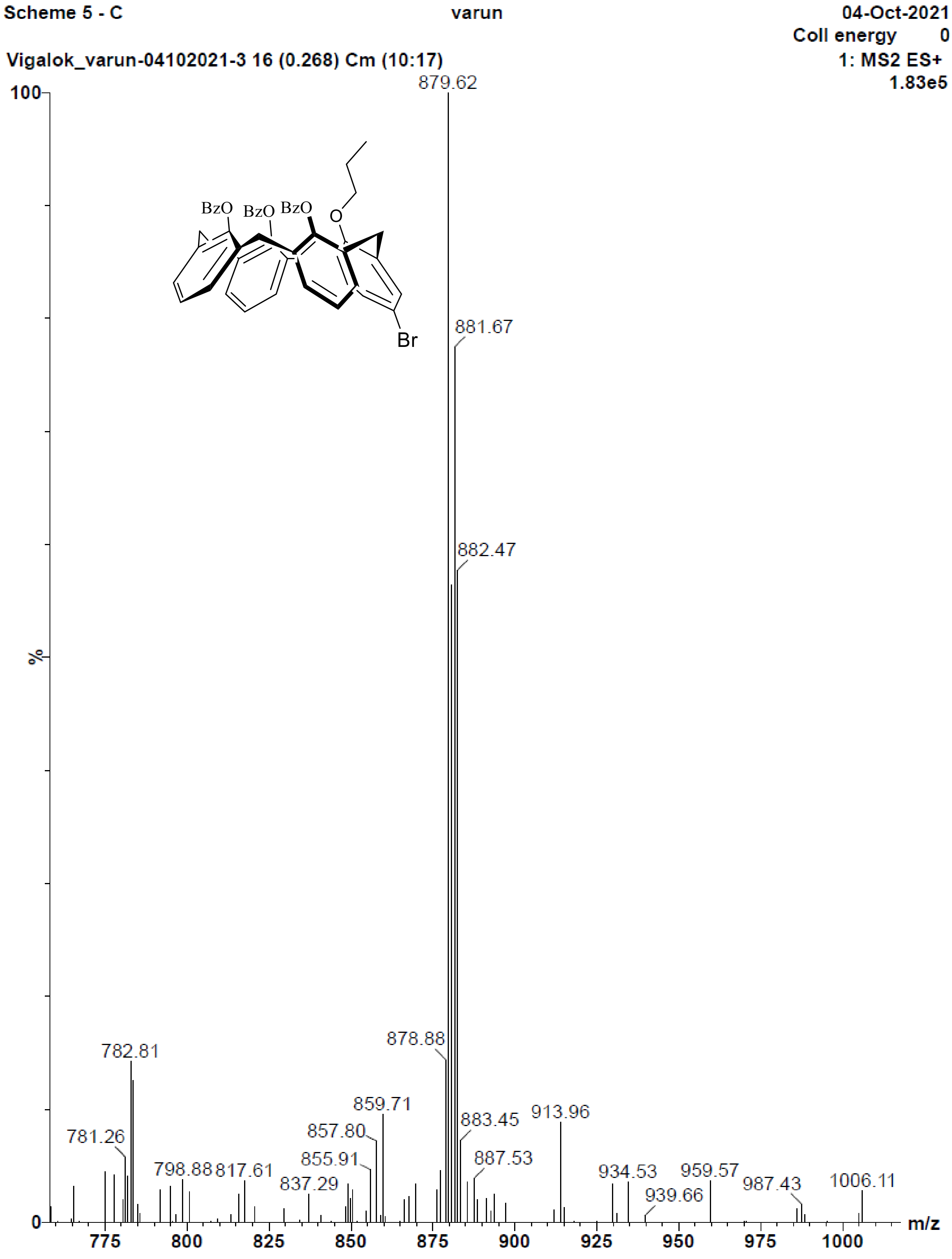

ESI MS of 25,26,27-tribenzoyl-5-bromo-28-propoxy-calix[4]arene

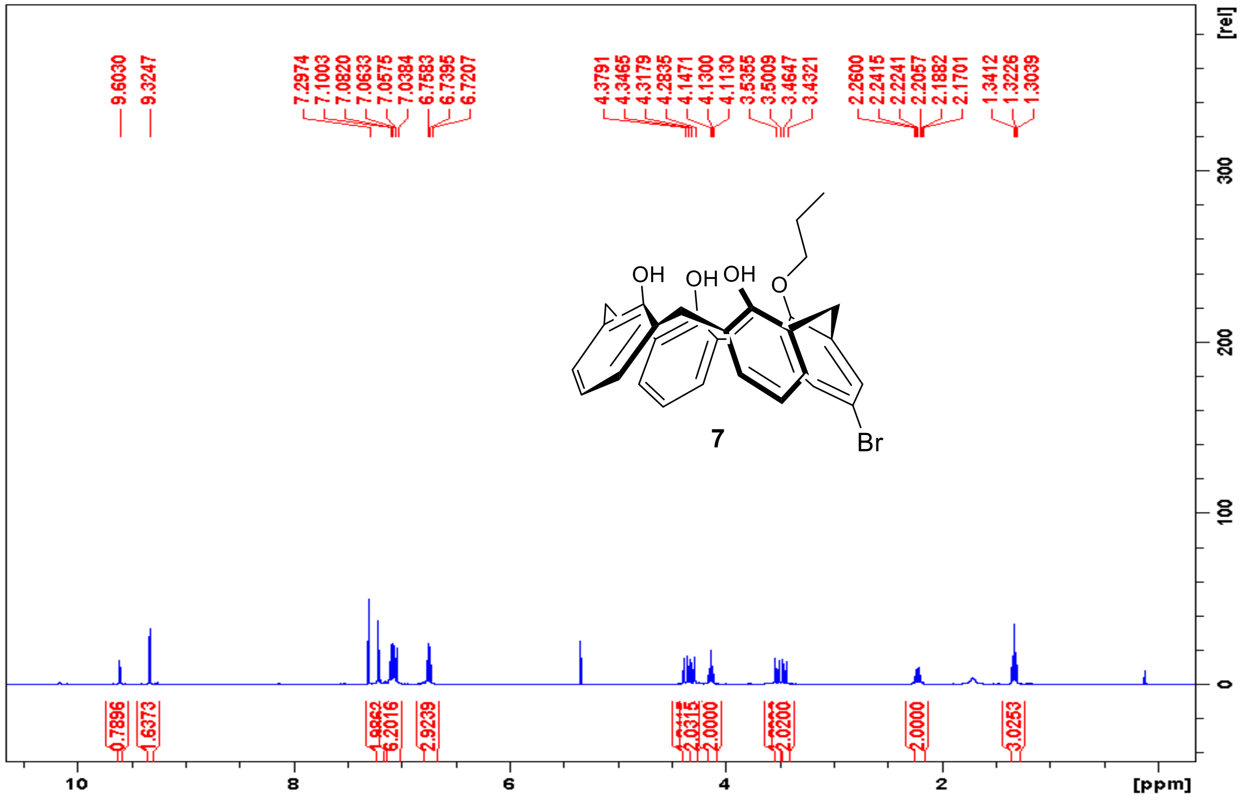

^{1}H NMR of compound 7

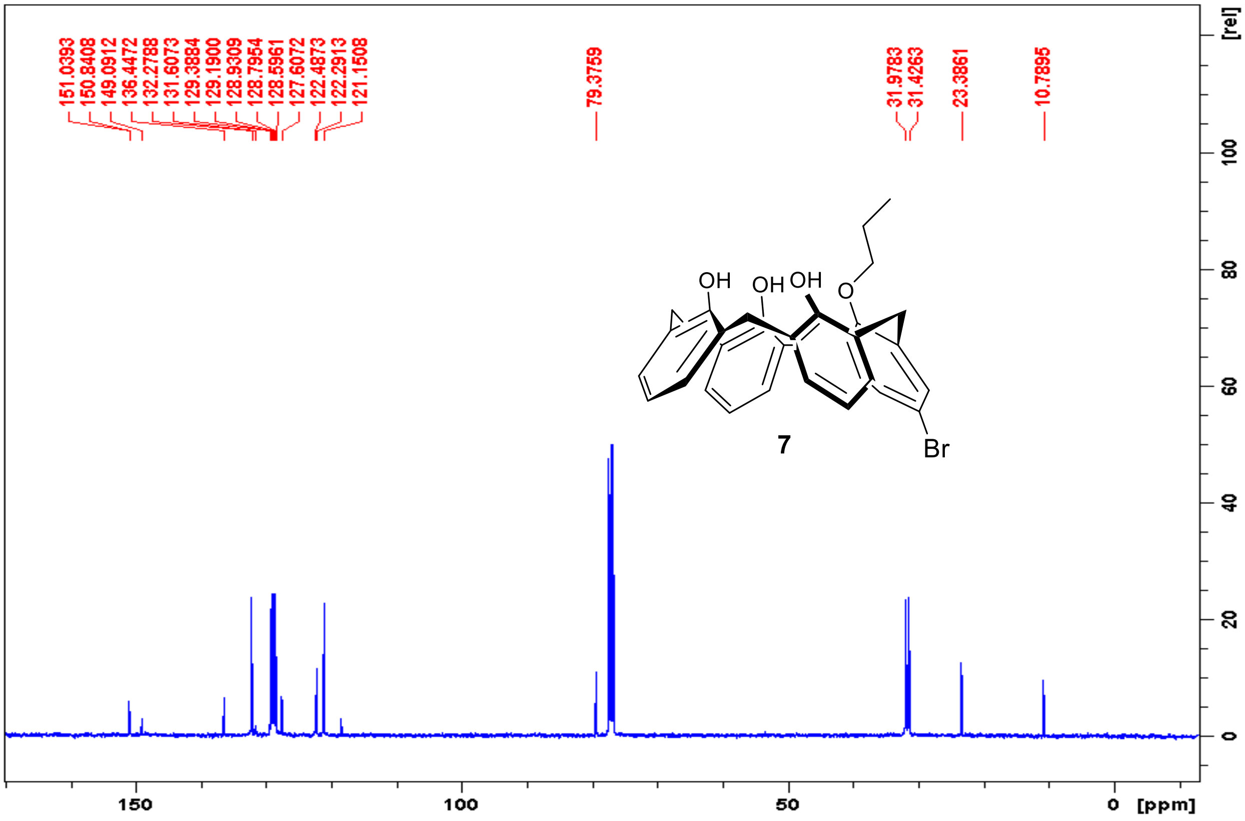

^{13}C NMR of compound 7

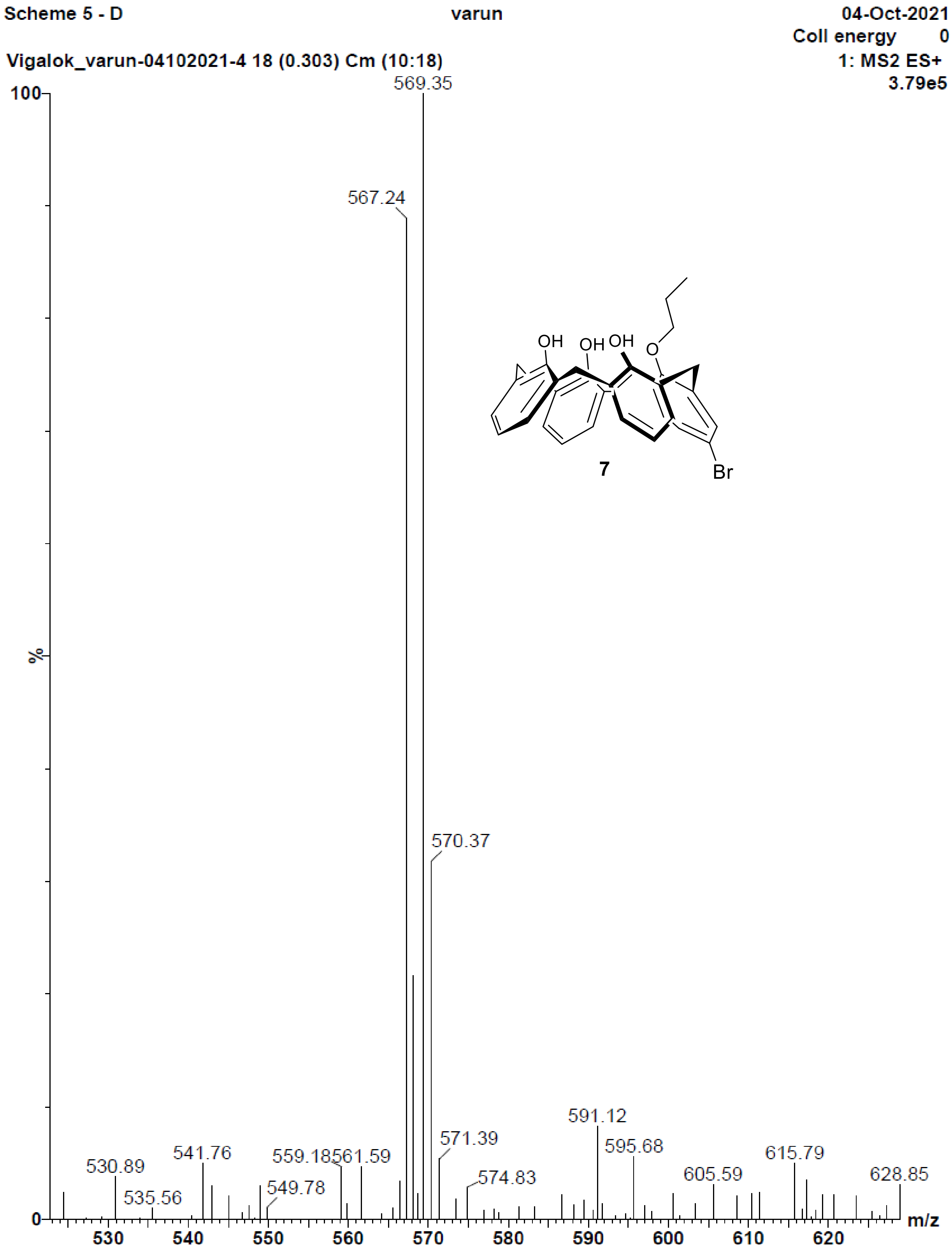
Scheme 5 - D
varun
04-Oct-2021
Coll energy 0
Vigalok_varun-04102021-4 18 (0.303) Cm (10:18)
1: MS2 ES+
3.79e5
100
%
0
569.35
567.24
570.37
571.39
591.12
595.68
541.76
530.89
535.56
559.18
561.59
549.78
574.83
605.59
615.79
628.85
530
540
550
560
570
580
590
600
610
620
m/z
OH
OH
OH
O
Br
7

ESI MS of 7

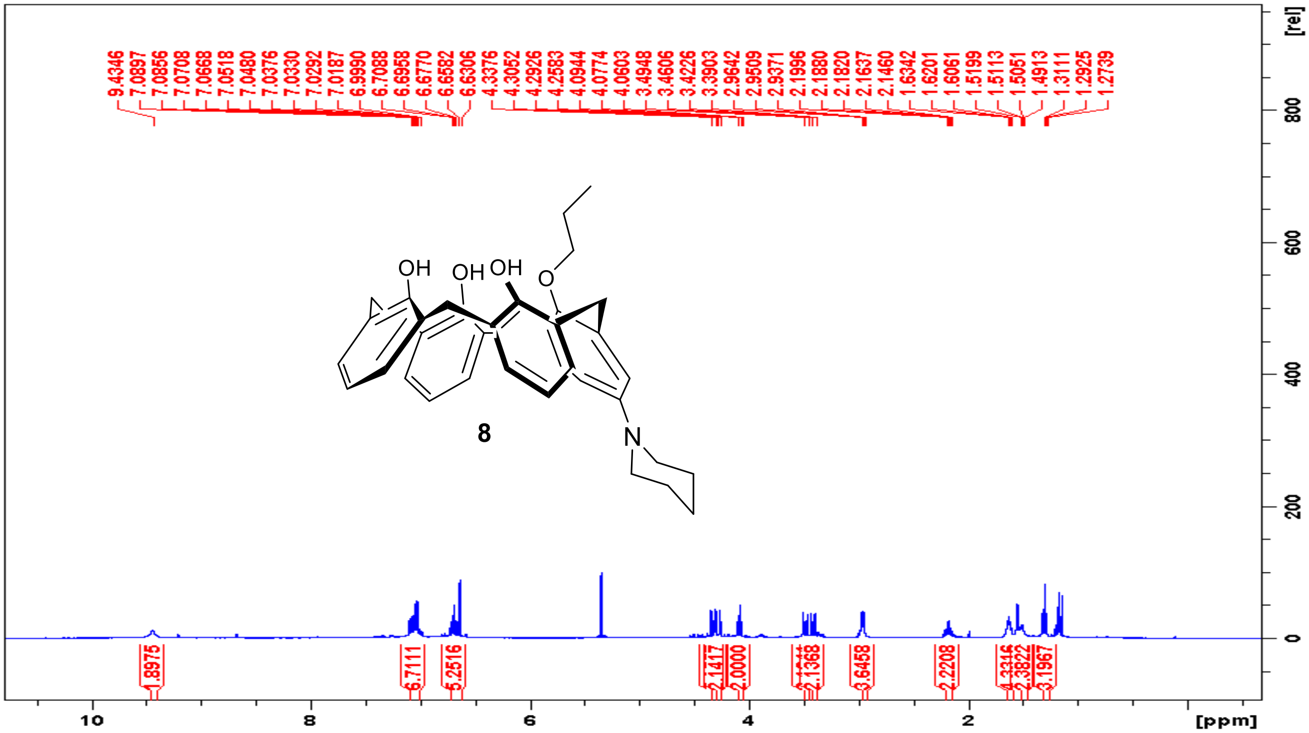

^{1}H NMR of 8

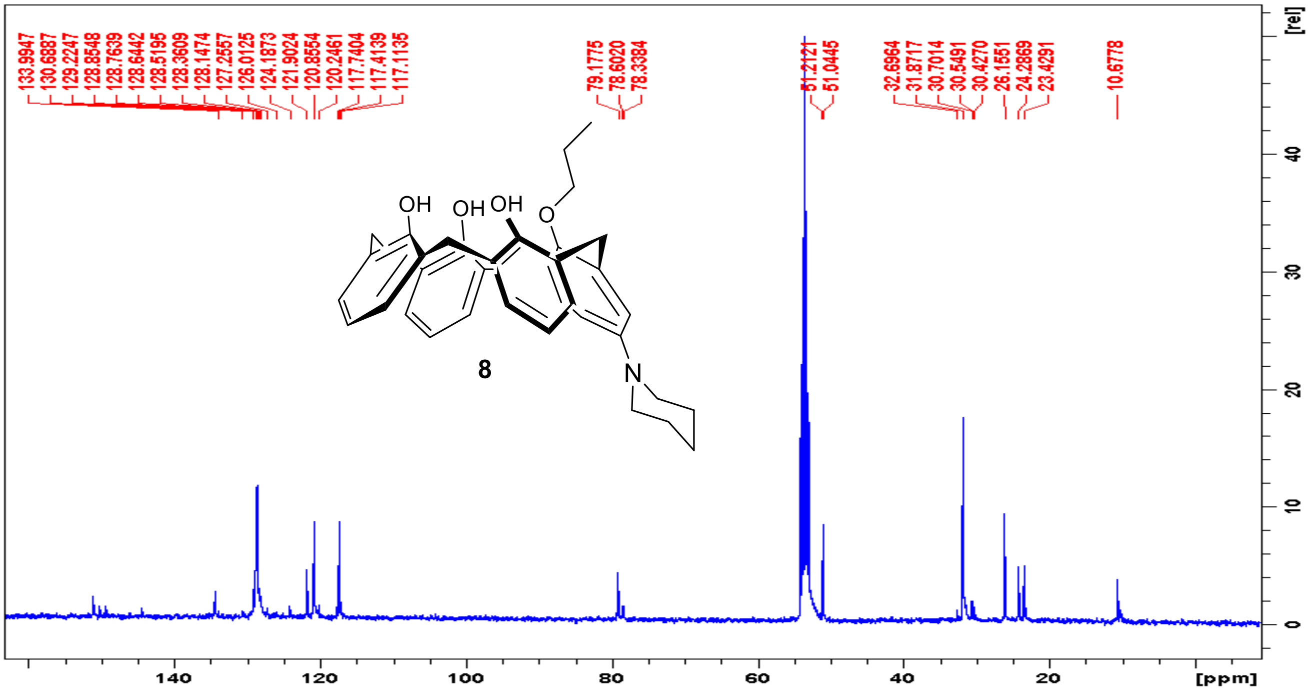

^{13}C NMR of 8

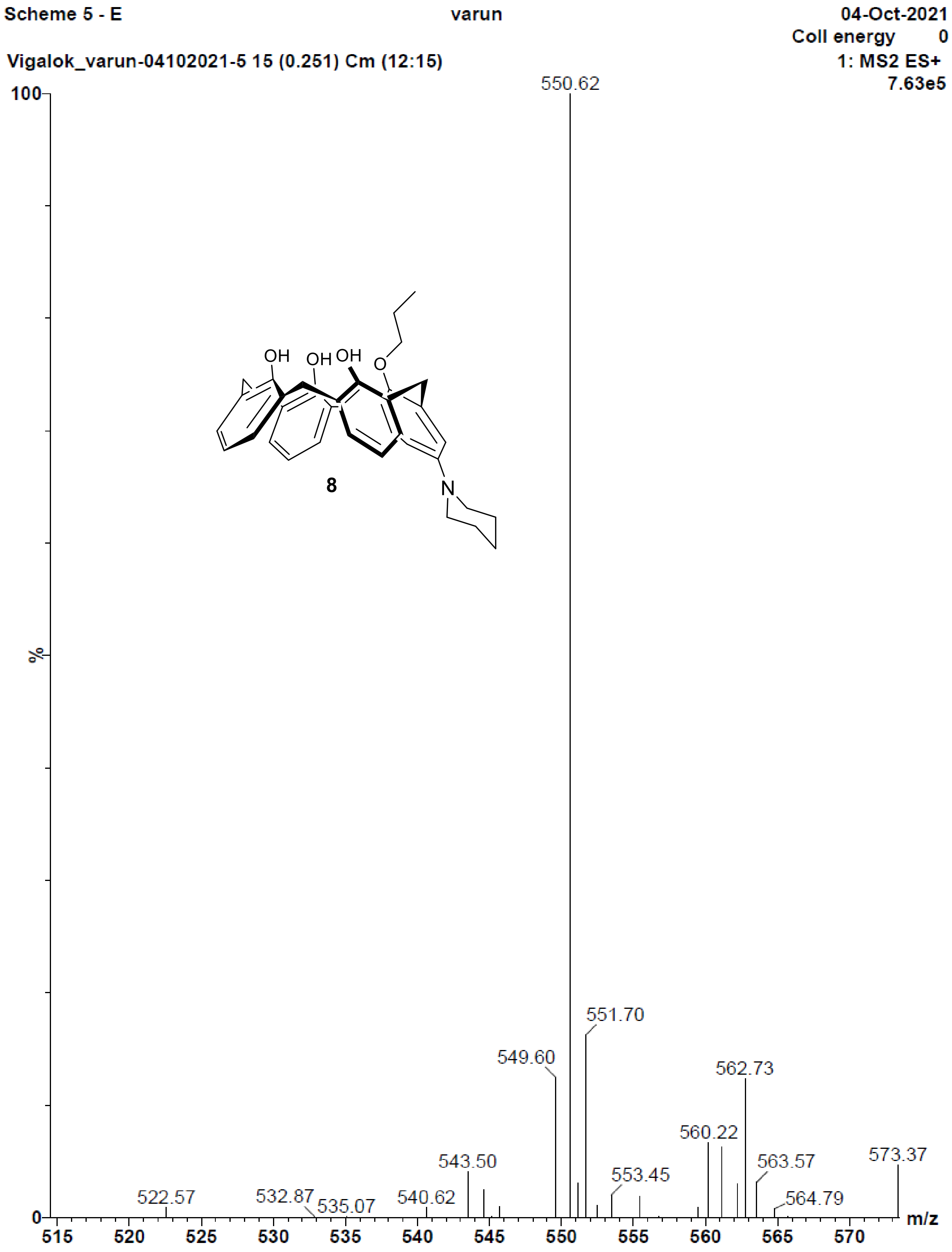
Scheme 5 - E
varun
04-Oct-2021
Coll energy 0
Vigalok_varun-04102021-5 15 (0.251) Cm (12:15)
1: MS2 ES+
7.63e5
100
%
0
OH
OH
OH
O
N
8
550.62
551.70
549.60
562.73
560.22
543.50
553.45
563.57
573.37
522.57
532.87
535.07
540.62
564.79
m/z
515
520
525
530
535
540
545
550
555
560
565
570

ESI MS of 8

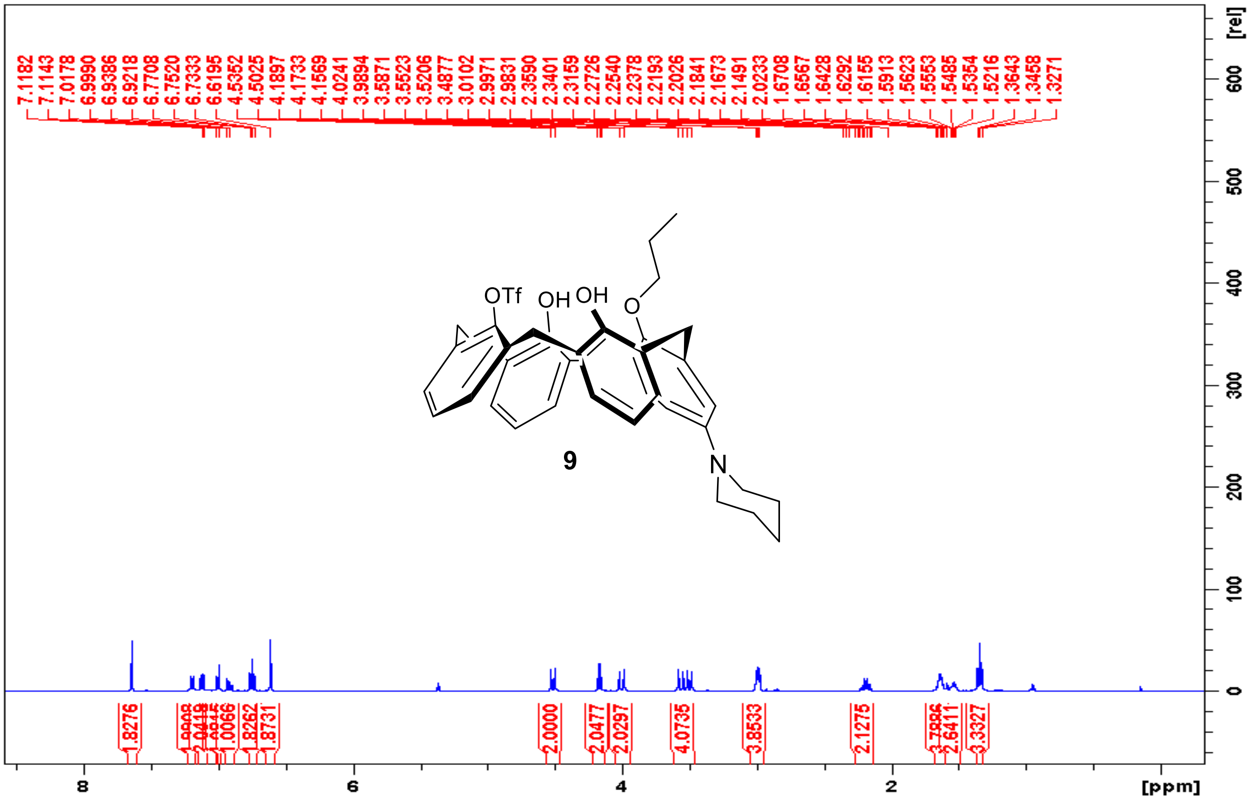

^{1}H NMR of 9

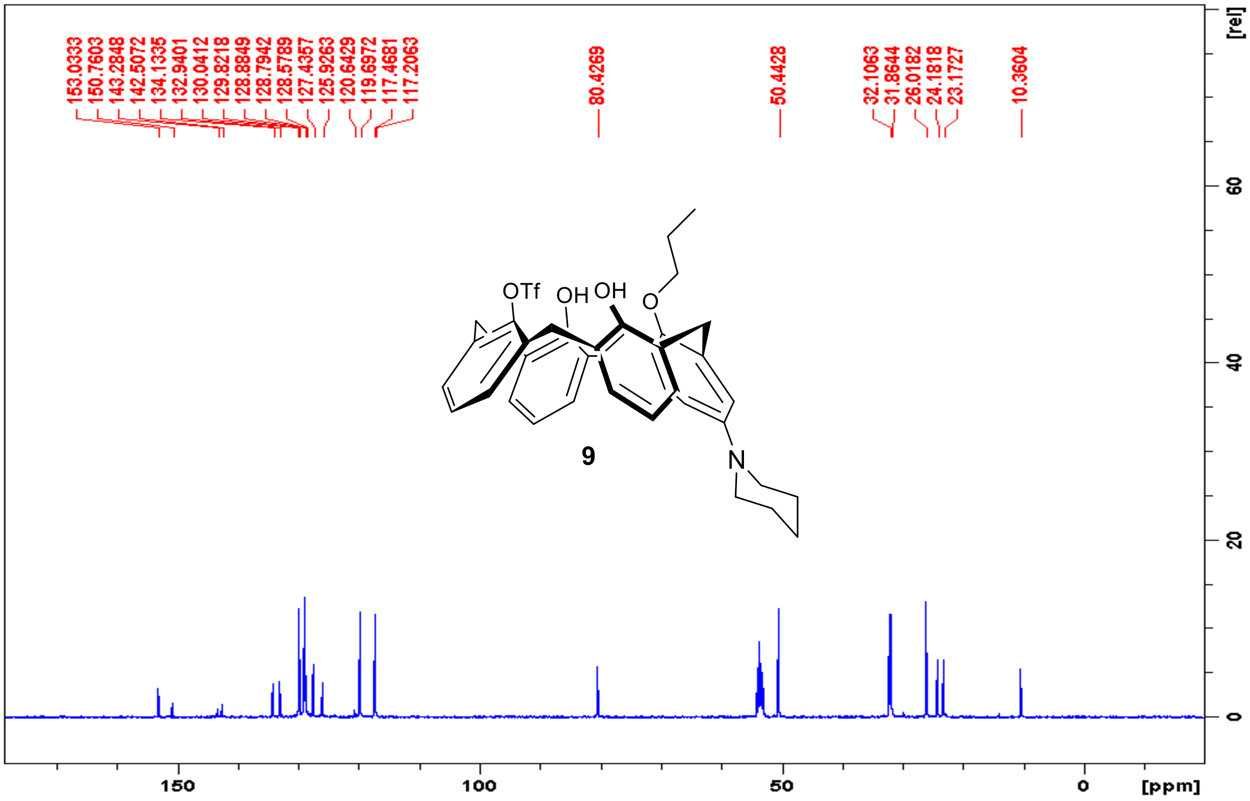

^{13}C NMR of 9

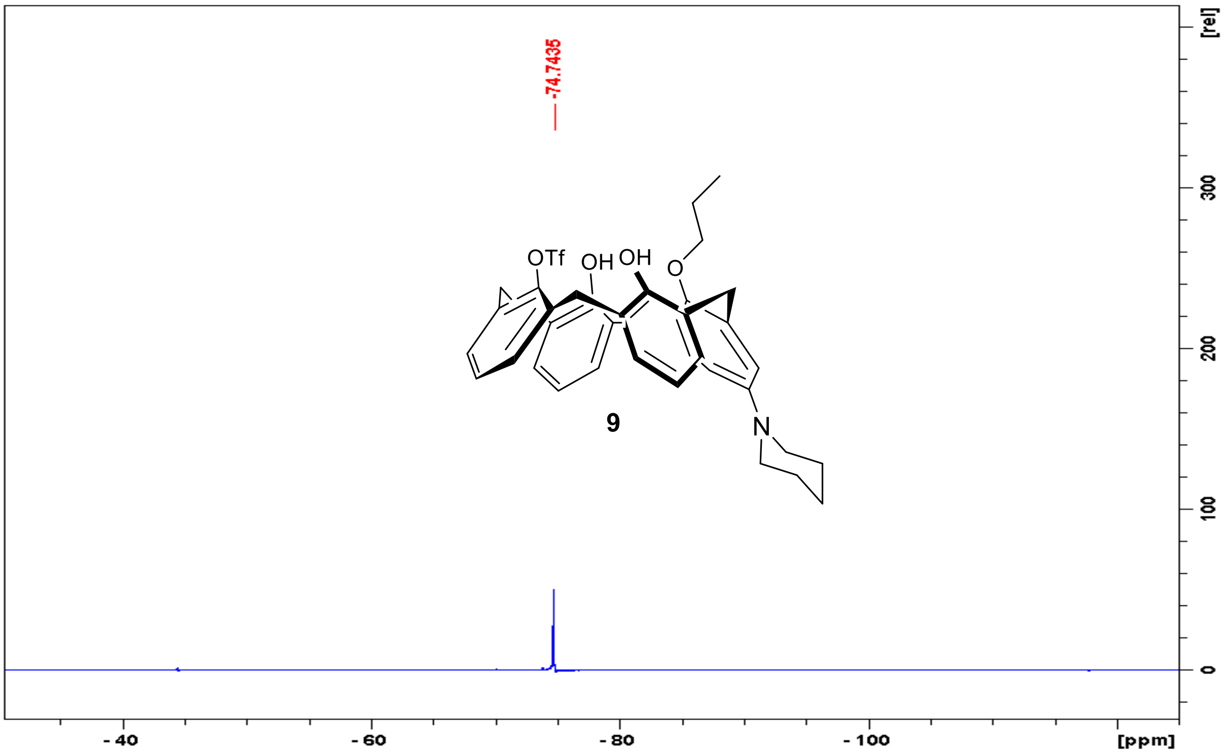

^{19}F NMR of 9

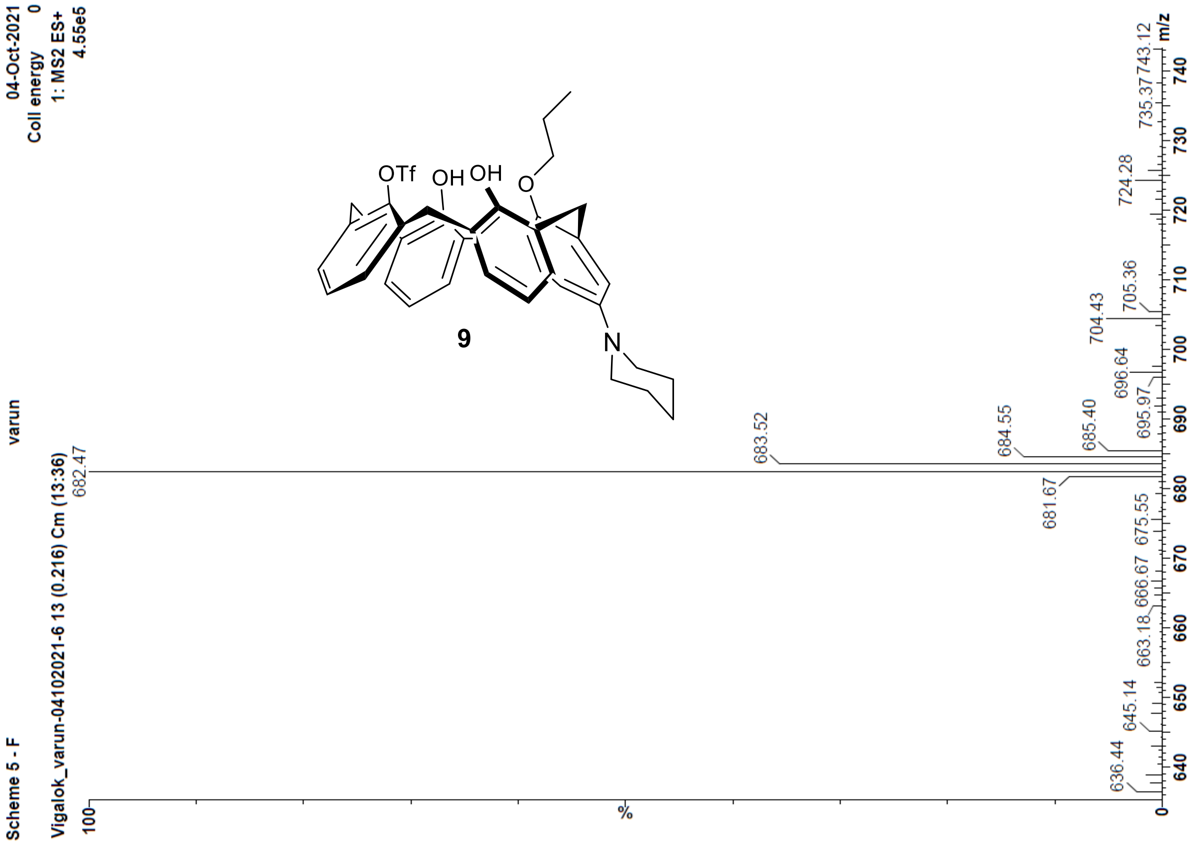

ESI MS of 9

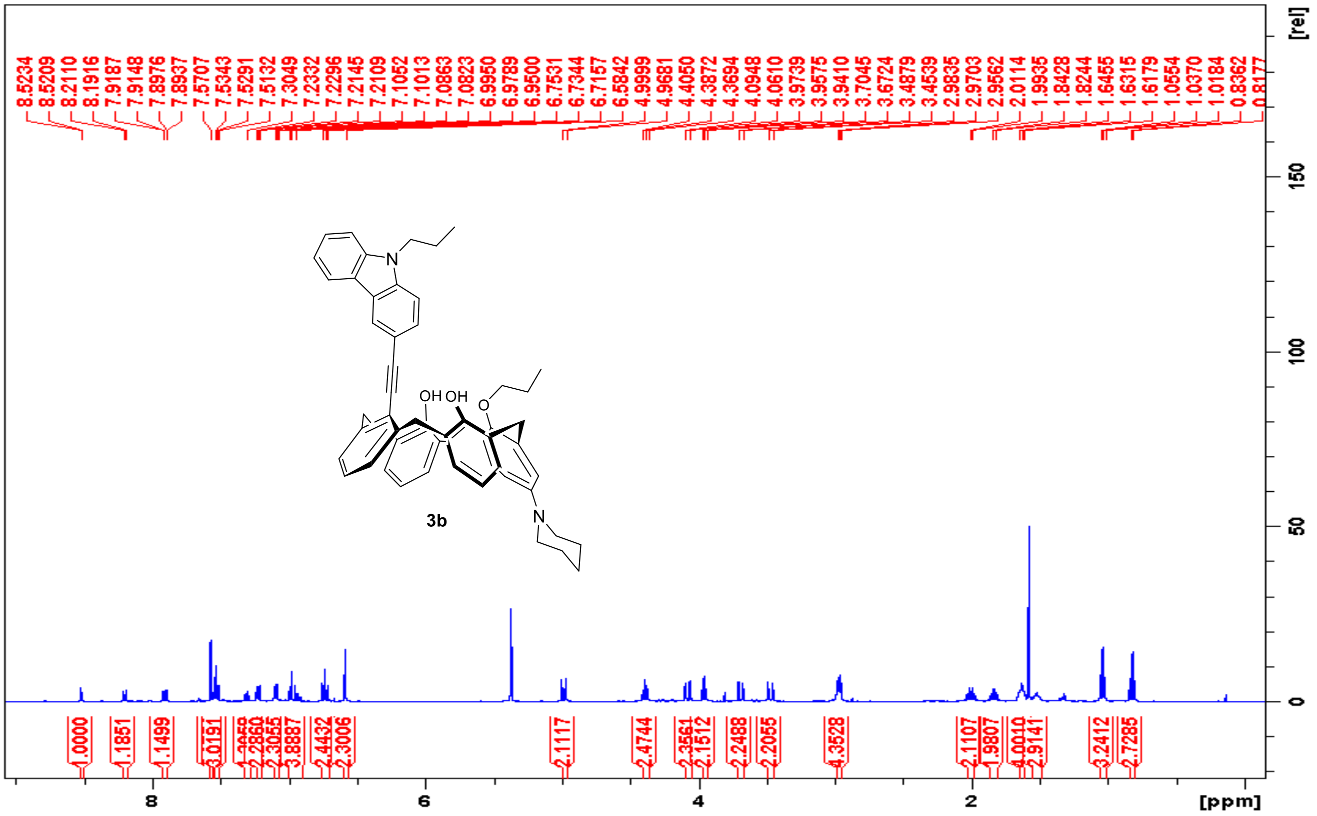

^{1}H NMR of carbazole-appended calix[4]arene 3b

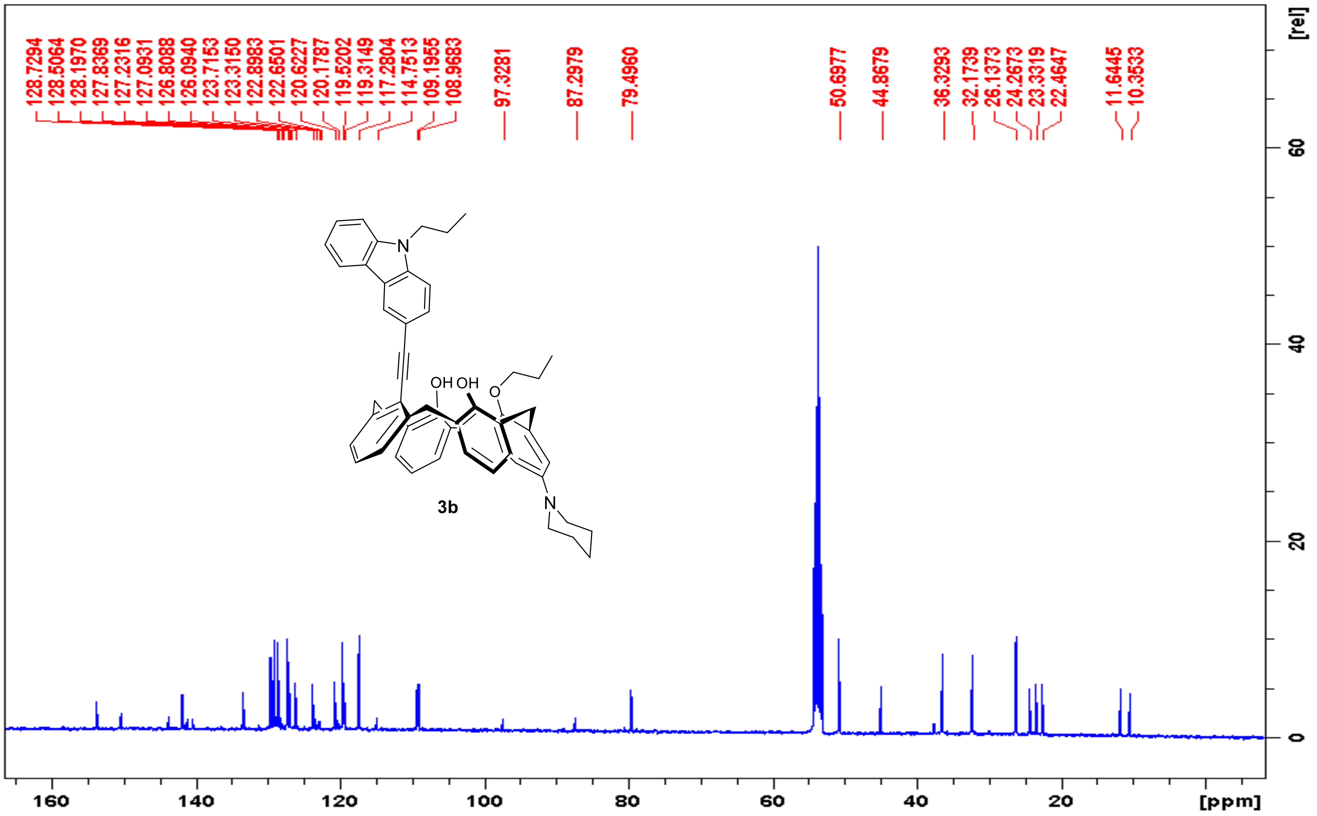

^{13}C NMR of carbazole-appended calix[4]arene 3b

Single Mass Analysis
Tolerance = 10.0 PPM / DBE: min = 0.0, max = 50.0
Element prediction: Off
Number of isotope peaks used for i-FIT = 3
Monoisotopic Mass, Even Electron Ions
89 formula(e) evaluated with 3 results within limits (all results (up to 1000) for each mass)
Elements Used:

Mass	Calc. Mass	mDa	PPM	DBE	Formula	i-FIT	i-FIT Norm	Fit Conf %	C	H	N	O
765.4055	765.4056	-0.1	-0.1	28.5	C53 H53 N2 O3	955.0	0.222	80.10	53	53	2	3
	765.4016	3.9	5.1	24.5	C48 H53 N4 O5	956.4	1.636	19.48	48	53	4	5
	765.4096	-4.1	-5.4	32.5	C58 H53 O	960.2	5.478	0.42	58	53		1

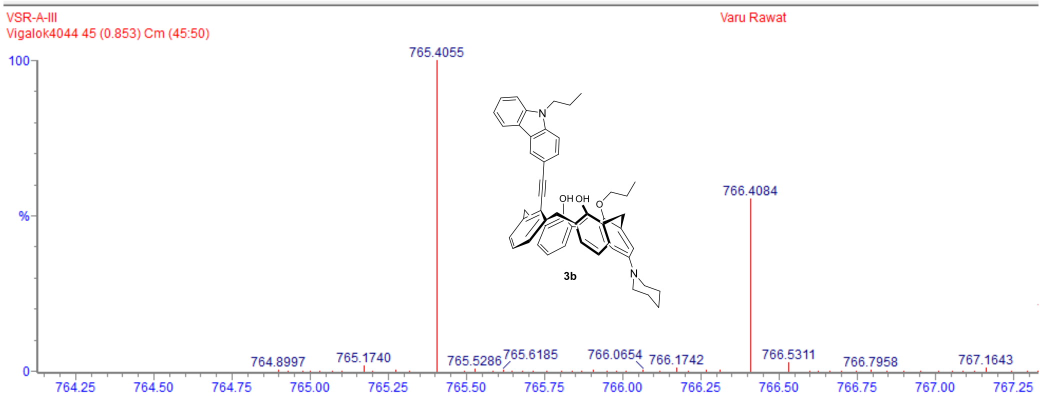

HRMS of carbazole-appended calix[4]arene 3b

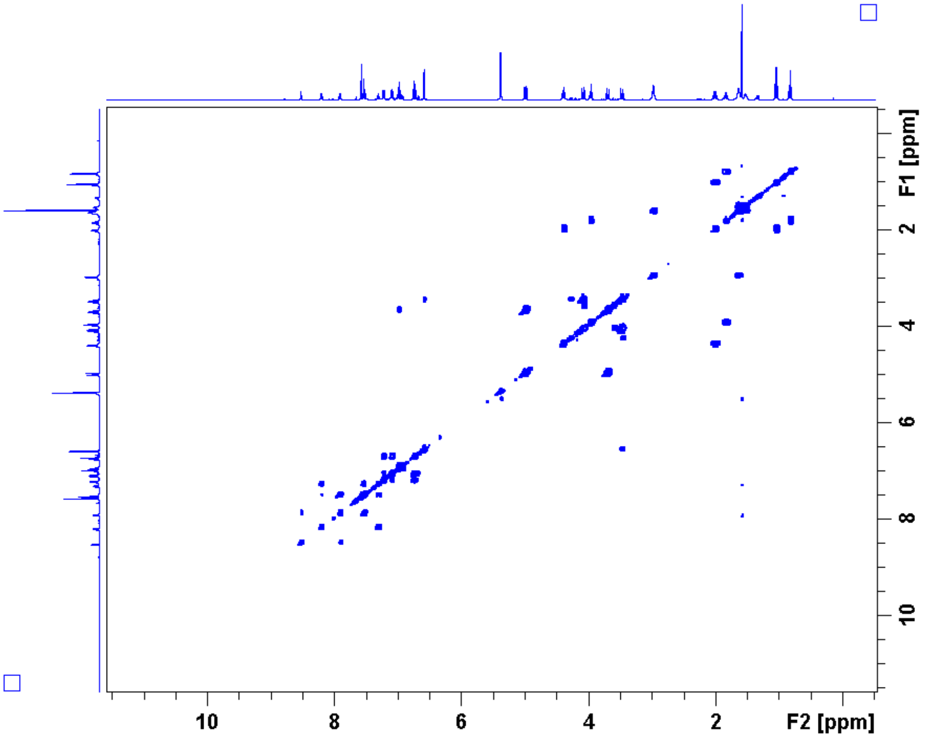

COSY NMR of carbazole-appended calix[4]arene 3b

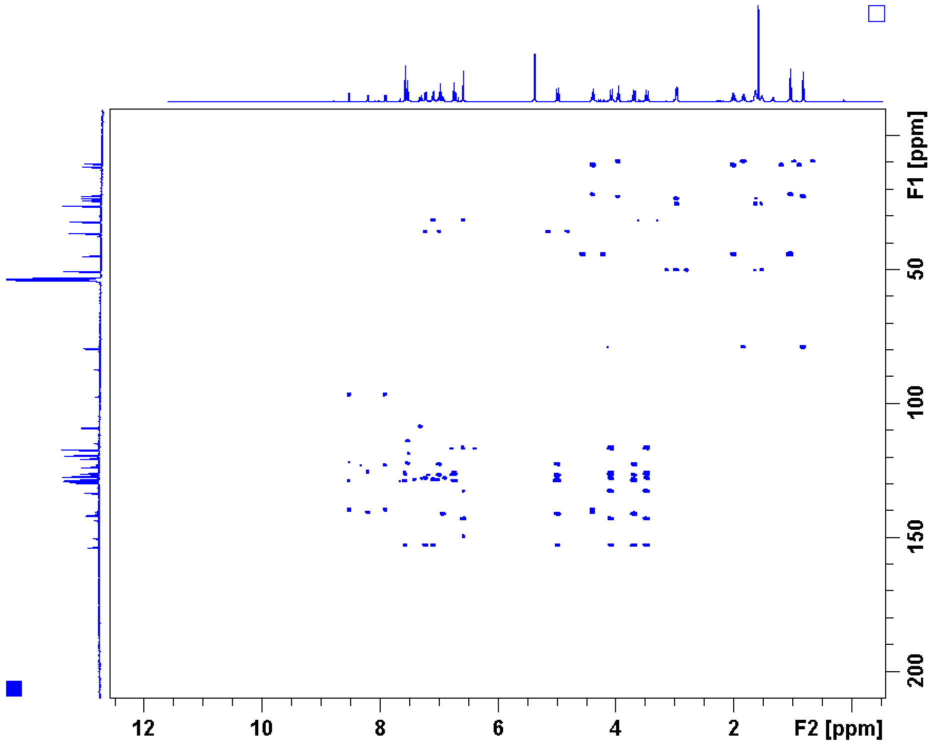

HMBC NMR of carbazole-appended calix[4]arene 3b

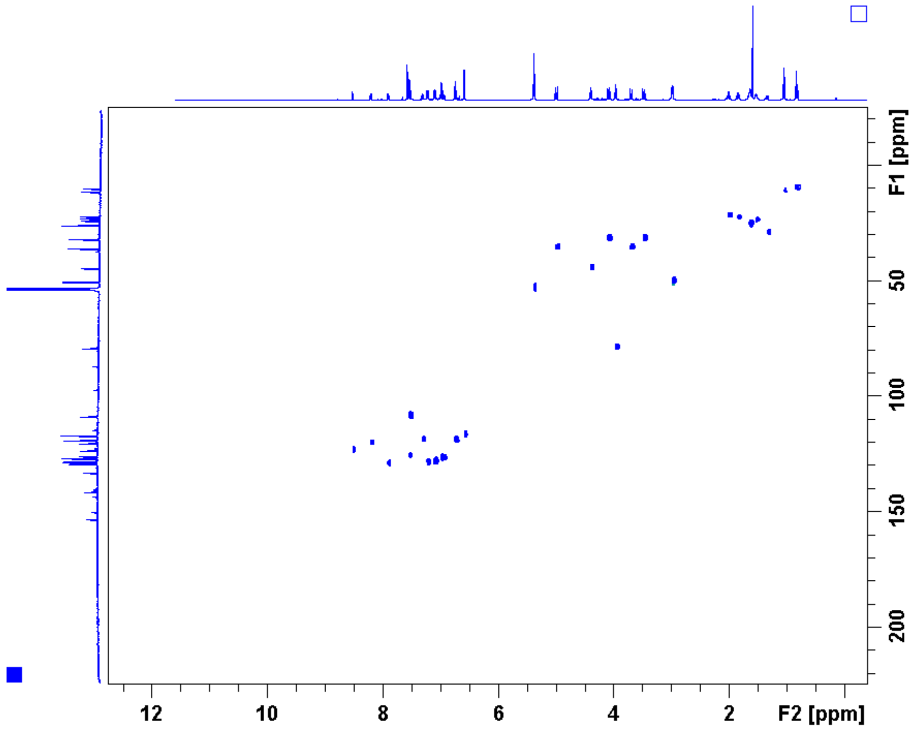

HSQC NMR of carbazole-appended calix[4]arene 3b

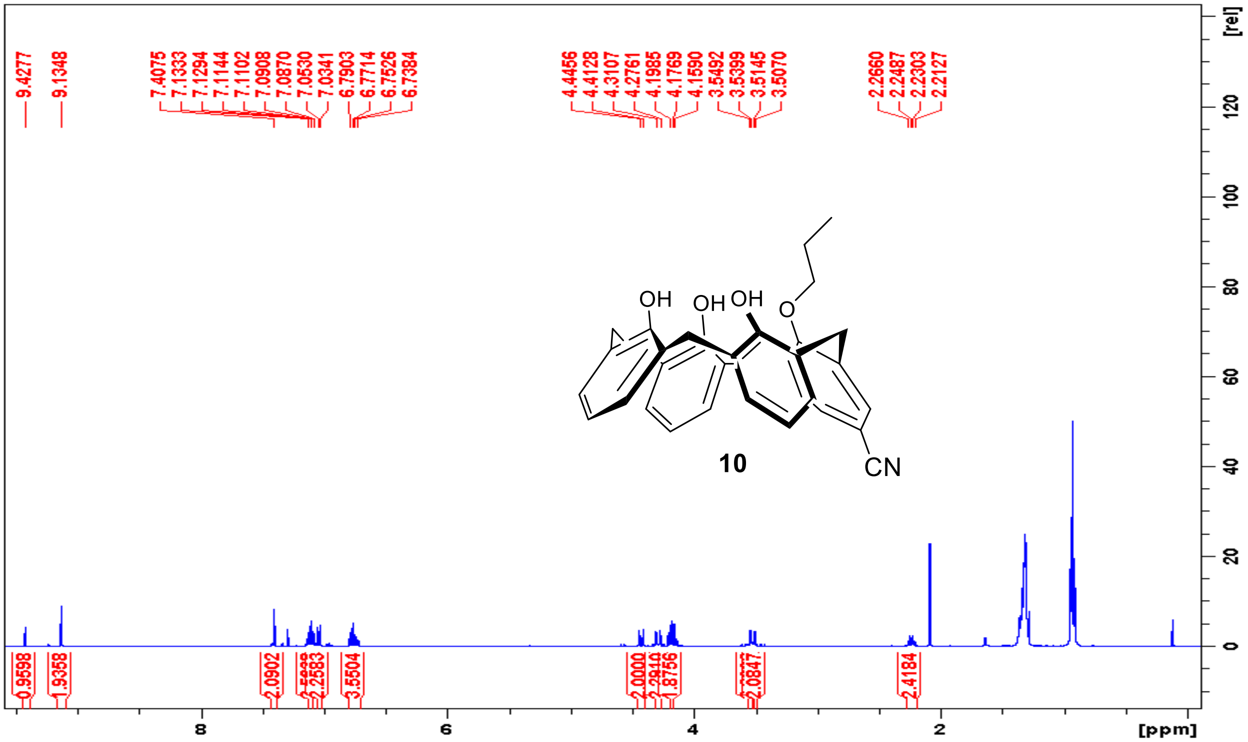

^{1}H NMR of 10

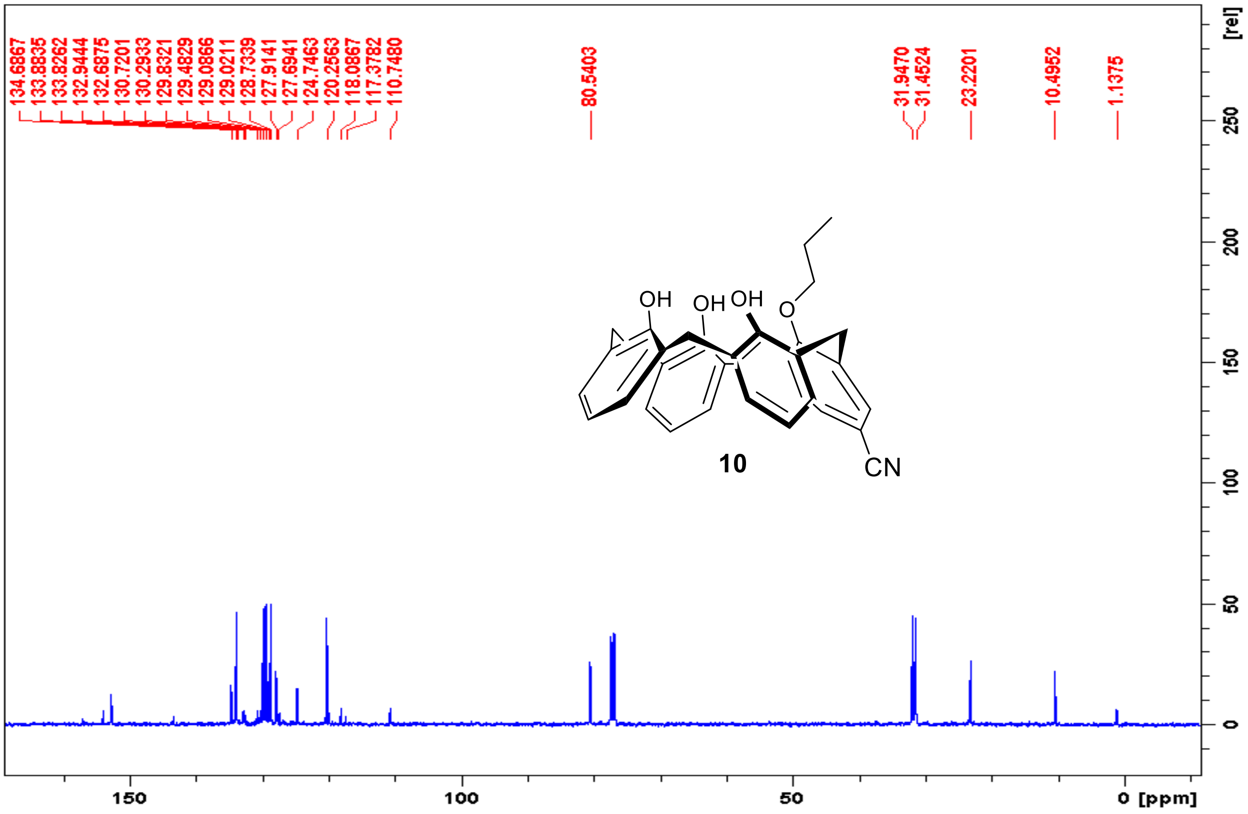

^{13}C NMR of 10

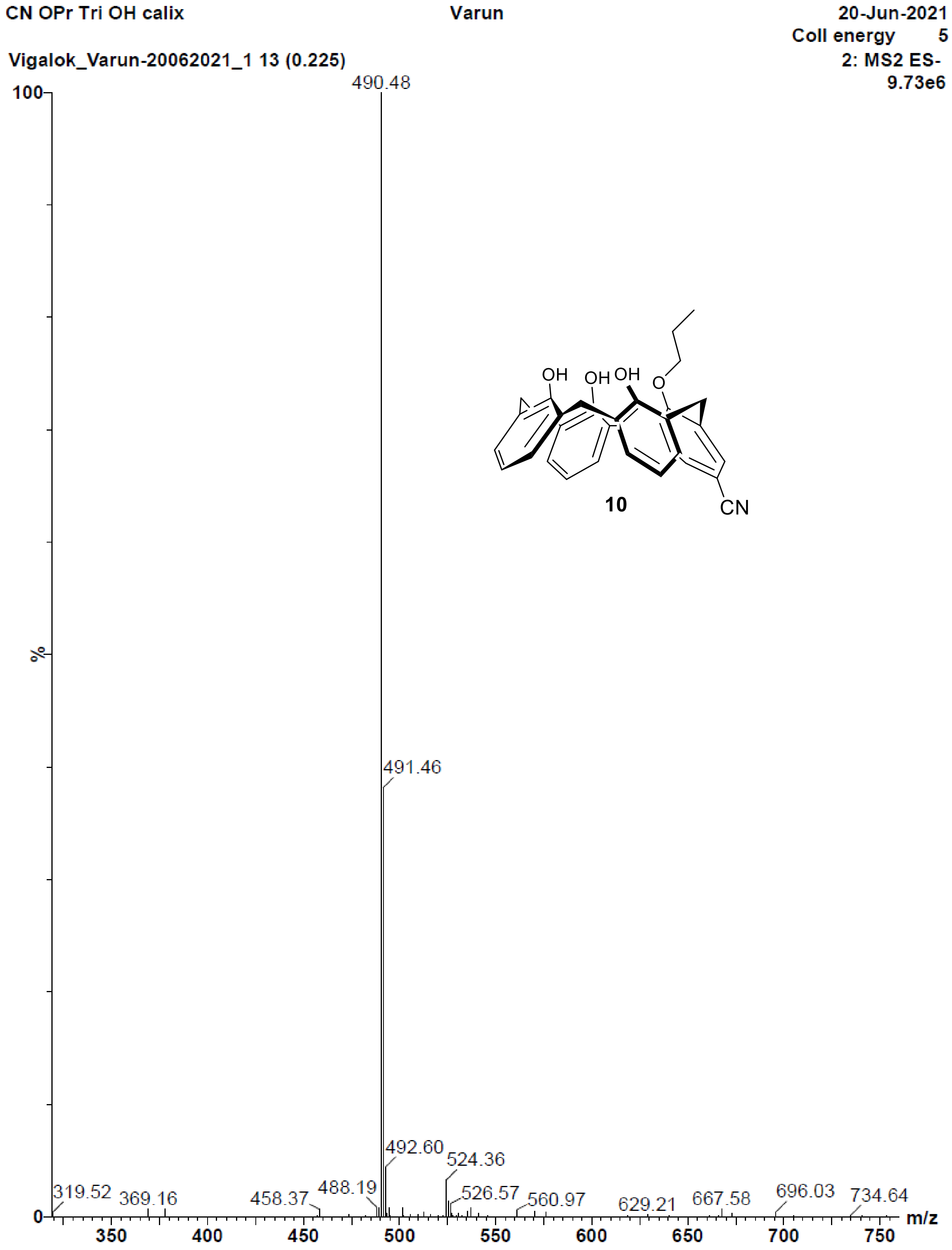
CN OPr Tri OH calix
Varun
20-Jun-2021
Coll energy 5
Vigalok_Varun-20062021_1 13 (0.225)
2: MS2 ES-
9.73e6
100
490.48
%
491.46
492.60
524.36
319.52
369.16
458.37
488.19
526.57
560.97
629.21
667.58
696.03
734.64
0
350
400
450
500
550
600
650
700
750
m/z
OH
OH
OH
O
10
CN

ESI MS of 10

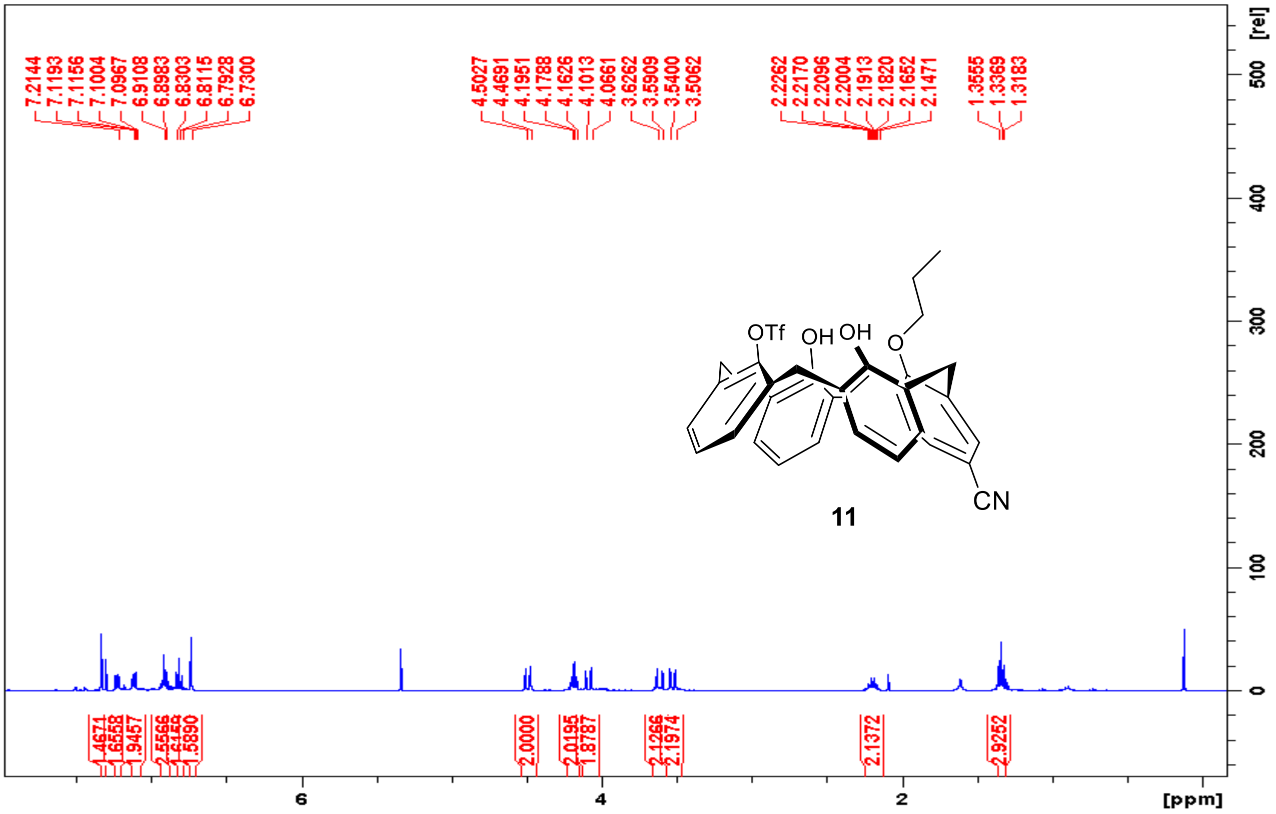

¹H NMR of 11

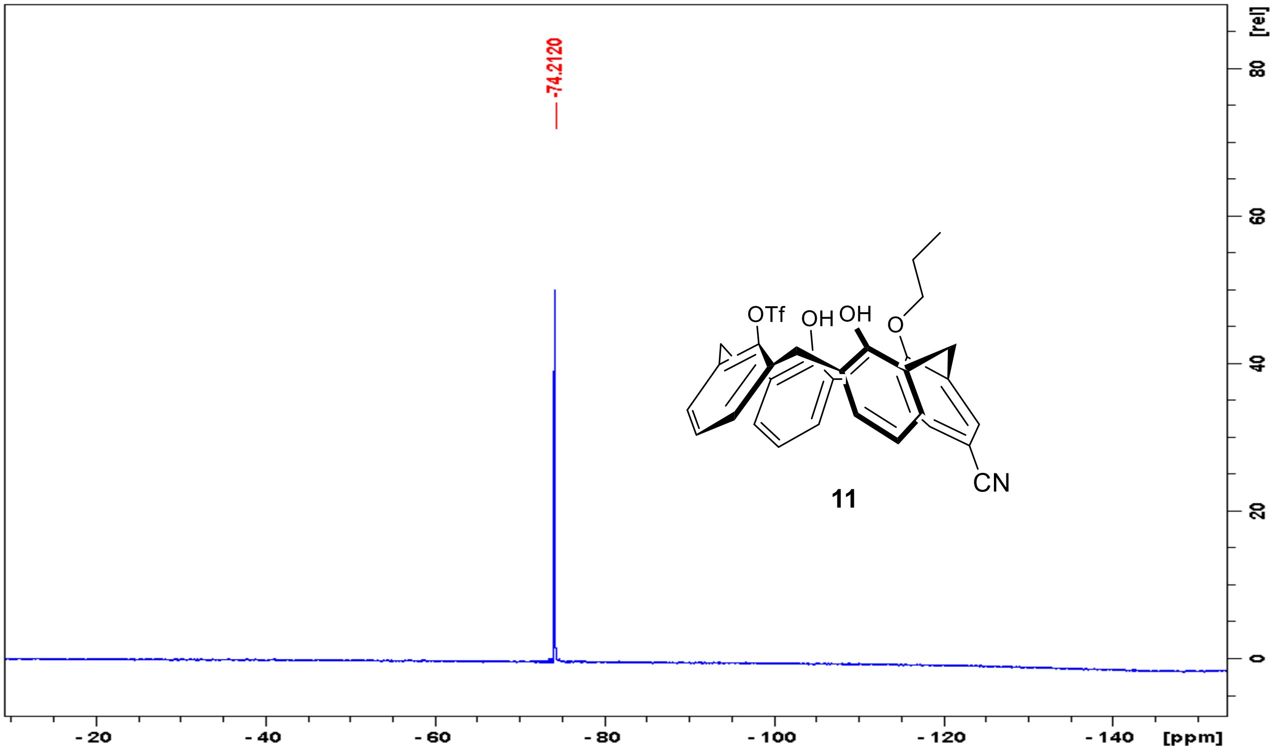

¹⁹F NMR of 11

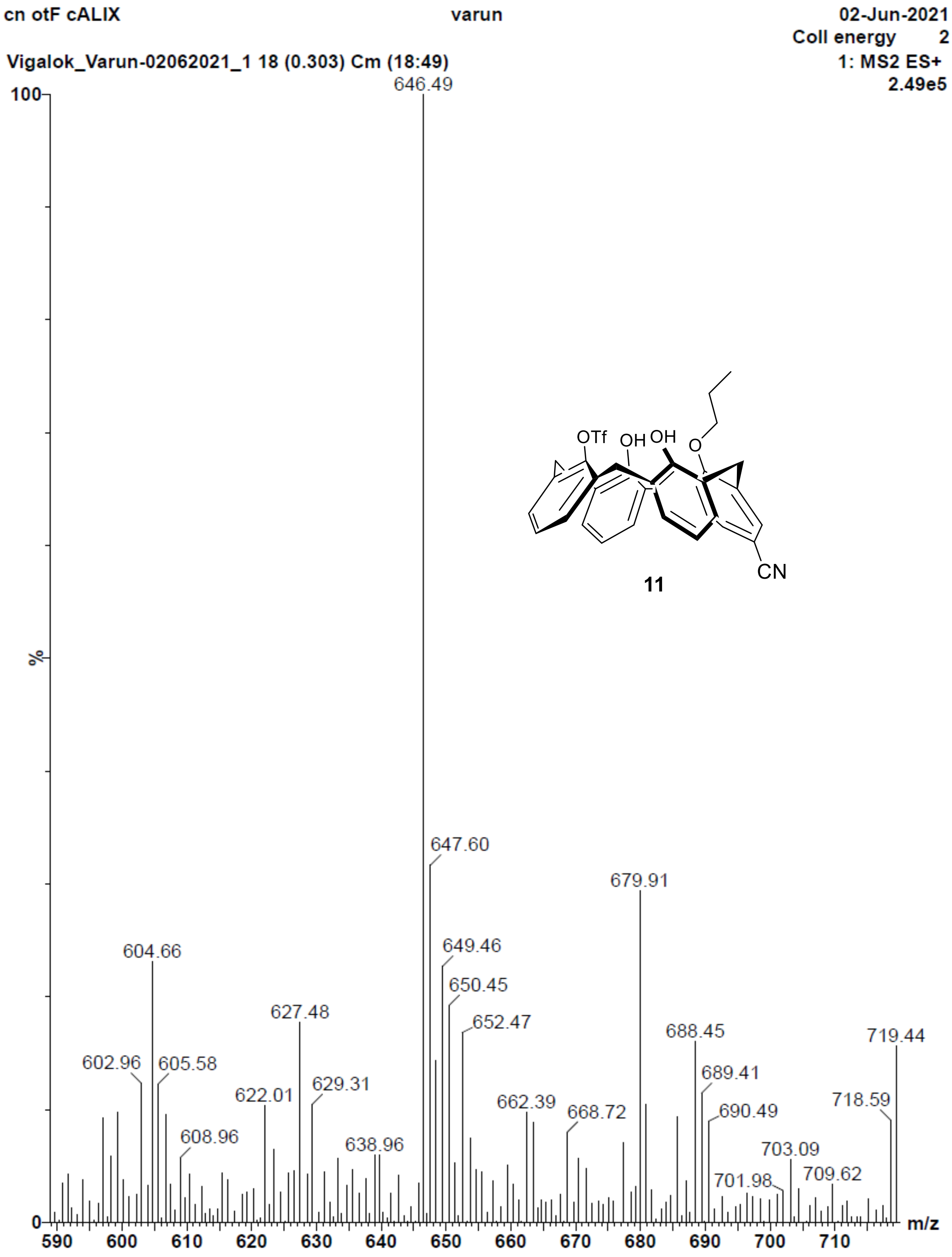
cn otF cALIX
varun
02-Jun-2021
Coll energy 2
Vigalok_Varun-02062021_1 18 (0.303) Cm (18:49)
1: MS2 ES+
2.49e5
100
%
0
646.49
647.60
679.91
604.66
649.46
650.45
627.48
652.47
688.45
719.44
602.96
605.58
689.41
622.01
629.31
662.39
718.59
668.72
690.49
608.96
638.96
703.09
701.98
709.62
590 600 610 620 630 640 650 660 670 680 690 700 710
m/z
OTf
OH
OH
O
CN
11

ESI MS of 11

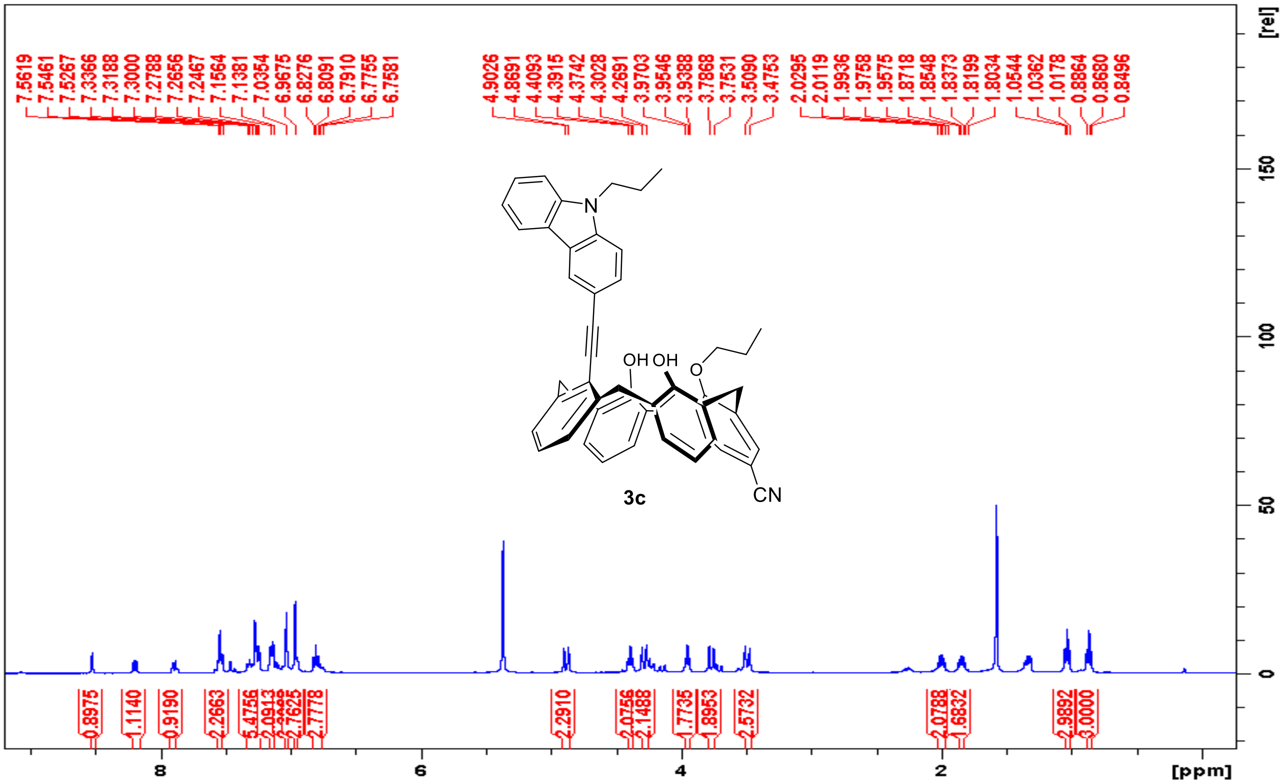

^{1}H NMR of carbazole-appended calix[4]arene 3c

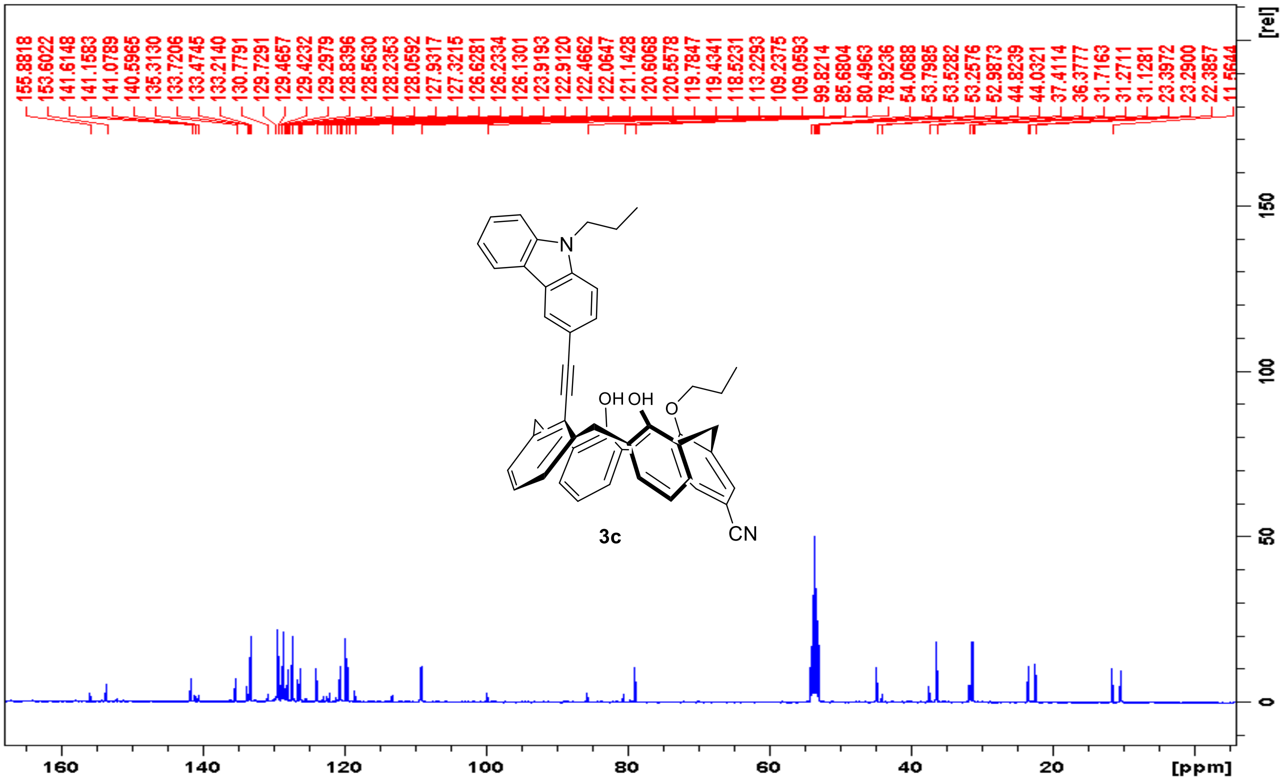

^{13}C NMR of carbazole-appended calix[4]arene 3c

Single Mass Analysis
Tolerance = 10.0 PPM / DBE: min = 0.0, max = 50.0
Element prediction: Off
Number of isotope peaks used for i-FIT = 3
Monoisotopic Mass, Even Electron Ions
90 formula(e) evaluated with 3 results within limits (all results (up to 1000) for each mass)
Elements Used:

Mass	Calc. Mass	mDa	PPM	DBE	Formula	i-FIT	i-FIT Norm	Fit Conf %	C	H	N	O
707.3279	707.3274	0.5	0.7	29.5	C49 H43 N2 O3	954.2	1.900	14.96	49	43	2	3
	707.3314	-3.5	-4.9	33.5	C54 H43 O	957.3	5.019	0.66	54	43		1
	707.3233	4.6	6.5	25.5	C44 H43 N4 O5	952.5	0.170	84.38	44	43	4	5

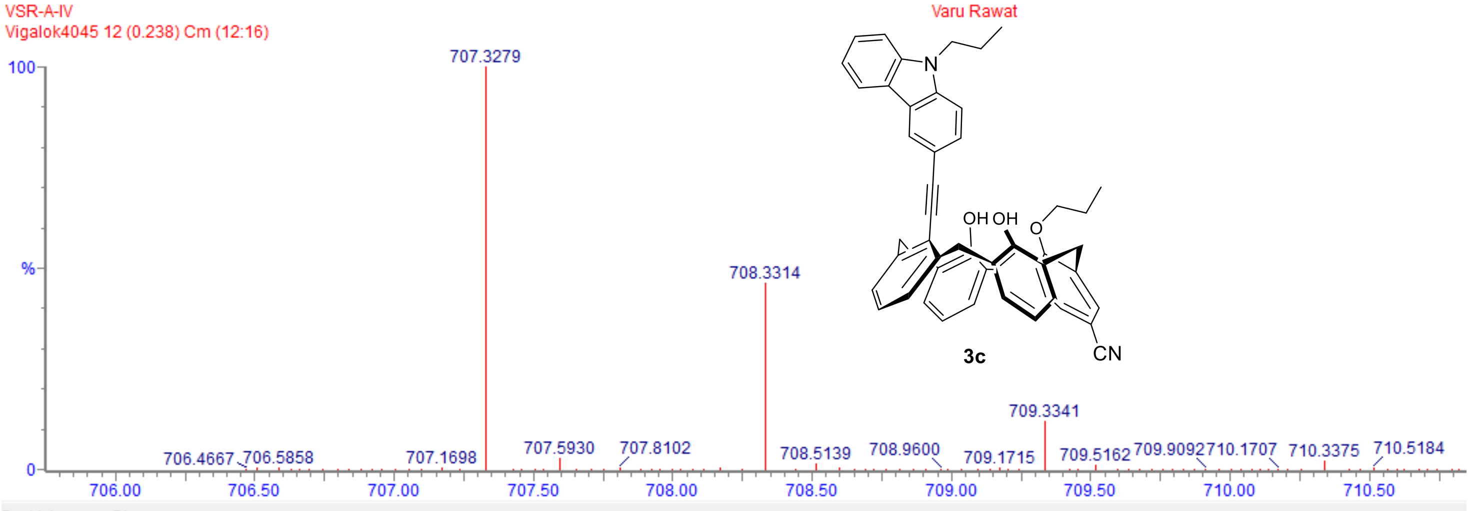

HRMS of carbazole-appended calix[4]arene 3c

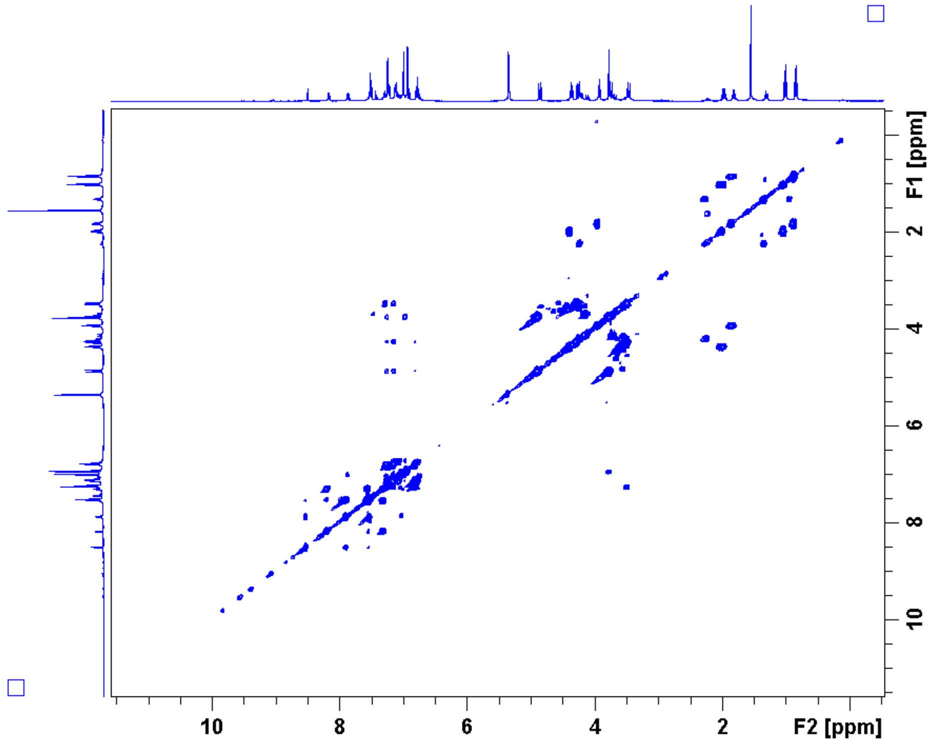

COSY NMR of carbazole-appended calix[4]arene 3c

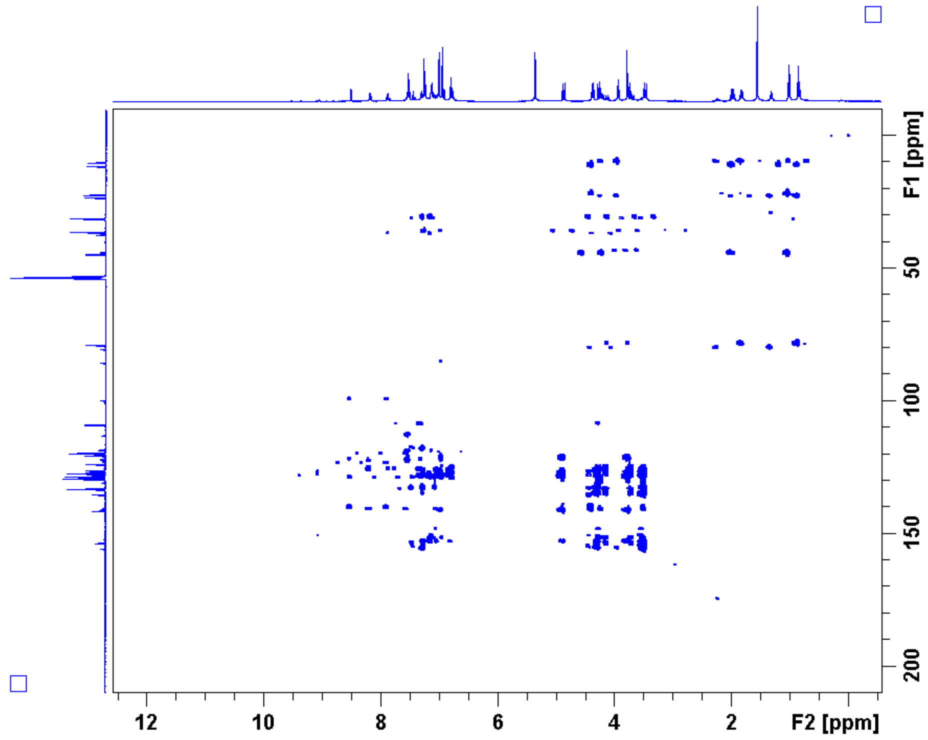

HMBC NMR of carbazole-appended calix[4]arene 3c

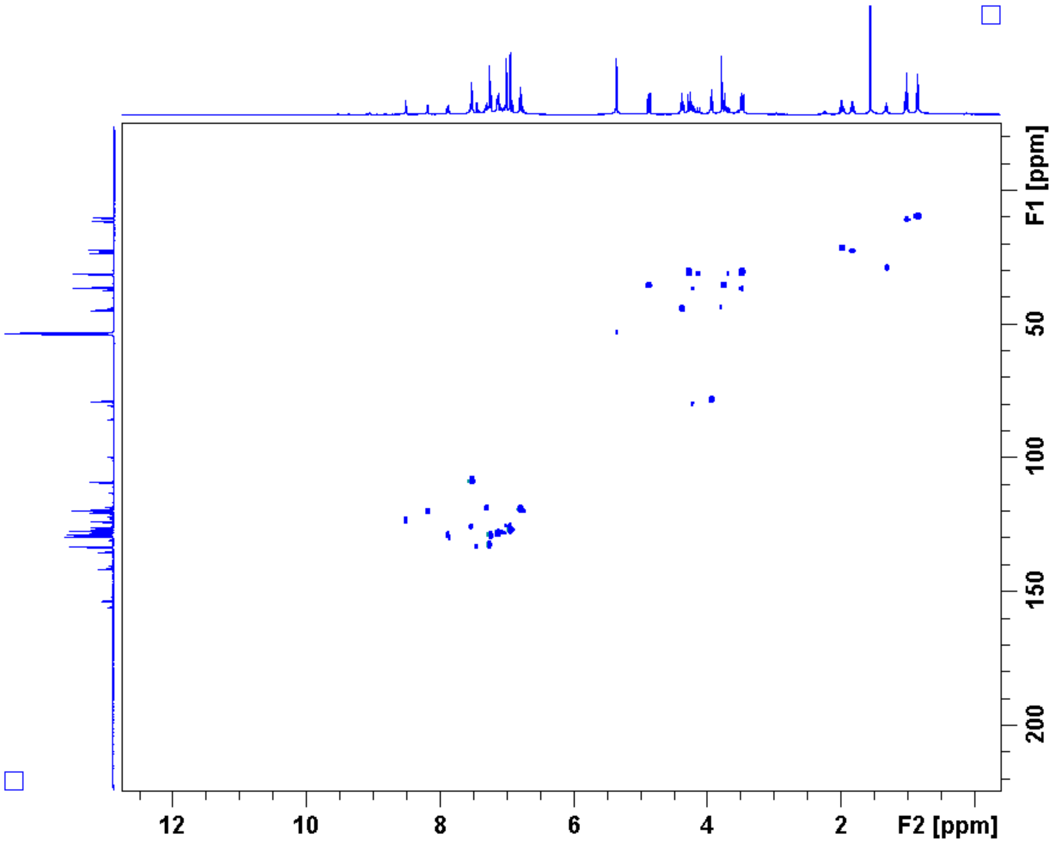

HSQC NMR of carbazole-appended calix[4]arene 3c

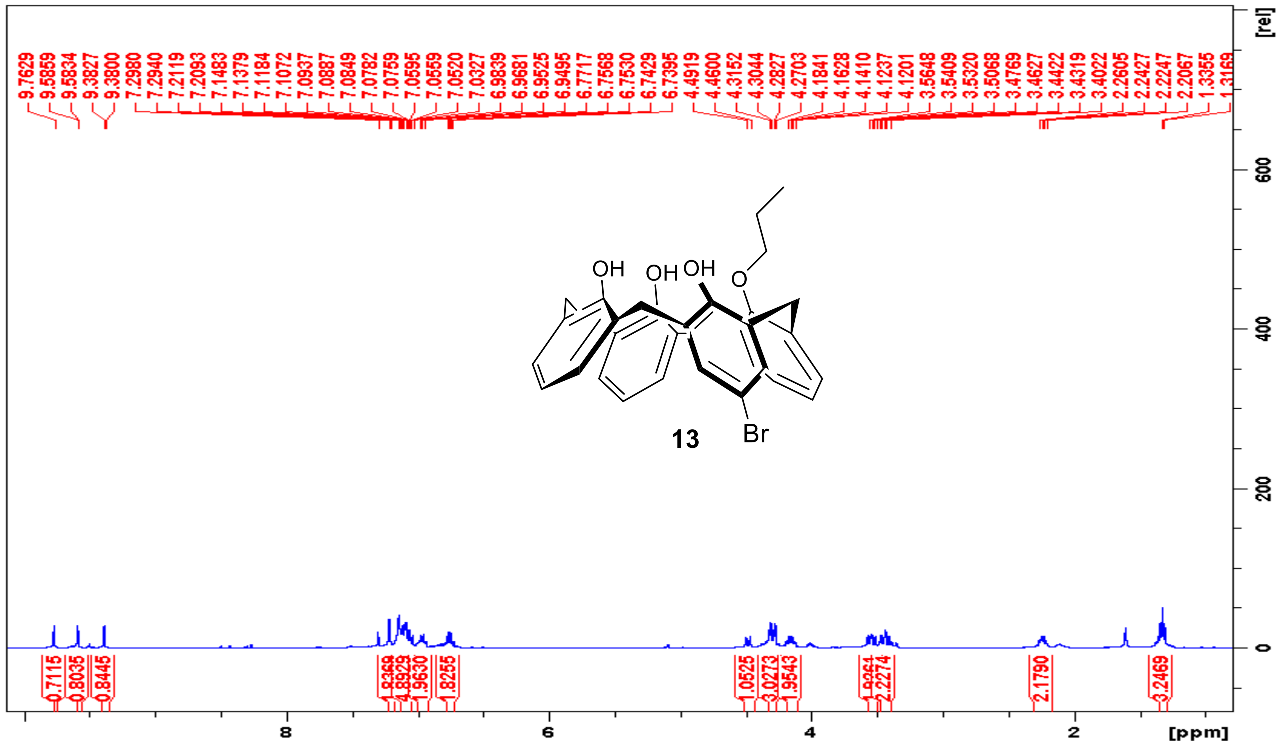

^{1}H NMR of 13

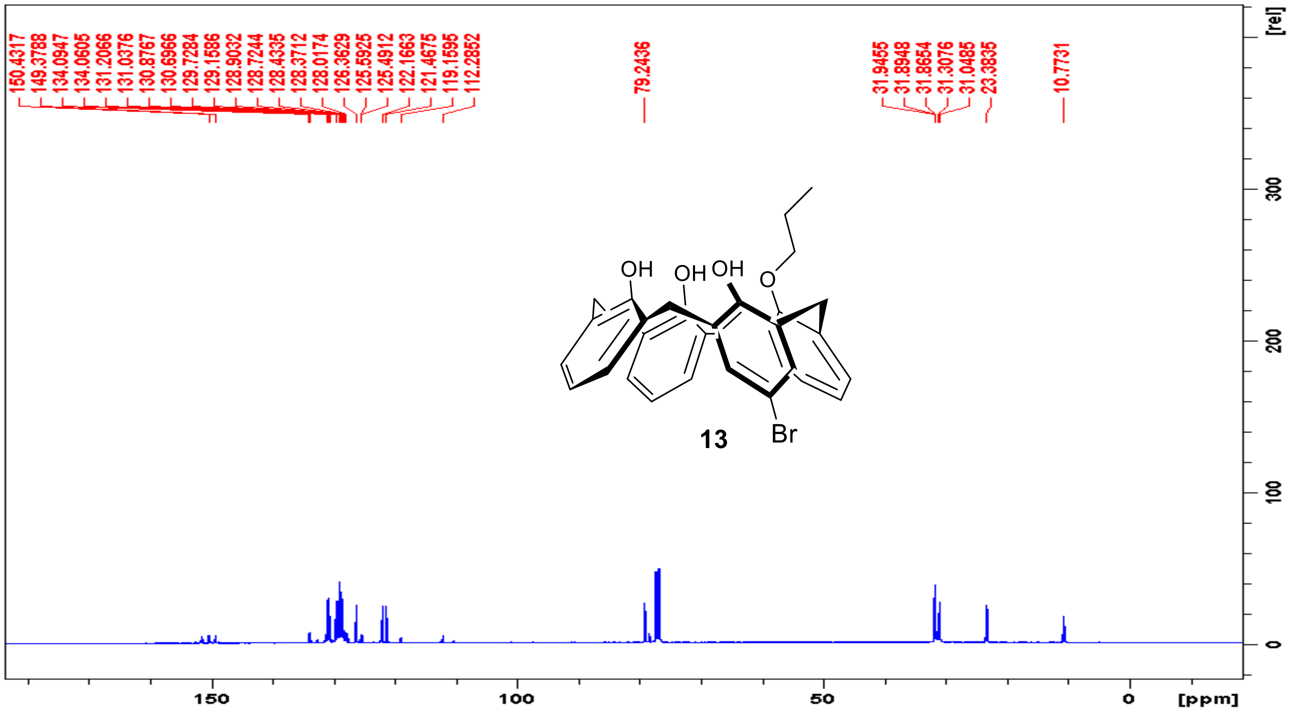

^{13}C NMR of 13

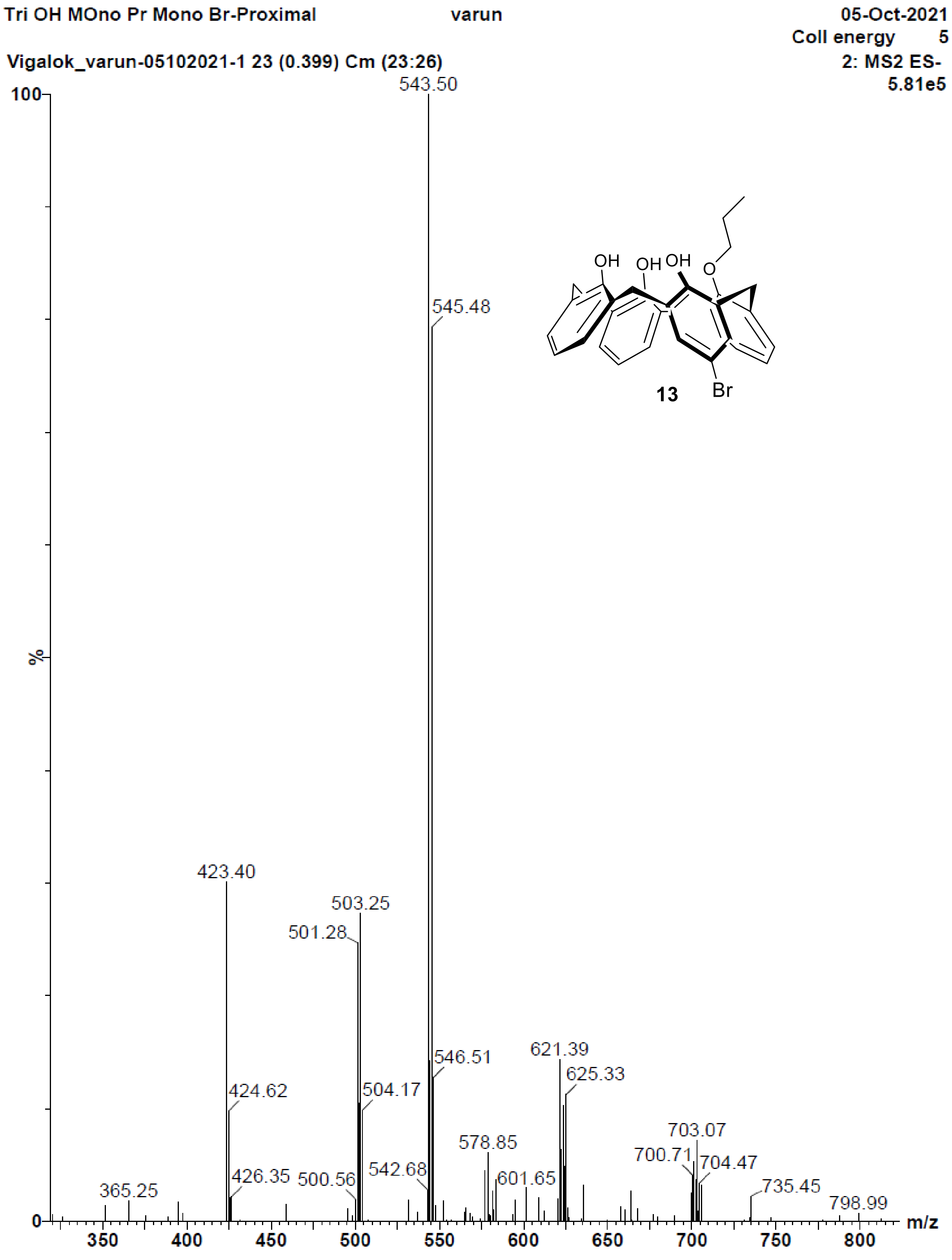
Tri OH MOno Pr Mono Br-Proximal
varun
05-Oct-2021
Coll energy 5
Vigalok_varun-05102021-1 23 (0.399) Cm (23:26)
2: MS2 ES-
5.81e5
100
%
0
543.50
545.48
423.40
503.25
501.28
621.39
625.33
546.51
504.17
424.62
703.07
700.71
704.47
578.85
735.45
798.99
365.25
426.35
500.56
542.68
601.65
350
400
450
500
550
600
650
700
750
800
m/z
OH
OH
OH
O
Br
13

ESI MS of 13

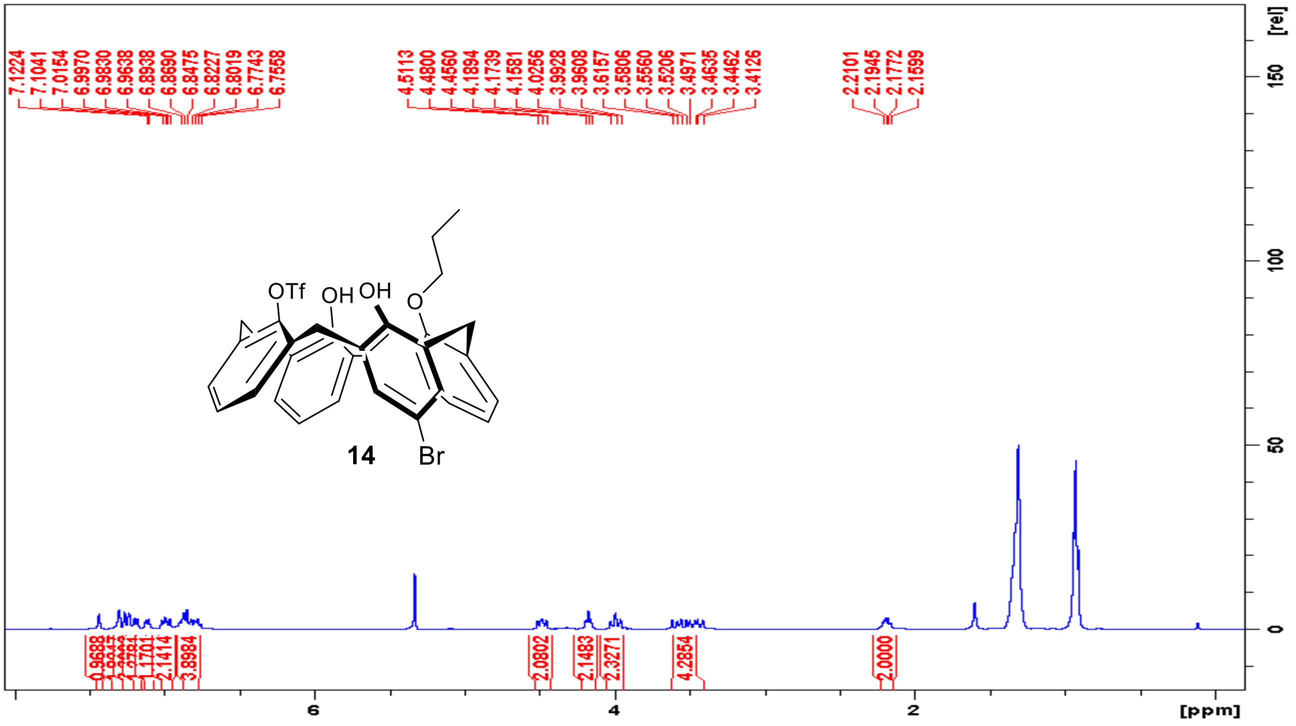

^{1}H NMR of 14

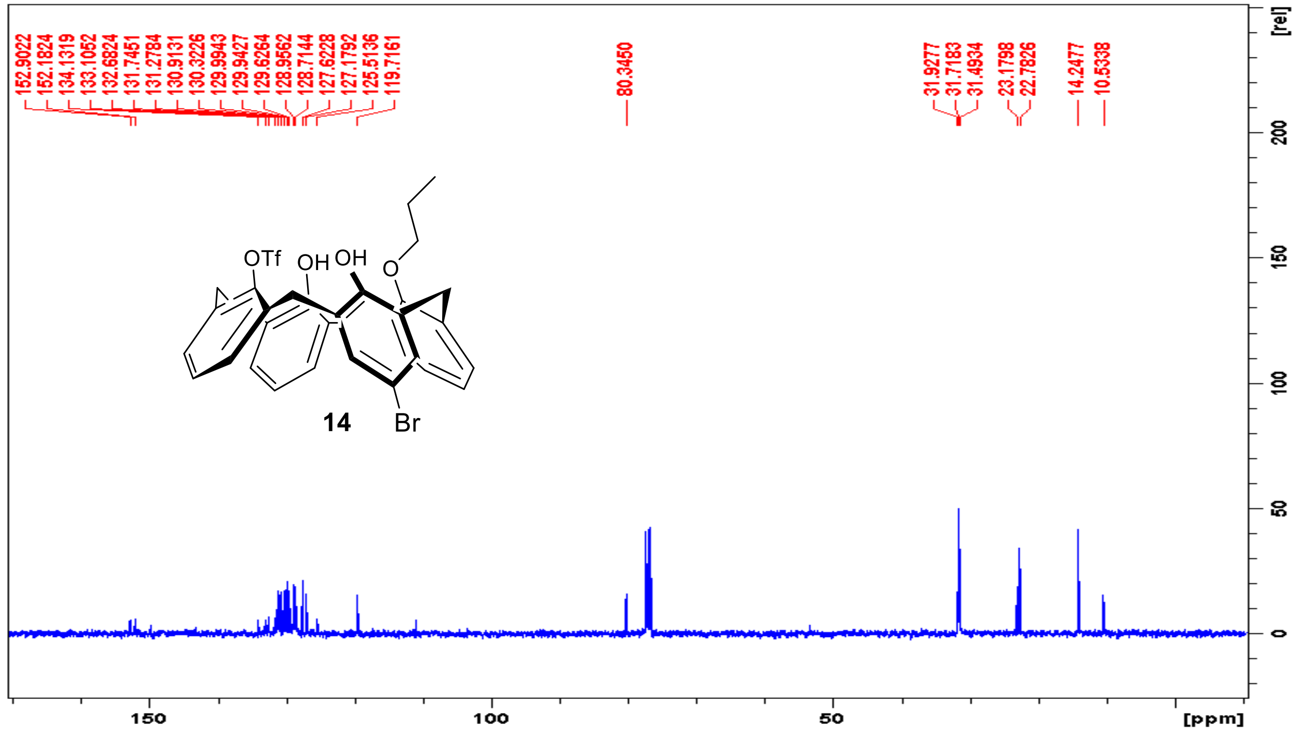

^{13}C NMR of 14

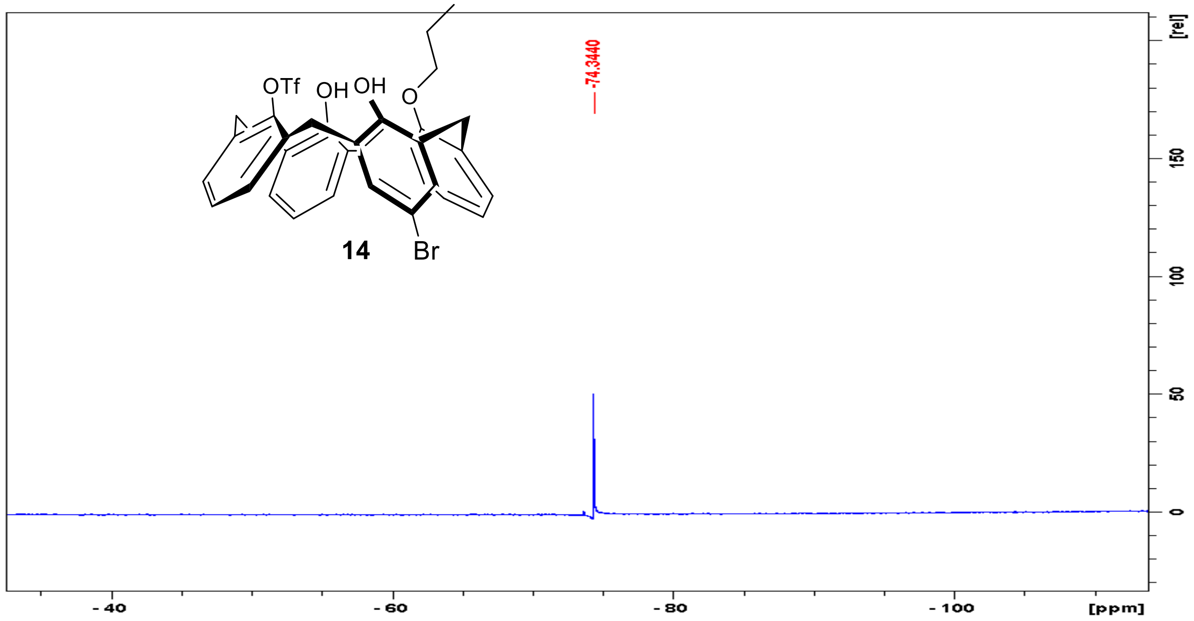

^{19}F NMR of 14

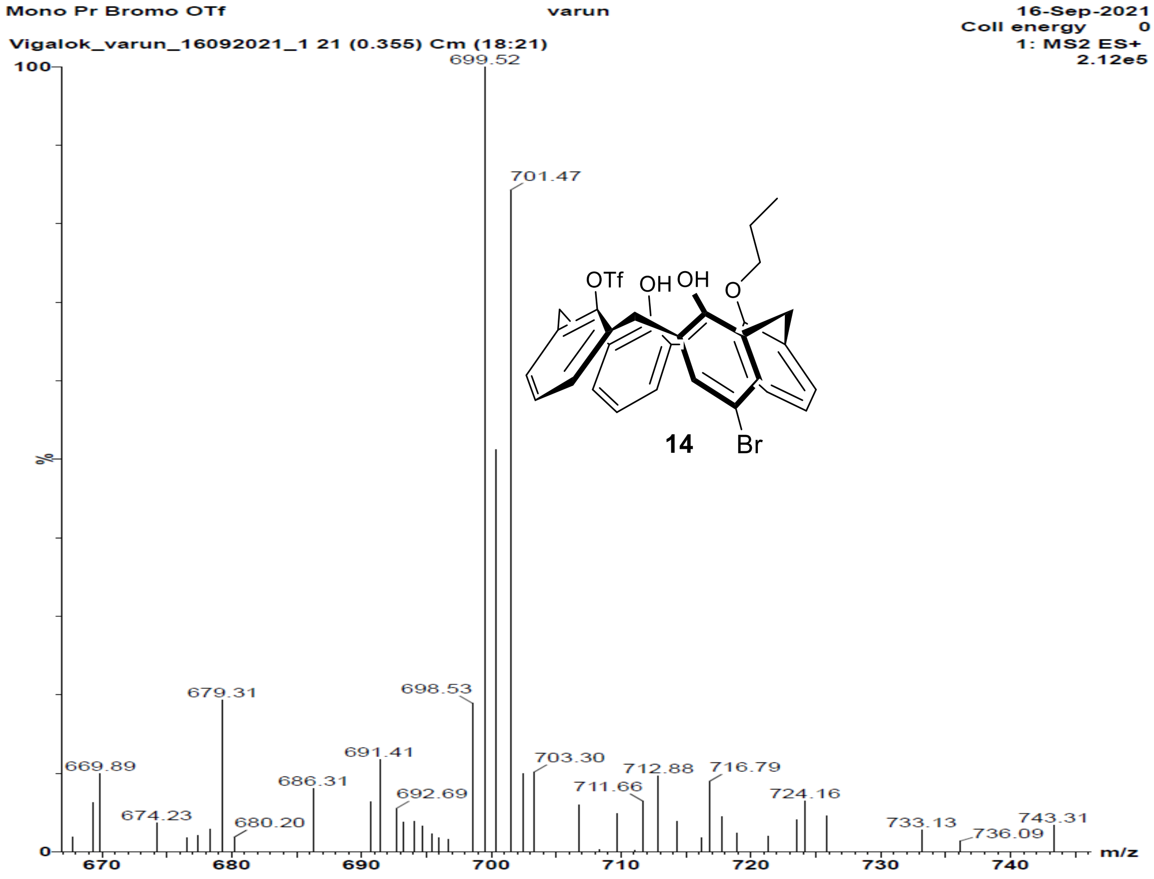

ESI MS of 14

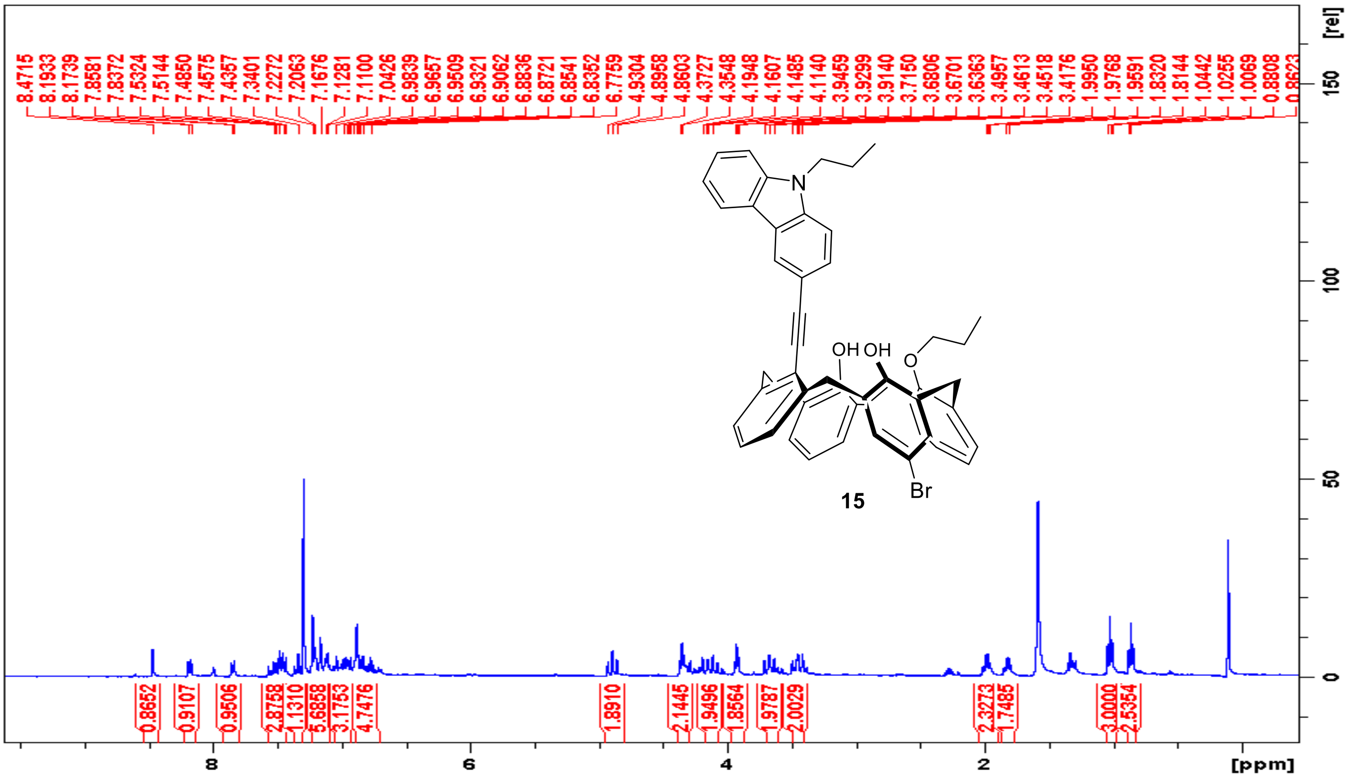

^{1}H NMR of 15

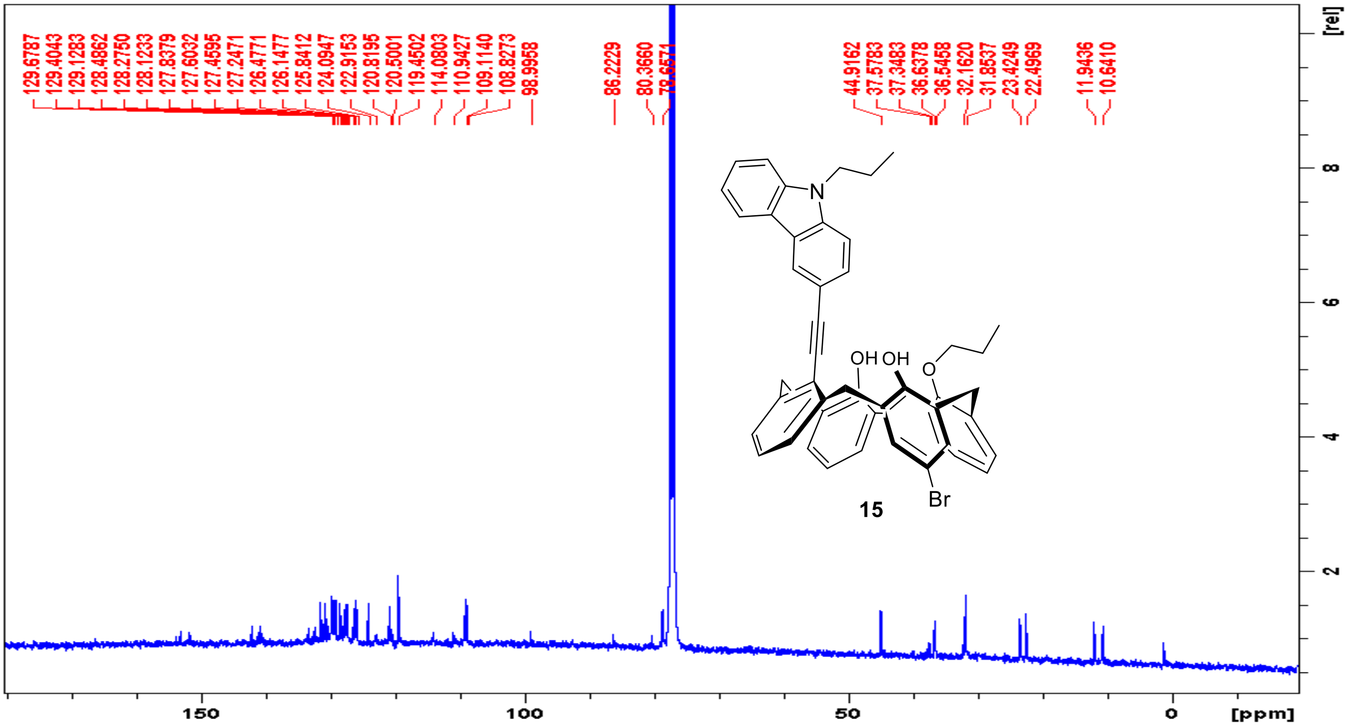

^{13}C NMR of 15

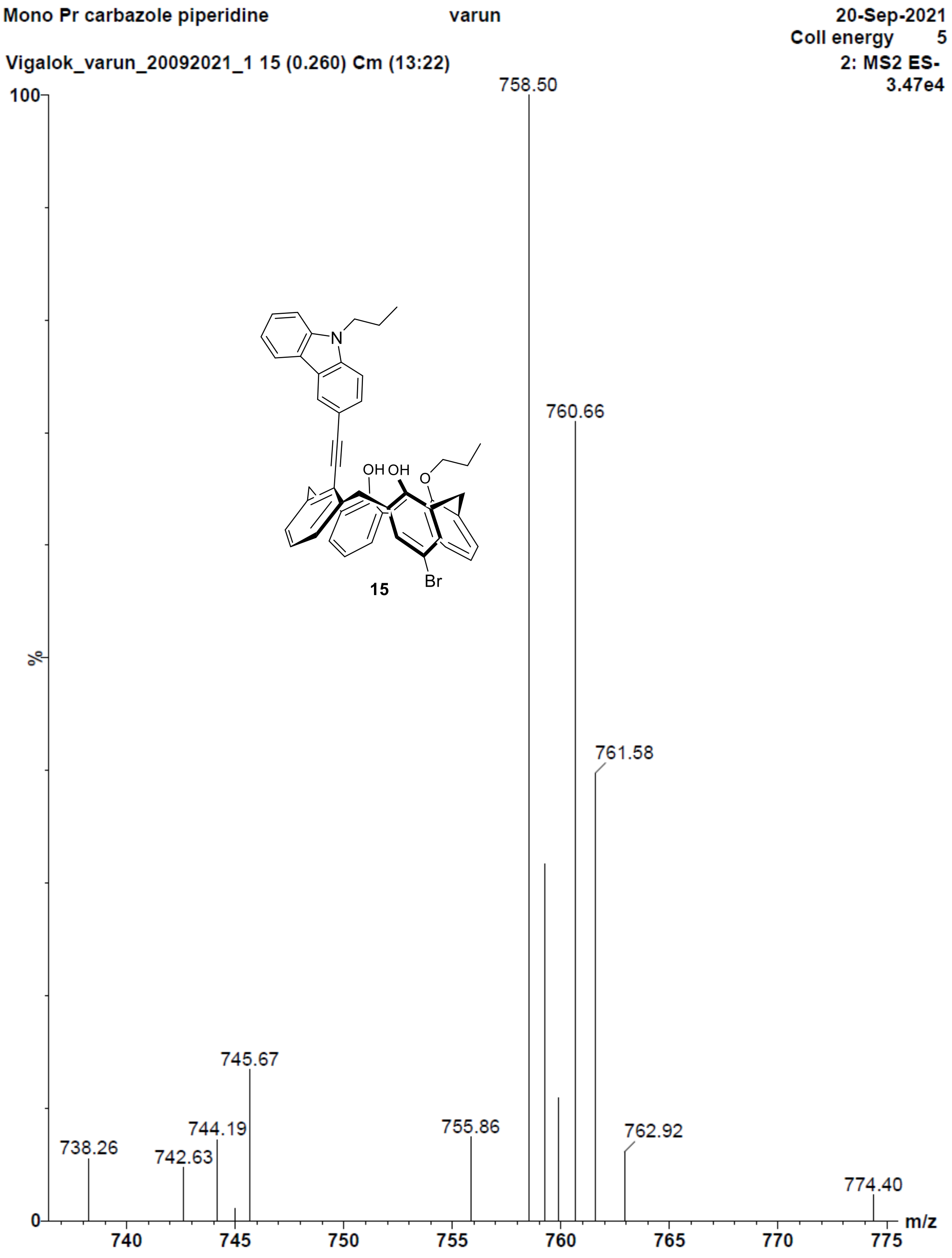
Mono Pr carbazole piperidine
varun
20-Sep-2021
Coll energy 5
Vigalok_varun_20092021_1 15 (0.260) Cm (13:22)
2: MS2 ES-
3.47e4
100
%
0
758.50
760.66
761.58
745.67
744.19
738.26
742.63
755.86
762.92
774.40
740
745
750
755
760
765
770
775
m/z
N
OH
OH
O
Br
15

ESI MS of 15

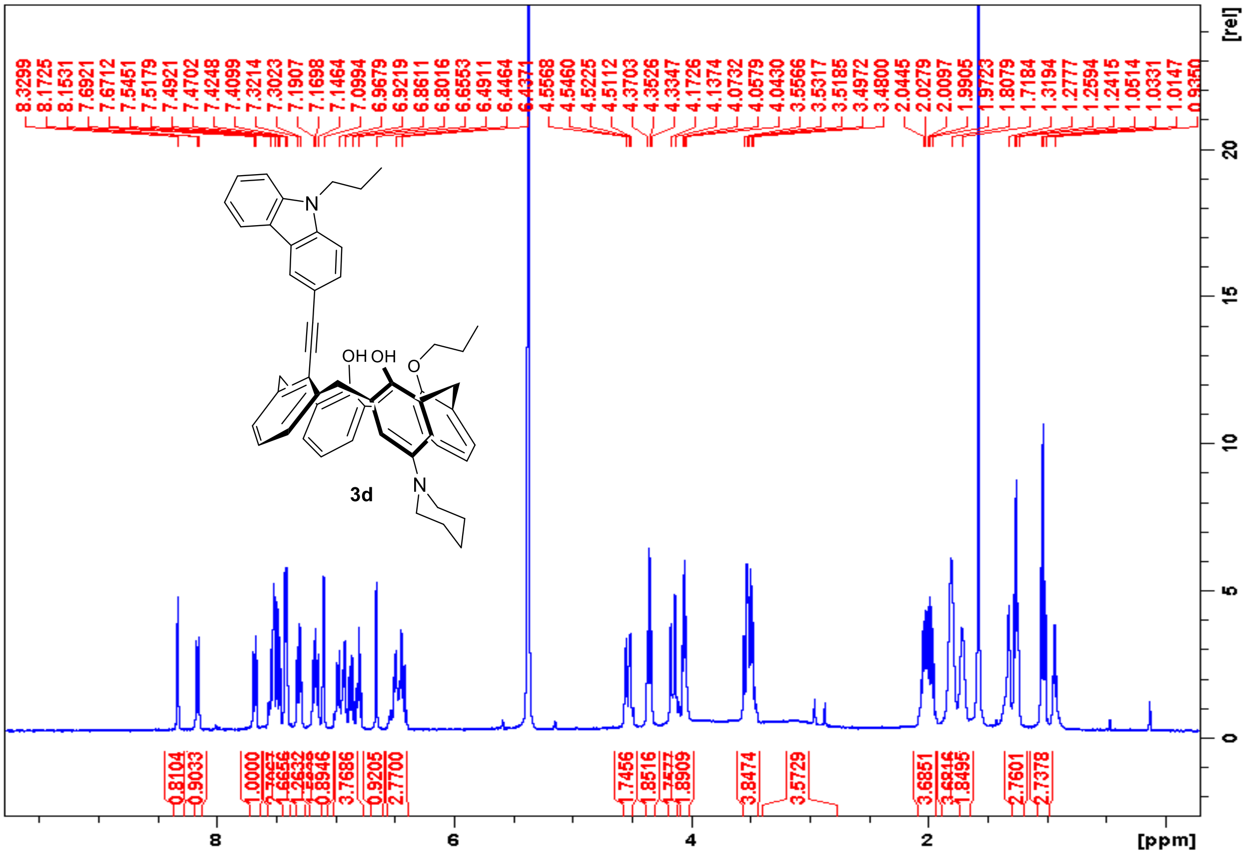

^{1}H NMR of carbazole-appended calix[4]arene 3d

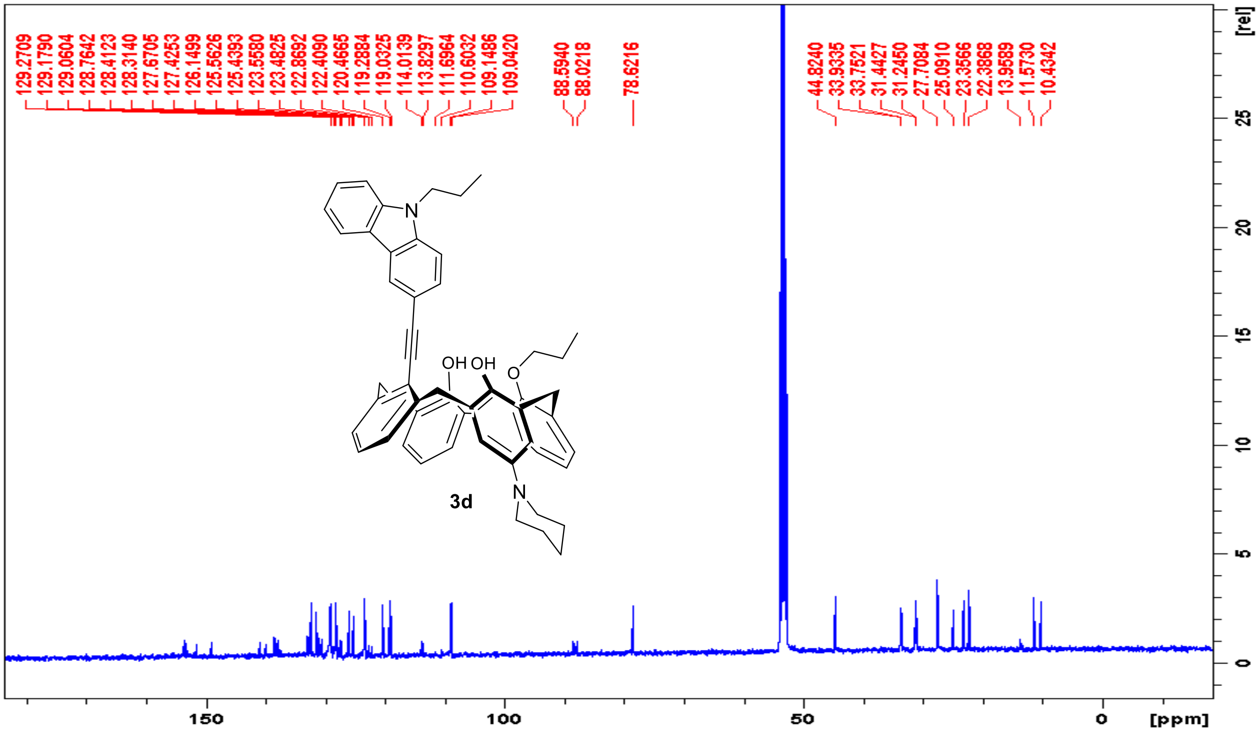

^{13}C NMR of carbazole-appended calix[4]arene 3d

Single Mass Analysis
Tolerance = 10.0 PPM / DBE: min = 0.0, max = 50.0
Element prediction: Off
Number of isotope peaks used for i-FIT = 3
Monoisotopic Mass, Even Electron Ions
89 formula(e) evaluated with 3 results within limits (all results (up to 1000) for each mass)
Elements Used:

Mass	Calc. Mass	mDa	PPM	DBE	Formula	i-FIT	i-FIT Norm	Fit Conf %	C	H	N	O
765.4057	765.4056	0.1	0.1	28.5	C53 H53 N2 O3	1091.0	4.196	1.51	53	53	2	3
	765.4096	-3.9	-5.1	32.5	C58 H53 O	1095.0	8.271	0.03	58	53		1
	765.4016	4.1	5.4	24.5	C48 H53 N4 O5	1086.8	0.015	98.47	48	53	4	5

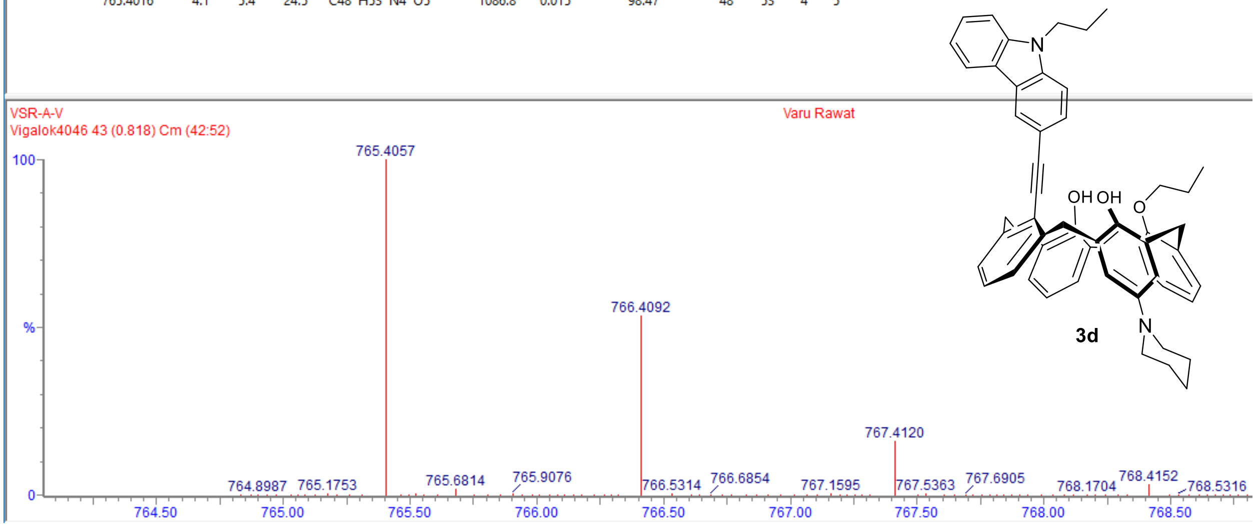

HRMS of carbazole-appended calix[4]arene 3d

3. Absorption and Emission Spectra

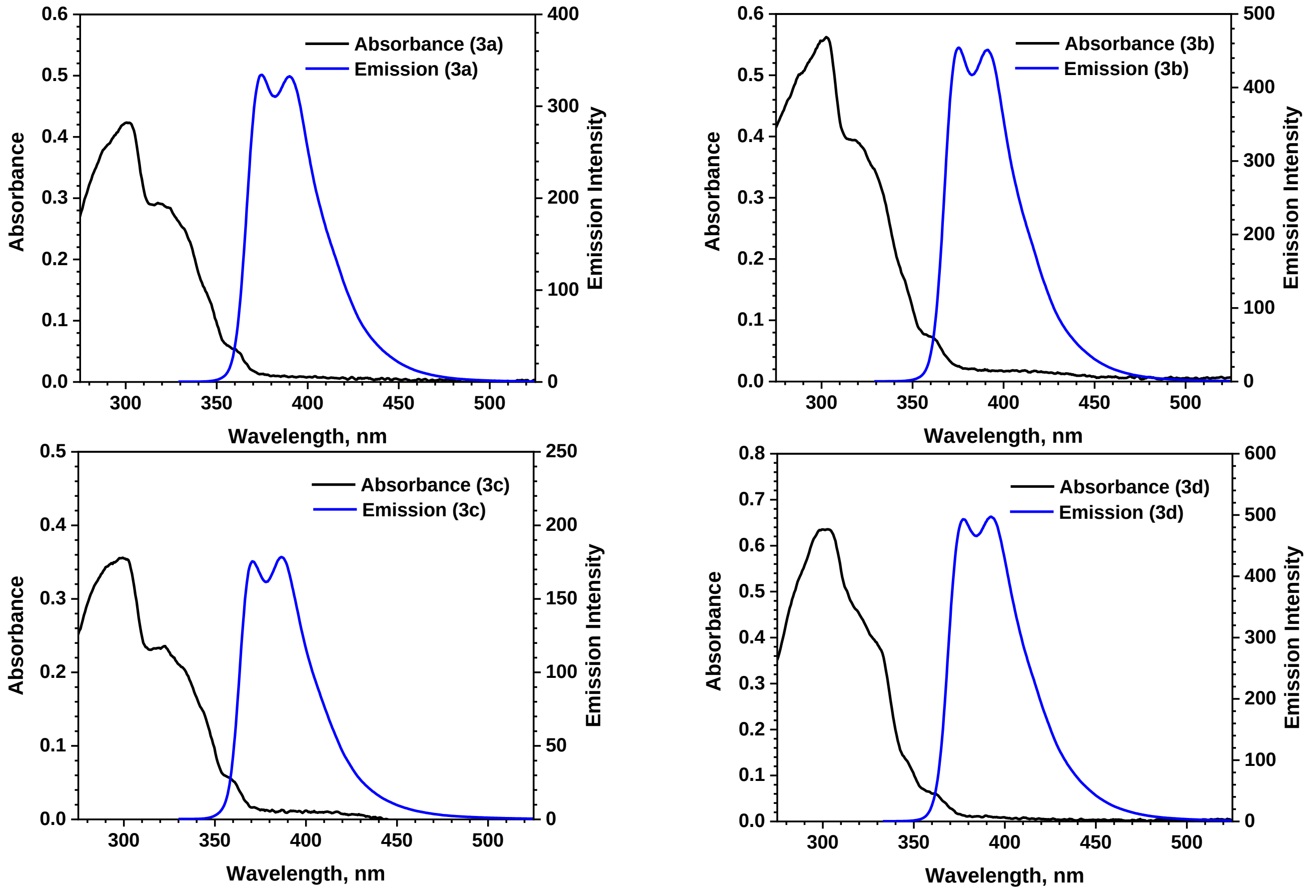

Figure S1: Absorption and Emission spectra of carbazole-appended calixarenes (**3a-d**) recorded in DCE (10 μM).

4. Host-guest Complexation Studies

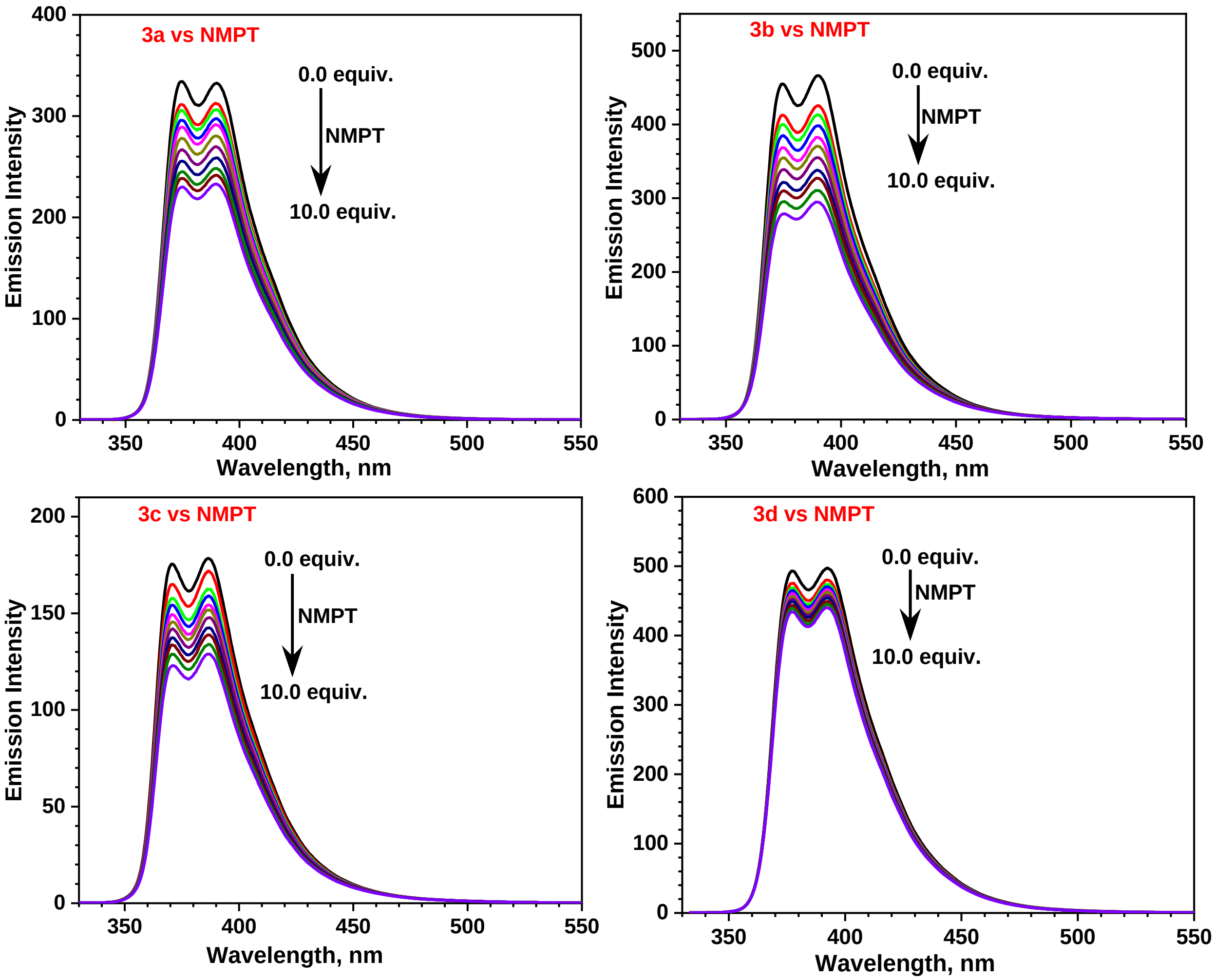

Figure S2: Fluorescent spectra of carbazole-appended calixarenes **3a-d** (DCE, 10 mM) with various concentrations of NMPT, **2** (0 to 10 equivalents).

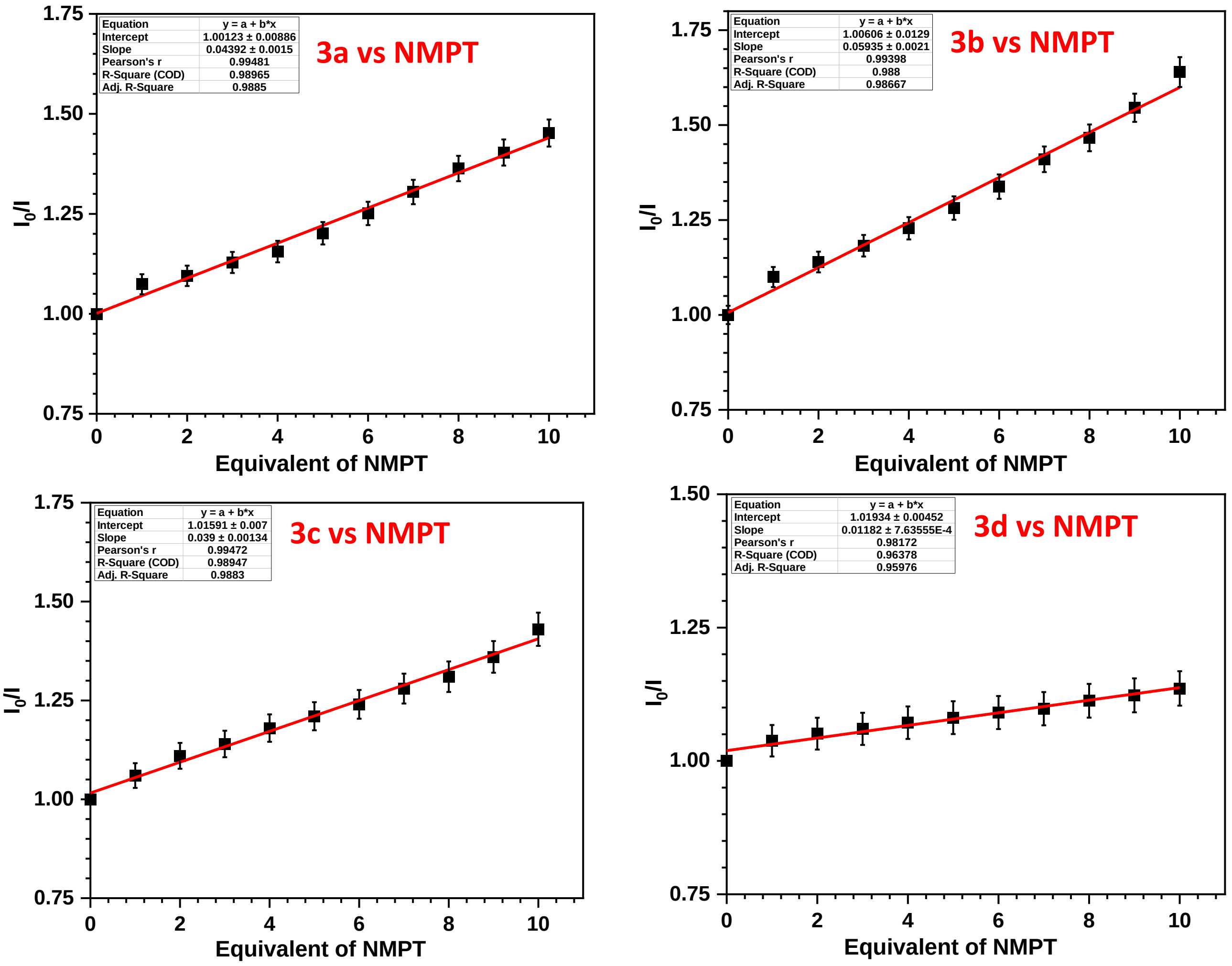

Figure S3: Stern-Volmer plot (I_o/I) of **3a-d** vs increasing equivalents of NMPT

Table S1: Emission intensity dependence on the concentration of **2**

Calixarene	% Decrease in Emission Intensity			
	1 equiv. 2	5 equiv. 2	10 equiv. 2	K_{ass}
**3a**	7%	17%	33%	4392 ±150 M^{-1}
**3b**	9%	25%	39%	5935 ±210 M^{-1}
**3c**	6%	17%	30%	3900 ±134 M^{-1}
**3d**	4%	8%	12%	1182 ±76 M^{-1}

Conditions: Titration was done with 10 μM solution of calixarenes (**3a-d**) in DCE with increasing concentration of NMPT (0 to 10 equivalents). Linear fitting eqn. $I_o/I = 1 + K_{ass}Q$.

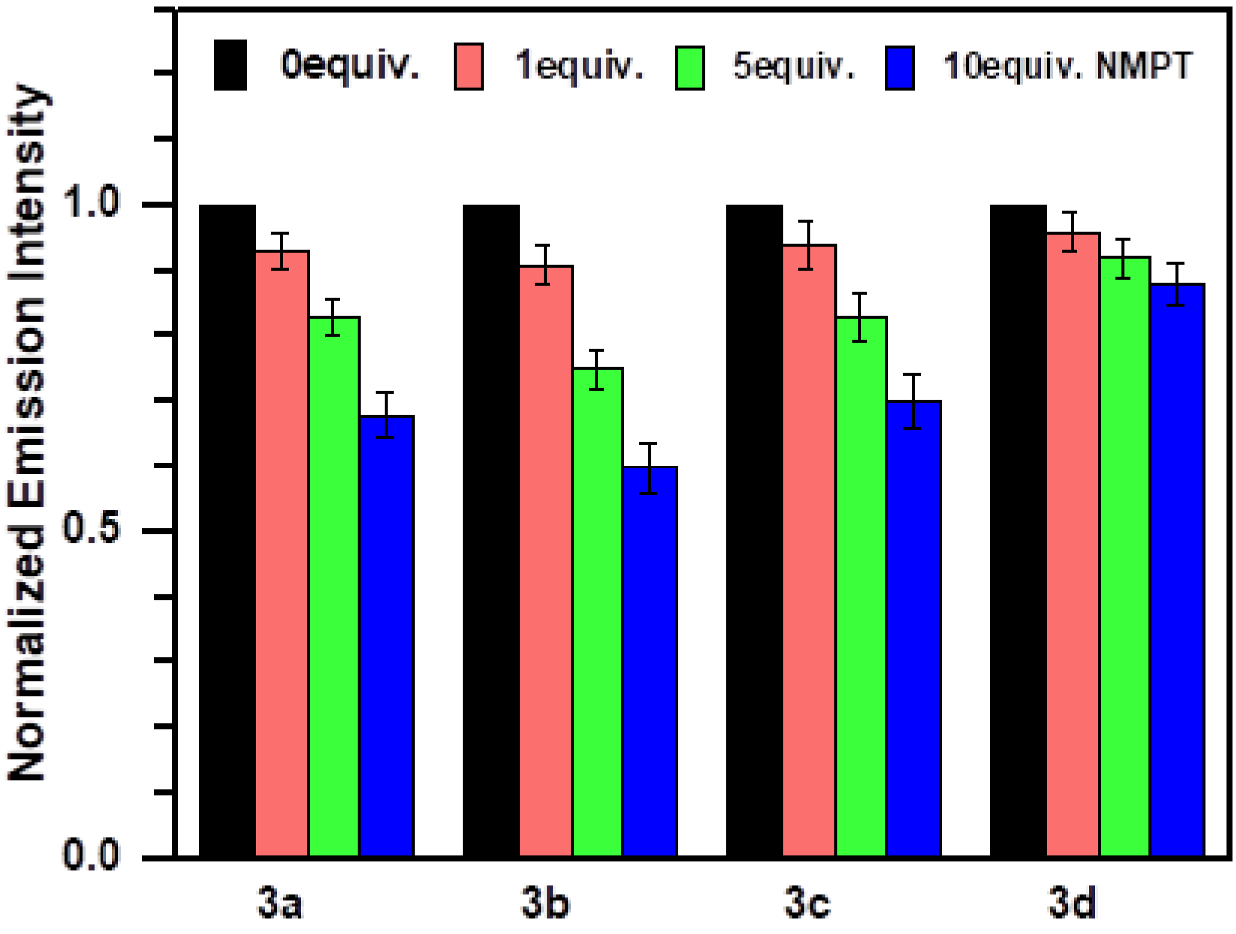

Figure S4: Fluorescence quenching in carbazole-appended calixarenes **3a-d** (DCE, 10 mM) upon the addition of N-methylpyridinium triflate (NMPT, **2**).

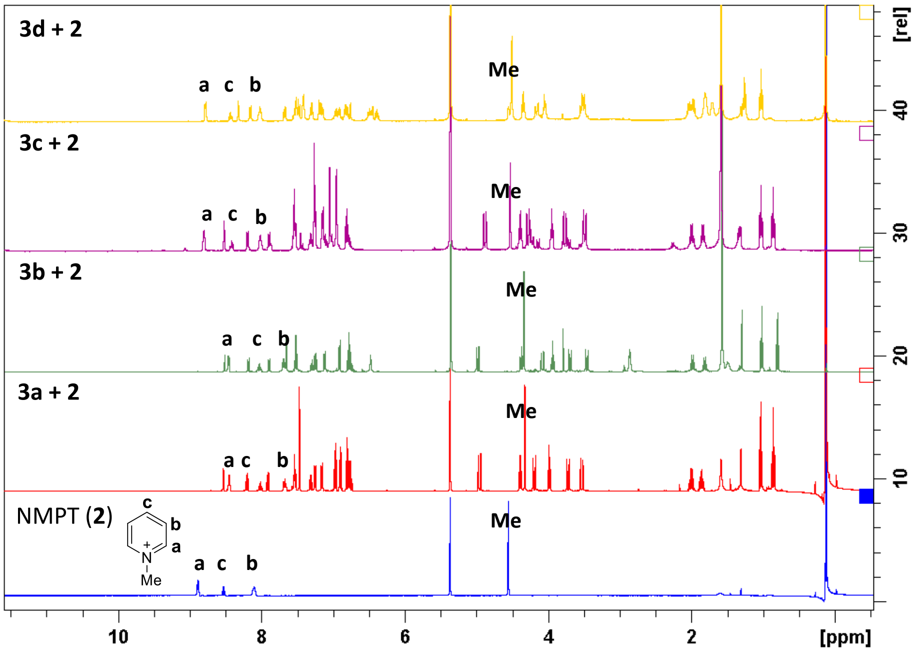

Figure S5: ^{1}H NMR (400 MHz) host-guest complexation studies in CD_2Cl_2 (5 mM solution).

Table S2: Comparison of ^{1}H NMR (400 MHz) chemical shifts during host-guest complexation

Resonance Signal	**2** δ, ppm	**3a + 2** δ, ppm	**3b + 2** δ, ppm	**3c + 2** δ, ppm	**3d + 2** δ, ppm
Me	4.56	4.33	4.36	4.53	4.51
H_a	8.88	8.45	8.47	8.80	8.78
H_b	8.11	7.68	7.71	8.02	8.02
H_c	8.54	8.02	8.05	8.42	8.44

Conditions: Host-guest complexation studies were carried out in CD_2Cl_2 (5 mM soln).

5. Binding constant from NMR titration

In a typical ^{1}H NMR titration experiment, increasing amounts of a solution of the carbazole-appended calix[4]arene **3c** (usually c = 10^{-3} M) were added to a solution of the NMPT salt (usually c = 10^{-4} M). After each addition, the variation of chemical shift experienced by the proton signals of the N-methylpyridinium (NMP) cation was monitored and plotted *via* a NMR version of the Benesi–Hildebrand equation.[1]

$$1/\Delta\delta = 1/(K_{ass}\Delta\delta_{max}\,[H]_o) + 1/\Delta\delta_{max}$$

where $\Delta\delta = (\delta_G - \delta_{obs})$, and $\Delta\delta_{max} = (\delta_G - \delta_{HG})$, $[H]_0$ = known total concentration of host, K_{ass} = association constants, δ_G = chemical shift of a nucleus in the guest molecule, δ_{HG} = chemical shift of a nucleus in the host–guest complex, δ_{obs} = an experimentally measured chemical shift

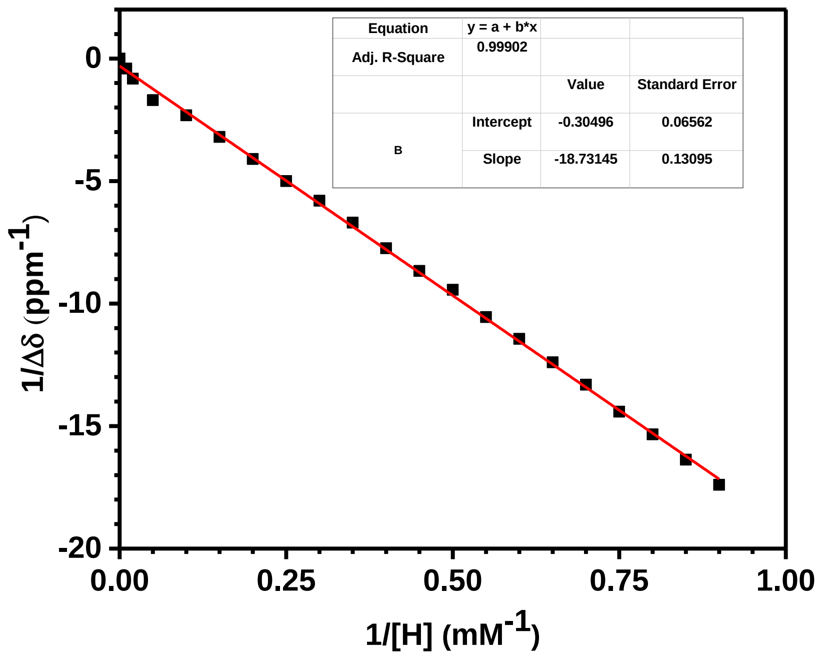
Equation
y = a + b*x
Adj. R-Square
0.99902
Value
Standard Error
B
Intercept
-0.30496
0.06562
Slope
-18.73145
0.13095
1/Δδ (ppm-1)
1/[H] (mM-1)
0
-5
-10
-15
-20
0.00
0.25
0.50
0.75
1.00

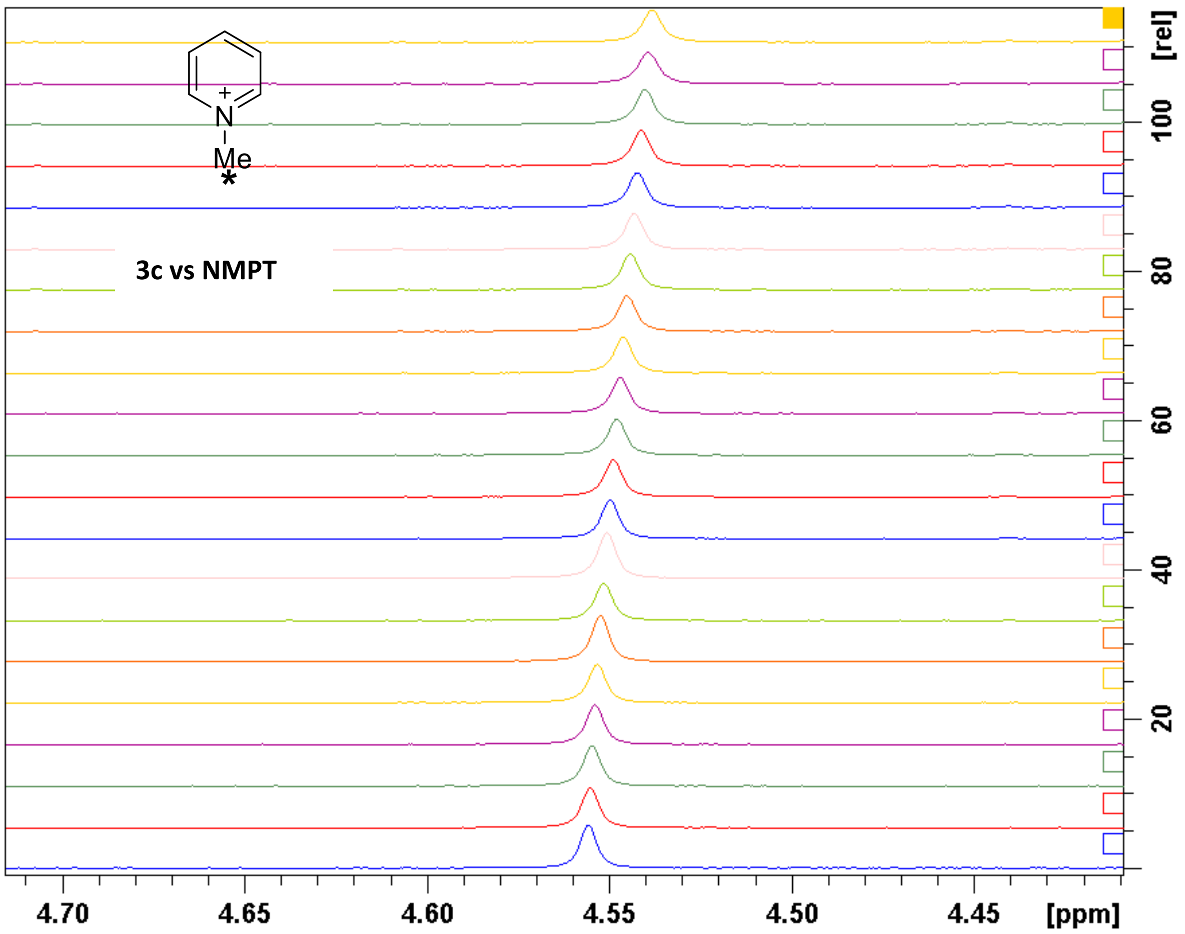
N
Me
*
3c vs NMPT
[rel]
100
80
60
40
20
4.70
4.65
4.60
4.55
4.50
4.45
[ppm]

6. Calculation of Quantum Yield

Fluorescence quantum yields (Φ) were estimated by integrating the area under the fluorescence curves using the equation.

$$\Phi_S = \Phi_R A_S \eta_S / A_R \eta_R$$

Where A was the area under the fluorescence spectral curve and η was the refractive indices of the solvent. Coumarin (Φ 0.73 in EtOAc) was used as quantum yield standard for measuring the quantum yields.

Reference:

1. Fielding, L. Determination of Association Constants (K_{ass}) from Solution NMR Data. *Tetrahedron* **2000**, *56*, 6151-6170.
